# Supplementary material for: Relationship Between RAP and Multi-Modal Cerebral Physiological Dynamics in Moderate/Severe Acute Traumatic Neural Injury: A CAHR-TBI Multivariate Analysis
Source: Bioengineering (Basel). 2025 Sep 22;12(9):1006. doi: 10.3390/bioengineering12091006 (PMC12467443; doi:10.3390/bioengineering12091006)
Supplement: Supplementary file 1 [file bioengineering-12-01006-s001.zip › bioengineering-3783669-Supplemantray.pdf]

Supplementary A: General descriptive relationships between RAP and other physiological variables

This supplemantray presents scatterplots and box plots, along with the median physiological measurements and formal comparison results across different groups segmented based on RAP thresholds. Additionally, it includes a reverse analysis, examining the response of RAP through median measurements and statistical comparisons in relation to other physiological thresholds. This supplemantray also includes %time spent within certain threshold ranges.

AMP, pulse amplitude of ICP; COx_L, cerebral oxygenation index of left hemisphere; COx_R, cerebral oxygenation index of right hemisphere; COx-a_L, COx with ABP of left hemisphere; COx-a_R, COx with ABP of the right hemisphere; CPP, cerebral perfusion pressure; ICP, intracranial pressure; IQR, interquartile range; MAP, mean arterial pressure; PAx, pulse amplitude index; PbtO$_2$*, brain tissue oxygenation ; PRx, pressure reactivity index; RAC, a cerebral autoregulation index; RAP, index of cerebral compensatory reserve; rSO*$_2$*_L, regional cerebral oxygen saturation of left hemisphere; rSO*$_2$*_R, regional cerebral oxygen saturation of the right hemisphere.*

Figure S.1 Scatterplots across the entire population with piecewise linear regression (based on RAP thresholds) for the physiological variables at minute-by-minute resolution

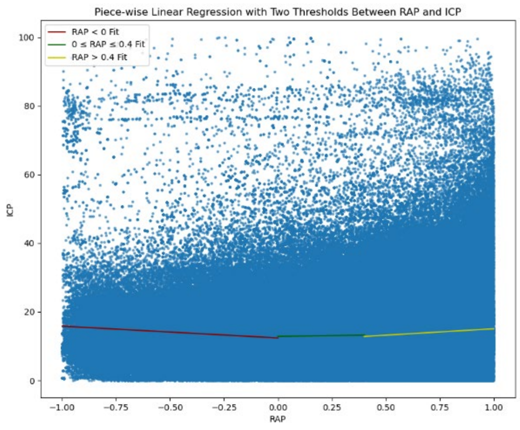

(a) RAP-ICP scatterplot

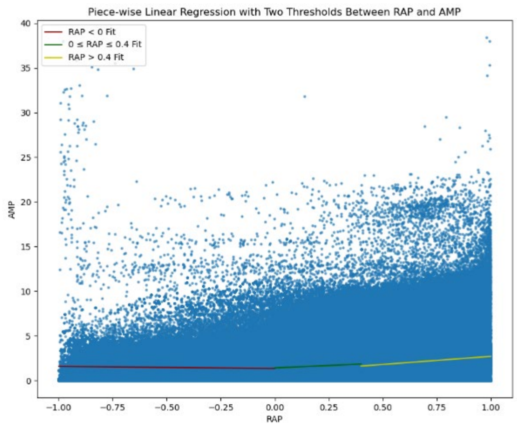

(b) RAP-AMP scatterplot

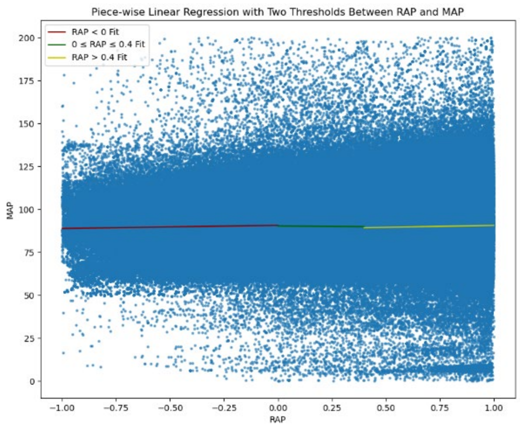

(c) RAP-MAP scatterplot

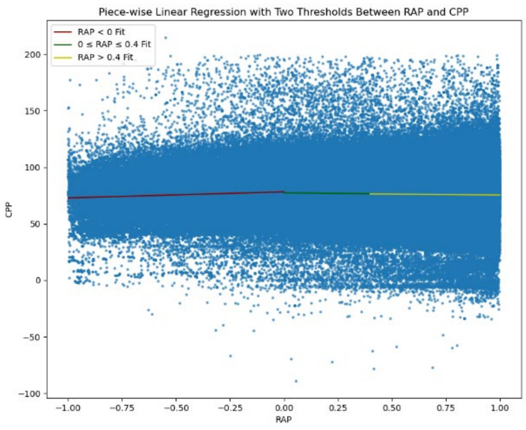

(d) RAP-CPP scatterplot

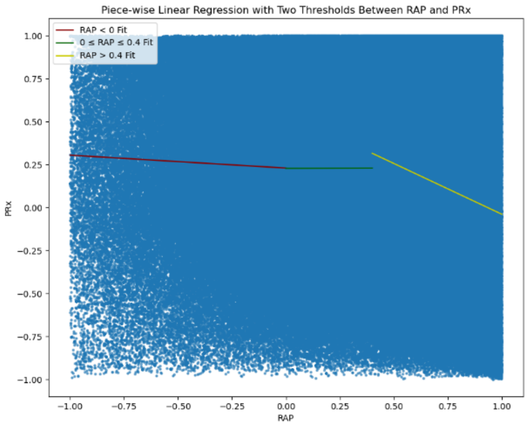

(e) RAP-PRx scatterplot

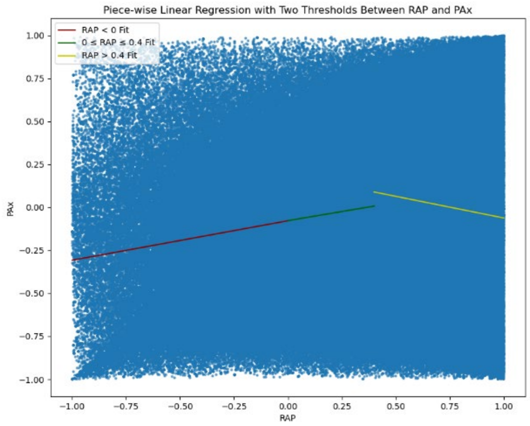

(f) RAP-PAx scatterplot

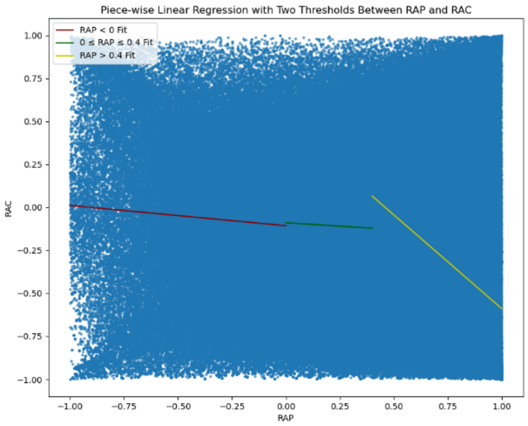

(g) RAP-RAC scatterplot

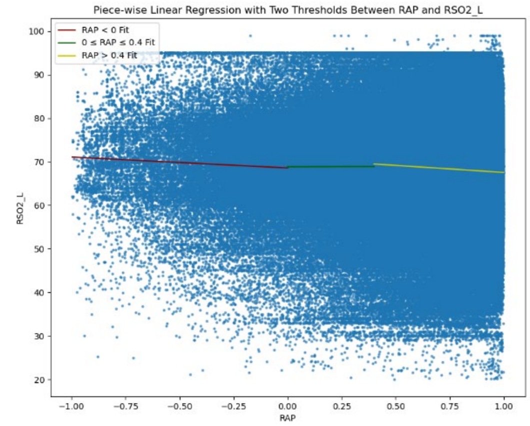

(h) RAP-rSO2_L scatterplot

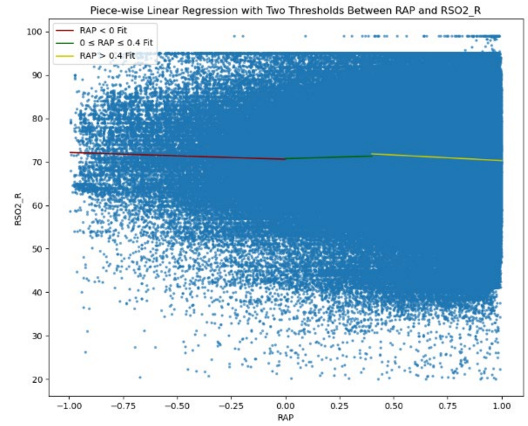

(i) RAP-rSO2_R scatterplot

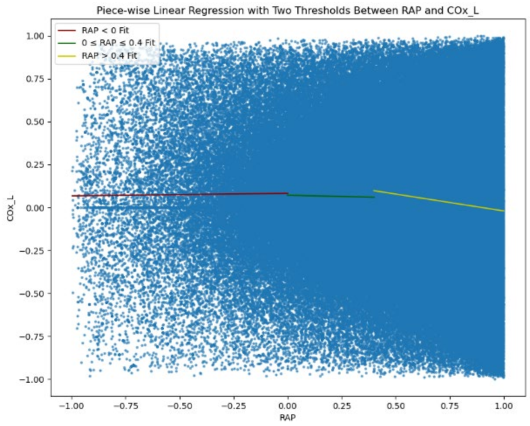

(j) RAP-COx_L scatterplot

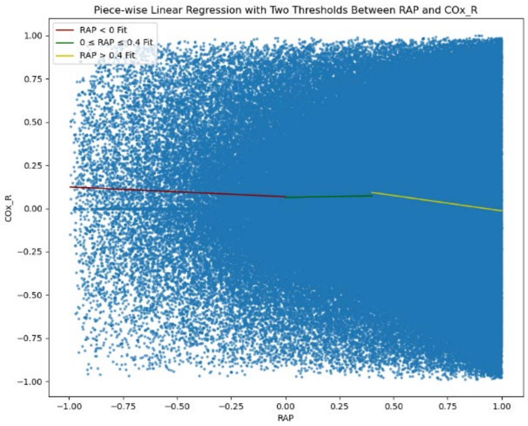

(k) RAP-COx_R scatterplot

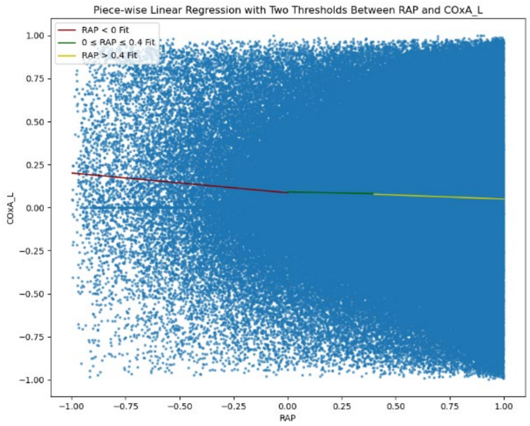

(l) RAP-COxA_L scatterplot

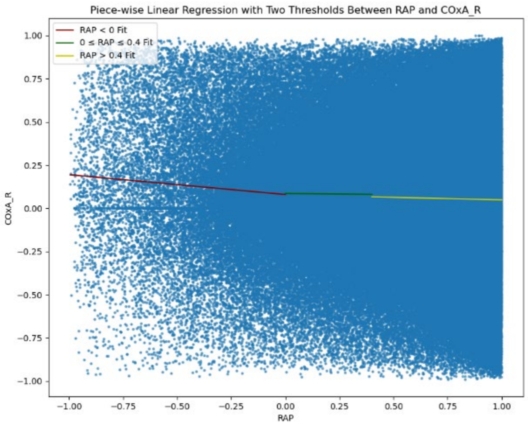

(m) RAP-COxA_R scatterplot

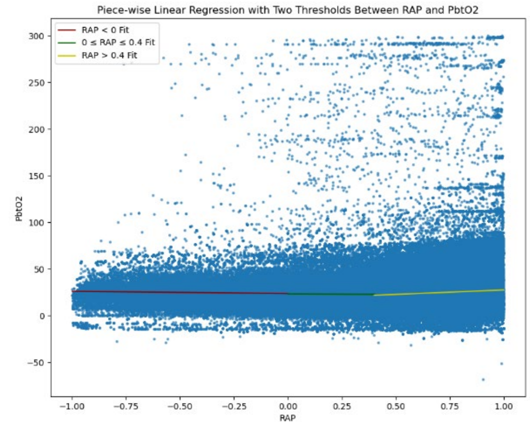

(n) RAP-PbtO2 scatterplot

This figure documents the scatterplots for the physiologic signals (a) ICP (b) AMP (c) MAP (d) CPP (e) PRx (f) PAx (g) RAC (h) rSO_2_L (i) rSO_2_R (j) COx_L (k) COx_L (l) COxA_L (m) COxA_R (n) $PbtO_2$ at the minute-by-minute resolution with respect to RAP across the whole population with piecewise linear regression, which was derived based on RAP threshold ranges. The green, yellow and red solid lines correspond to the $0 \leq RAP \leq 0.4$, $RAP > 0.4$, and $RAP < 0$ segments, respectively.

Figure S.2 Boxplots across the entire population for the physiological variables

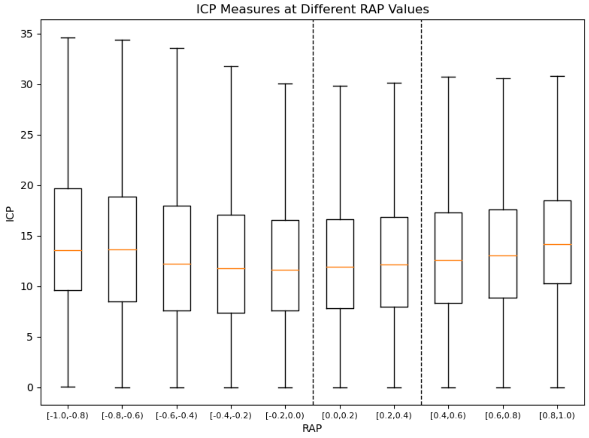

(a) RAP-ICP boxplot

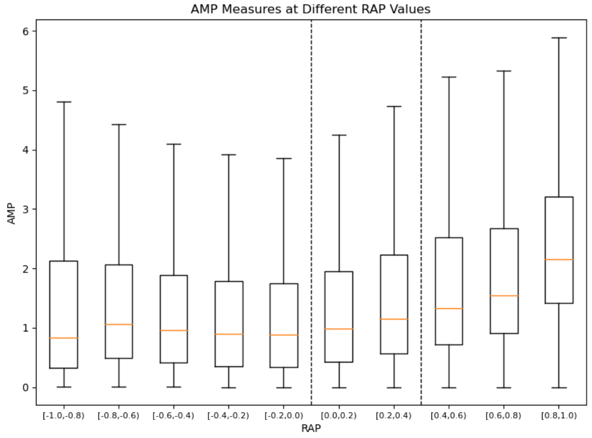

(b) RAP-AMP boxplot

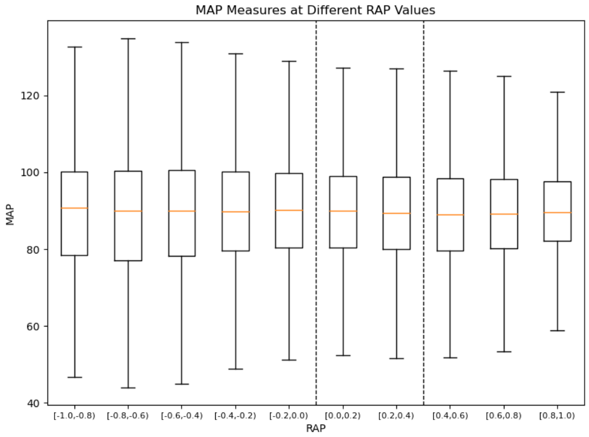

(c) RAP-MAP boxplot

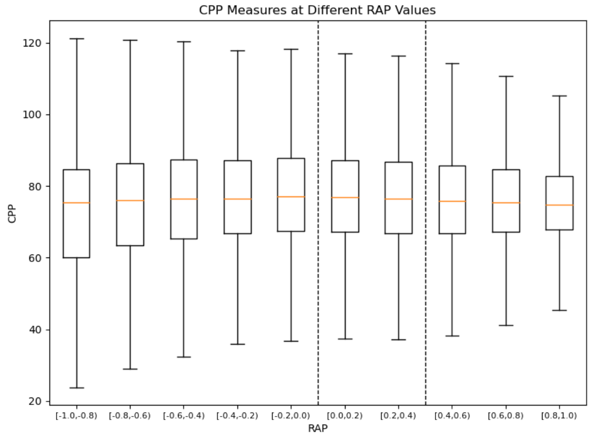

(d) RAP-CPP boxplot

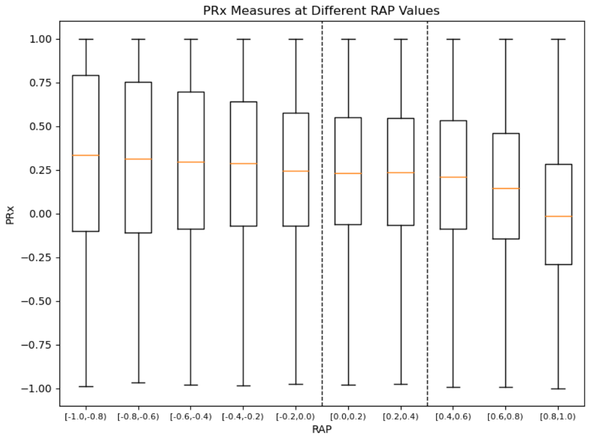

(e) RAP-PRx boxplot

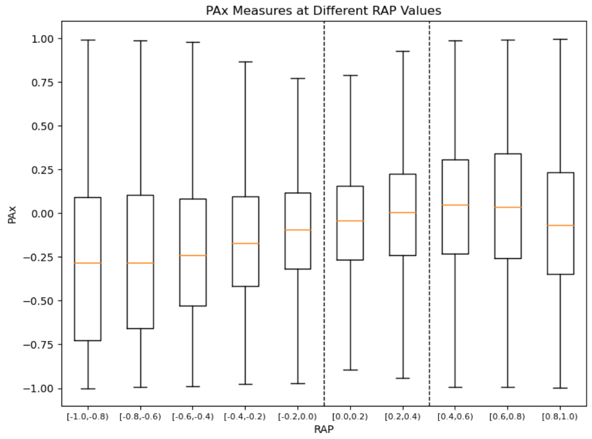

(f) RAP-PAx boxplot

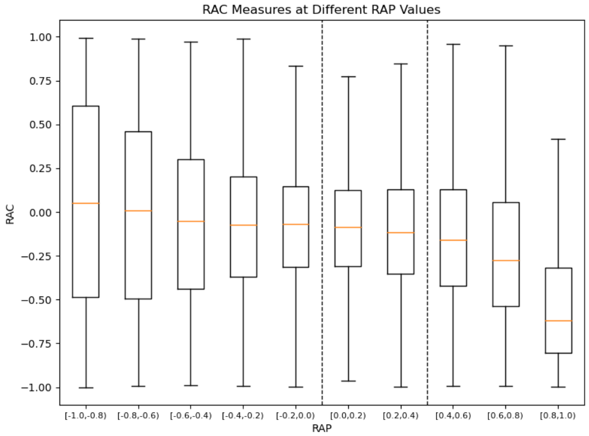

(g) RAP-RAC boxplot

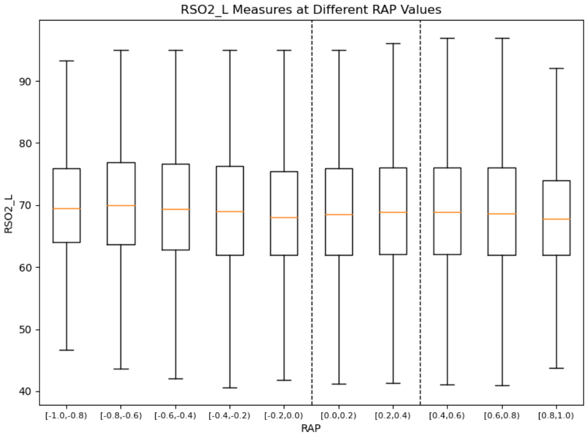

(h) RAP-rSO2_L boxplot

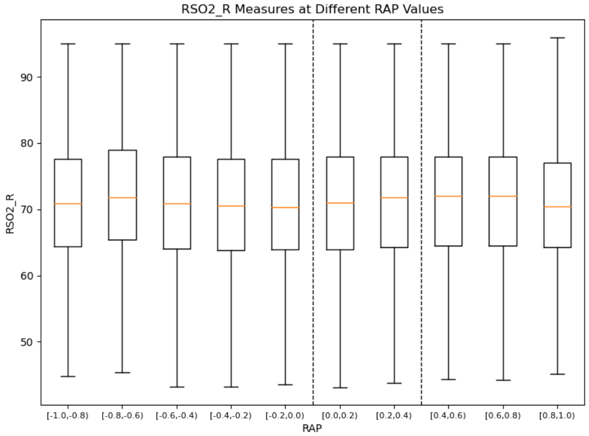

(i) RAP-rSO2_R boxplot

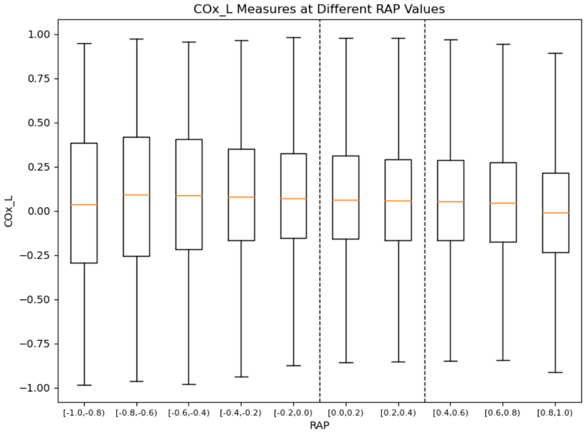

(j) RAP-Cox_L boxplot

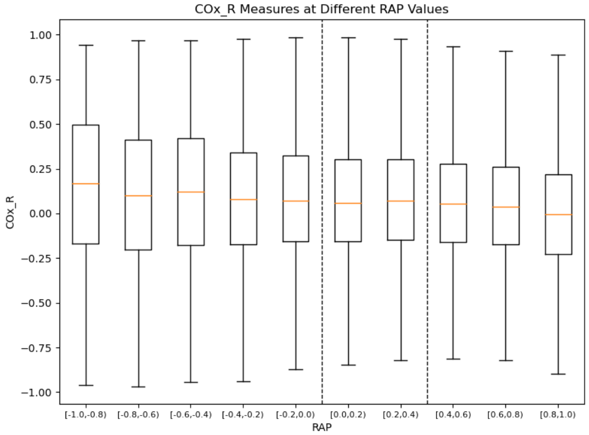

(k) RAP-Cox_R boxplot

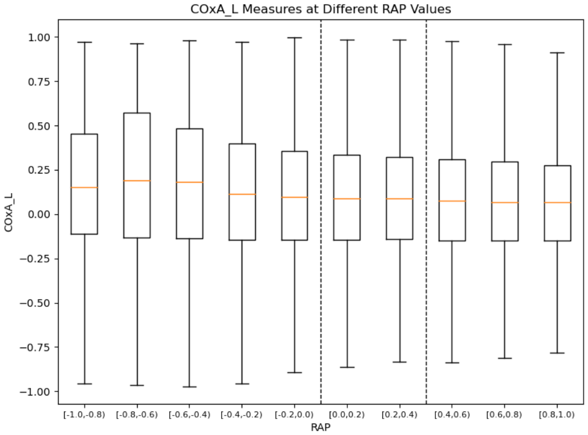

(l) RAP-CoxA_L boxplot

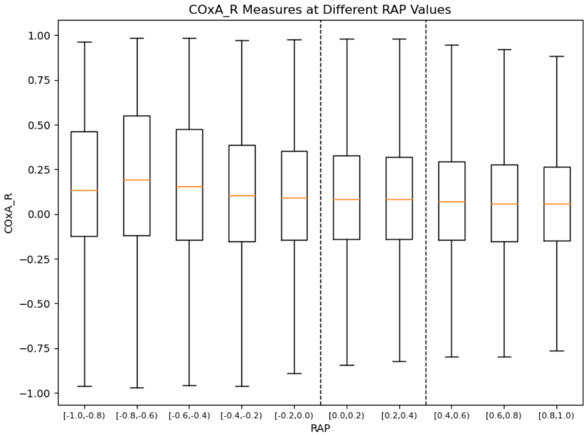

(k) RAP-CoxA_R boxplot

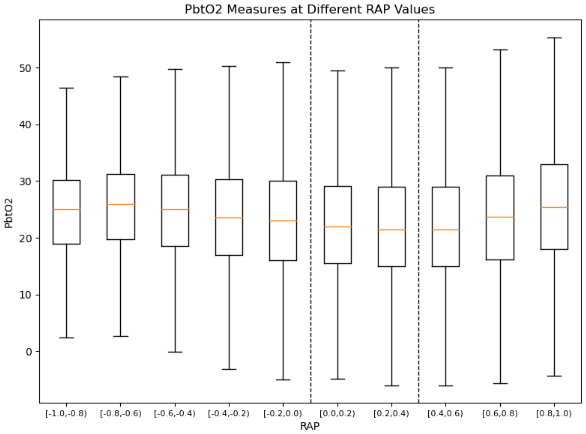

(l) RAP-PbtO2 boxplot

This figure documents the boxplots for the physiologic signals (a) ICP (b) AMP (c) MAP (d) CPP (e) PRx (f) PAx (g) RAC (h) rSO2_L (i) rSO2_R (j) COx_L (k) COx_L (l) COxA_L (m) COxA_R (n) PbtO2 with respect to RAP across the whole population. Each bin covers the 0.2 range of RAP, starting from [-1, -0.8] to [0.8, 1]. The entire plot is segmented into three parts based on the RAP threshold ranges, which are separated by vertical dashed lines.

Table S.1 Median physiology measures across the RAP states with formal comparison and % time spent of parameters within different RAP states at their threshold ranges

**RAP Thresholds**				
**Parameter**	**RAP < 0 (IQR)**	**0 ≤ RAP ≤ 0.4 (IQR)**	**RAP > 0.4 (IQR)**	**p-value**
RAP	-0.234(-0.458—-0.1)	0.239(0.134—0.326)	0.873(0.722—0.949)	**3.05e-19**
ICP	12.03(7.7—17.3)	12.06(7.891—16.75)	13.71(9.697—18.14)	**0.021**
AMP	0.913(0.362—1.822)	1.081(0.506—2.119)	1.93(1.174—3.052)	**3.94e-09**
MAP	90.06(79.64—100.1)	89.66(80.11—98.9)	89.46(81.44—97.85)	**0.008**
CPP	76.69(66.38—87.2)	76.63(67.04—86.9)	75.07(67.58—83.53)	0.638
PRx	0.271(-0.078—0.638)	0.233(-0.064—0.548)	0.047(-0.238—0.36)	**0.035**
PAx	-0.147(-0.423—0.107)	-0.015(-0.25—0.2)	-0.03(-0.314—0.272)	0.184
RAC	-0.06(-0.364—0.214)	-0.103(-0.336—0.126)	-0.485(-0.739—-0.131)	**1.00e-04**
RSO2_L	69.29(62.67—76.485)	69.03(62.54—76.39)	68.02(62.08—74.94)	0.093
RSO2_R	71.01(64.325—78.32)	71.96(64.53—78.16)	71.29(64.57—77.71)	0.152
COx_L	0.076(-0.172—0.35)	0.058(-0.159—0.298)	0.009(-0.215—0.234)	0.170
COx_R	0.085(-0.166—0.352)	0.066(-0.15—0.301)	0.013(-0.206—0.233)	0.508
COxA_L	0.115(-0.14—0.402)	0.085(-0.14—0.323)	0.065(-0.15—0.28)	0.669
COxA_R	0.11(-0.141—0.395)	0.084(-0.138—0.32)	0.059(-0.146—0.268)	0.827
PbtO2	24.0(17.31—30.56)	21.66(15.01—29.0)	24.67(17.24—32.0)	0.819

% time spent within the thresholds of the parameters							
	RAP < 0 (IQR)		0 ≤ RAP ≤ 0.4 (IQR)		RAP > 0.4 (IQR)		
% time spent within ICP thresholds	ICP < 20	ICP ≥ 20	ICP < 20	ICP ≥ 20	ICP < 20	ICP ≥ 20	
	91.29%(75.06%—99.03%)	8.71%(0.97%—24.94%)	94.11%(79.5%—99.14%)	5.89%(0.86%—20.5%)	95.07%(80.42%—99.38%)	4.93%(0.62%—19.58%)	
	ICP < 22	ICP ≥ 22	ICP < 22	ICP ≥ 22	ICP < 22	ICP ≥ 22	
	95.5%(84.62%—100.0%)	4.5%(0.0%—15.38%)	96.98%(89.06%—99.69%)	3.02%(0.31%—10.94%)	97.92%(89.57%—99.8%)	2.08%(0.2%—10.43%)	
% time spent within CPP thresholds	CPP < 60	CPP ≥ 60	CPP < 60	CPP ≥ 60	CPP < 60	CPP ≥ 60	
	3.72%(0.55%—11.03%)	96.28%(88.97%—99.45%)	3.66%(0.89%—9.38%)	96.34%(90.62%—99.11%)	4.54%(1.21%—9.35%)	95.46%(90.65%—98.79%)	
	CPP < 70	CPP ≥ 70	CPP < 70	CPP ≥ 70	CPP < 70	CPP ≥ 70	
	24.19%(9.65%—48.57%)	75.81%(51.43%—90.35%)	24.41%(9.38%—47.42%)	75.59%(52.58%—90.62%)	34.25%(14.63%—54.07%)	65.75%(45.93%—85.37%)	
% time spent within PRx thresholds	PRx < 0	PRx ≥ 0	PRx < 0	PRx ≥ 0	PRx < 0	PRx ≥ 0	
	25.83%(13.29%—39.79%)	74.17%(60.21%—86.71%)	27.27%(16.67%—39.54%)	72.73%(60.46%—83.33%)	42.11%(25.78%—55.92%)	57.89%(44.08%—74.22%)	
	PRx < 0.25	PRx ≥ 0.25	PRx < 0.25	PRx ≥ 0.25	PRx < 0.25	PRx ≥ 0.25	
	45.11%(27.25%—61.72%)	54.89%(38.28%—72.75%)	50.91%(33.8%—65.83%)	49.09%(34.17%—66.2%)	67.84%(49.99%—78.08%)	32.16%(21.92%—50.01%)	
	PRx < 0.35	PRx ≥ 0.35	PRx < 0.35	PRx ≥ 0.35	PRx < 0.35	PRx ≥ 0.35	
	52.93%(34.72%—69.48%)	47.07%(30.52%—65.28%)	61.0%(44.16%—74.54%)	39.0%(25.46%—55.84%)	75.66%(60.52%—84.96%)	24.34%(15.04%—39.48%)	
% time spent within PAx thresholds	PAx < 0	PAx ≥ 0	PAx < 0	PAx ≥ 0	PAx < 0	PAx ≥ 0	
	65.87%(52.96%—80.68%)	34.13%(19.32%—47.04%)	53.61%(41.02%—66.4%)	46.39%(33.6%—58.98%)	49.26%(32.33%—64.97%)	50.74%(35.03%—67.67%)	
	PAx < 0.25	PAx ≥ 0.25	PAx < 0.25	PAx ≥ 0.25	PAx < 0.25	PAx ≥ 0.25	

	86.86%(77.61%—93.26%)	13.14%(6.74%—22.39%)	83.94%(71.73%—89.42%)	16.06%(10.58%—28.27%)	75.49%(59.28%—85.6%)	24.51%(14.4%—40.72%)	
% time spent within RAC thresholds	RAC < 0	RAC ≥ 0	RAC < 0	RAC ≥ 0	RAC < 0	RAC ≥ 0	
	53.42%(41.0%—71.98%)	46.58%(28.02%—59.0%)	65.0%(51.54%—77.42%)	35.0%(22.58%—48.46%)	81.76%(66.19%—92.08%)	18.24%(7.92%—33.81%)	
% time spent within rSO_2_L thresholds	rSO_2_L < 60	rSO_2_L ≥ 60	rSO_2_L < 60	rSO_2_L ≥ 60	rSO_2_L < 60	rSO_2_L ≥ 60	
	7.65%(0.0%—36.09%)	92.35%(63.91%—100.0%)	7.25%(0.07%—37.7%)	92.75%(62.3%—99.93%)	6.15%(0.21%—39.16%)	93.85%(60.84%—99.79%)	
	rSO_2_L < 70	rSO_2_L ≥ 70	rSO_2_L < 70	rSO_2_L ≥ 70	rSO_2_L < 70	rSO_2_L ≥ 70	
	60.43%(14.12%—94.57%)	39.57%(5.43%—85.88%)	61.73%(15.01%—94.46%)	38.27%(5.54%—84.99%)	63.33%(16.83%—95.02%)	36.67%(4.98%—83.17%)	
	rSO_2_L < 80	rSO_2_L ≥ 80	rSO_2_L < 80	rSO_2_L ≥ 80	rSO_2_L < 80	rSO_2_L ≥ 80	
	98.18%(80.61%—100.0%)	1.82%(0.0%—19.39%)	99.06%(82.05%—100.0%)	0.94%(0.0%—17.95%)	99.36%(84.1%—100.0%)	0.64%(0.0%—15.9%)	
	rSO_2_L < 90	rSO_2_L ≥ 90	rSO_2_L < 90	rSO_2_L ≥ 90	rSO_2_L < 90	rSO_2_L ≥ 90	
	100.0%(100.0%—100.0%)	0.0%(0.0%—0.0%)	100.0%(100.0%—100.0%)	0.0%(0.0%—0.0%)	100.0%(99.99%—100.0%)	0.0%(0.0%—0.01%)	
% time spent within rSO_2_R thresholds	rSO_2_R < 60	rSO_2_R ≥ 60	rSO_2_R < 60	rSO_2_R ≥ 60	rSO_2_R < 60	rSO_2_R ≥ 60	
	3.06%(0.0%—25.5%)	96.94%(74.5%—100.0%)	3.38%(0.0%—31.1%)	96.62%(68.9%—100.0%)	3.49%(0.0%—28.1%)	96.51%(71.9%—100.0%)	
	rSO_2_R < 70	rSO_2_R ≥ 70	rSO_2_R < 70	rSO_2_R ≥ 70	rSO_2_R < 70	rSO_2_R ≥ 70	
	44.22%(9.72%—87.34%)	55.78%(12.66%—90.28%)	40.8%(12.16%—89.35%)	59.2%(10.65%—87.84%)	50.97%(11.12%—91.85%)	49.03%(8.15%—88.88%)	
	rSO_2_R < 80	rSO_2_R ≥ 80	rSO_2_R < 80	rSO_2_R ≥ 80	rSO_2_R < 80	rSO_2_R ≥ 80	
	97.88%(74.32%—100.0%)	2.12%(0.0%—25.68%)	98.16%(78.55%—100.0%)	1.84%(0.0%—21.45%)	98.57%(80.81%—100.0%)	1.43%(0.0%—19.19%)	
	rSO_2_R < 90	rSO_2_R ≥ 90	rSO_2_R < 90	rSO_2_R ≥ 90	rSO_2_R < 90	rSO_2_R ≥ 90	
	100.0%(100.0%—100.0%)	0.0%(0.0%—0.0%)	100.0%(99.97%—100.0%)	0.0%(0.0%—0.03%)	100.0%(99.77%—100.0%)	0.0%(0.0%—0.23%)	

% time spent within COx_L thresholds	COx_L < 0	COx_L ≥ 0	COx_L < 0	COx_L ≥ 0	COx_L < 0	COx_L ≥ 0	
	38.32%(30.12%—48.87%)	61.68%(51.13%—69.88%)	38.92%(29.21%—47.51%)	61.08%(52.49%—70.79%)	42.33%(33.83%—52.61%)	57.67%(47.39%—66.17%)	
	COx_L < 0.20	COx_L ≥ 0.20	COx_L < 0.20	COx_L ≥ 0.20	COx_L < 0.20	COx_L ≥ 0.20	
	66.67%(56.83%—77.69%)	33.33%(22.31%—43.17%)	67.15%(58.93%—75.12%)	32.85%(24.88%—41.07%)	75.11%(68.12%—80.54%)	24.89%(19.46%—31.88%)	
% time spent within COx_R thresholds	COx_R < 0	COx_R ≥ 0	COx_R < 0	COx_R ≥ 0	COx_R < 0	COx_R ≥ 0	
	38.34%(28.28%—47.89%)	61.66%(52.11%—71.72%)	36.2%(27.79%—46.44%)	63.8%(53.56%—72.21%)	42.22%(31.91%—50.3%)	57.78%(49.7%—68.09%)	
	COx_R < 0.20	COx_R ≥ 0.20	COx_R < 0.20	COx_R ≥ 0.20	COx_R < 0.20	COx_R ≥ 0.20	
	67.29%(55.33%—76.51%)	32.71%(23.49%—44.67%)	70.02%(59.33%—78.93%)	29.98%(21.07%—40.67%)	76.26%(68.38%—83.12%)	23.74%(16.88%—31.62%)	
% time spent within COx-a_L thresholds	COx-a_L < 0	COx-a_L ≥ 0	COx-a_L < 0	COx-a_L ≥ 0	COx-a_L < 0	COx-a_L ≥ 0	
	36.63%(26.67%—46.05%)	63.37%(53.95%—73.33%)	35.88%(28.99%—46.28%)	64.12%(53.72%—71.01%)	36.34%(30.42%—44.95%)	63.66%(55.05%—69.58%)	
	COx-a_L < 0.20	COx-a_L ≥ 0.20	COx-a_L < 0.20	COx-a_L ≥ 0.20	COx-a_L < 0.20	COx-a_L ≥ 0.20	
	62.04%(50.0%—71.4%)	37.96%(28.6%—50.0%)	64.0%(55.23%—74.35%)	36.0%(25.65%—44.77%)	69.36%(61.77%—76.1%)	30.64%(23.9%—38.23%)	
% time spent within COx-a_R thresholds	COx-a_R < 0	COx-a_R ≥ 0	COx-a_R < 0	COx-a_R ≥ 0	COx-a_R < 0	COx-a_R ≥ 0	
	35.29%(26.05%—47.33%)	64.71%(52.67%—73.95%)	35.33%(28.0%—45.16%)	64.67%(54.84%—72.0%)	38.46%(28.52%—44.81%)	61.54%(55.19%—71.48%)	
	COx-a_R < 0.20	COx-a_R ≥ 0.20	COx-a_R < 0.20	COx-a_R ≥ 0.20	COx-a_R < 0.20	COx-a_R ≥ 0.20	
	64.73%(50.06%—74.24%)	35.27%(25.76%—49.94%)	68.08%(54.55%—75.64%)	31.92%(24.36%—45.45%)	72.16%(63.9%—80.09%)	27.84%(19.91%—36.1%)	
% time spent within $PbtO_2$ thresholds	$PbtO_2$ < 15	$PbtO_2$ ≥ 15	$PbtO_2$ < 15	$PbtO_2$ ≥ 15	$PbtO_2$ < 15	$PbtO_2$ ≥ 15	
	12.37%(2.69%—65.69%)	87.63%(34.31%—97.31%)	12.42%(1.98%—69.96%)	87.58%(30.04%—98.02%)	7.57%(1.41%—70.3%)	92.43%(29.7%—98.59%)	
	$PbtO_2$ < 25	$PbtO_2$ ≥ 25	$PbtO_2$ < 25	$PbtO_2$ ≥ 25	$PbtO_2$ < 25	$PbtO_2$ ≥ 25	

	63.94%(23.49%—94.08%)	36.06%(5.92%—76.51%)	67.7%(32.68%—95.37%)	32.3%(4.63%—67.32%)	67.09%(20.1%—96.55%)	32.91%(3.45%—79.9%)	

Table S.2 Median physiology measures across the thresholds of different physiologic variables with formal comparison and % time spent of RAP within different RAP states at the threshold ranges

**ICP Thresholds**							
**Parameter**	**ICP < 20**			**ICP ≥ 20**			**p-value**
ICP	12.11(8.437—15.49)			23.64(21.5—27.58)			**9.83e-20**
RAP	0.812(0.525—0.933)			0.853(0.594—0.95)			0.535
% time spent within RAP thresholds	RAP < 0	0 ≤ RAP ≤ 0.4	RAP > 0.4	RAP < 0	0 ≤ RAP ≤ 0.4	RAP > 0.4	
	4.69%(2.06%—10.26%)	10.77%(6.05%—17.69%)	83.93%(72.05%—91.68%)	6.25%(2.2%—14.24%)	10.34%(4.59%—17.93%)	81.33%(67.68%—92.42%)	
**Parameter**	**ICP < 22**			**ICP ≥ 22**			**p-value**
ICP	12.57(8.785—16.37)			26.25(24.01—30.89)			**5.23e-11**
RAP	0.816(0.532—0.935)			0.845(0.579—0.948)			0.725
% time spent within RAP thresholds	RAP < 0	0 ≤ RAP ≤ 0.4	RAP > 0.4	RAP < 0	0 ≤ RAP ≤ 0.4	RAP > 0.4	
	4.66%(2.16%—9.97%)	10.67%(5.85%—17.44%)	83.92%(72.19%—91.68%)	6.21%(1.98%—15.52%)	11.14%(4.29%—19.0%)	79.7%(64.38%—92.96%)	
**CPP Thresholds**							
**Parameter**	**CPP < 60**			**CPP ≥ 60**			**p-value**
CPP	54.46(48.67—57.86)			76.78(69.79—85.17)			**8.92e-10**
RAP	0.719(0.406—0.904)			0.83(0.558—0.939)			0.139
% time spent within RAP thresholds	RAP < 0	0 ≤ RAP ≤ 0.4	RAP > 0.4	RAP < 0	0 ≤ RAP ≤ 0.4	RAP > 0.4	
	4.36%(1.15%—11.03%)	9.66%(3.71%—20.0%)	83.67%(67.98%—93.76%)	4.79%(2.23%—9.43%)	10.33%(5.65%—17.65%)	84.1%(72.68%—92.06%)	
**Parameter**	**CPP < 70**			**CPP ≥ 70**			**p-value**
CPP	64.05(59.04—67.29)			80.68(75.15—88.19)			**6.96e-49**
RAP	0.818(0.539—0.934)			0.823(0.543—0.938)			**0.006**
	RAP < 0	0 ≤ RAP ≤ 0.4	RAP > 0.4	RAP < 0	0 ≤ RAP ≤ 0.4	RAP > 0.4	

% time spent within RAP thresholds	3.76%(1.48%—9.11%)	8.48%(3.81%—16.04%)	87.36%(74.43%—94.17%)	5.3%(2.49%—10.4%)	11.23%(6.23%—19.67%)	82.59%(68.22%—91.09%)	
**PRx Thresholds**							
**Parameter**	**PRx < 0**			**PRx ≥ 0**			**p-value**
PRx	-0.262(-0.447—-0.123)			0.35(0.164—0.605)			**1.30e-51**
RAP	0.881(0.675—0.955)			0.761(0.455—0.913)			**1.76e-09**
% time spent within RAP thresholds	RAP < 0	0 ≤ RAP ≤ 0.4	RAP > 0.4	RAP < 0	0 ≤ RAP ≤ 0.4	RAP > 0.4	
	3.12%(1.27%—8.98%)	7.69%(3.68%—15.6%)	88.26%(74.42%—94.77%)	6.12%(3.0%—11.38%)	12.86%(6.88%—20.67%)	80.86%(66.37%—89.69%)	
**Parameter**	**PRx < 0.25**			**PRx ≥ 0.25**			**p-value**
PRx	-0.124(-0.346—0.058)			0.528(0.375—0.74)			**7.99e-50**
RAP	0.863(0.633—0.949)			0.721(0.4—0.895)			**6.86e-05**
% time spent within RAP thresholds	RAP < 0	0 ≤ RAP ≤ 0.4	RAP > 0.4	RAP < 0	0 ≤ RAP ≤ 0.4	RAP > 0.4	
	3.51%(1.47%—8.51%)	8.77%(4.65%—16.8%)	87.33%(73.38%—93.42%)	8.84%(4.18%—15.44%)	15.52%(8.5%—22.38%)	75.33%(60.93%—85.68%)	
**Parameter**	**PRx < 0.35**			**PRx ≥ 0.35**			**p-value**
PRx	-0.081(-0.316—0.122)			0.605(0.464—0.792)			**4.74e-36**
RAP	0.856(0.619—0.947)			0.701(0.377—0.886)			**9.47e-06**
% time spent within RAP thresholds	RAP < 0	0 ≤ RAP ≤ 0.4	RAP > 0.4	RAP < 0	0 ≤ RAP ≤ 0.4	RAP > 0.4	
	4.36%(1.15%—11.03%)	9.66%(3.71%—20.0%)	83.67%(67.98%—93.76%)	4.79%(2.23%—9.43%)	10.33%(5.65%—17.65%)	84.1%(72.68%—92.06%)	
**PAx Thresholds**							
**Parameter**	**PAx < 0**			**PAx ≥ 0**			**p-value**
PAx	-0.293(-0.486—-0.141)			0.275(0.128—0.477)			**5.89e-63**
RAP	0.842(0.54—0.945)			0.798(0.543—0.925)			0.257
% time spent within RAP thresholds	RAP < 0	0 ≤ RAP ≤ 0.4	RAP > 0.4	RAP < 0	0 ≤ RAP ≤ 0.4	RAP > 0.4	
	6.7%(3.12%—14.18%)	11.46%(6.1%—19.23%)	81.07%(64.03%—89.64%)	3.3%(1.5%—6.96%)	10.23%(5.22%—17.72%)	85.85%(75.64%—92.76%)	
**Parameter**	**PAx < 0.25**			**PAx ≥ 0.25**			**p-value**

PAx	-0.17(-0.4—0.029)			0.457(0.342—0.615)			**4.13e-29**
RAP	0.828(0.518—0.94)			0.804(0.587—0.924)			0.215
% time spent within RAP thresholds	RAP < 0	0 ≤ RAP ≤ 0.4	RAP > 0.4	RAP < 0	0 ≤ RAP ≤ 0.4	RAP > 0.4	
	5.92%(2.67%—12.04%)	12.04%(6.07%—20.37%)	81.31%(66.25%—90.92%)	2.76%(1.15%—6.59%)	7.92%(3.93%—14.97%)	88.85%(78.44%—94.53%)	
**RAC Thresholds**							
**Parameter**	**RAC < 0**			**RAC ≥ 0**			**p-value**
RAC	-0.546(-0.76—-0.298)			0.247(0.109—0.449)			**1.64e-31**
RAP	0.865(0.639—0.95)			0.608(0.278—0.817)			**6.99e-08**
% time spent within RAC thresholds	RAP < 0	0 ≤ RAP ≤ 0.4	RAP > 0.4	RAP < 0	0 ≤ RAP ≤ 0.4	RAP > 0.4	
	3.73%(1.56%—8.56%)	9.0%(4.52%—15.8%)	86.36%(74.79%—93.53%)	10.54%(5.59%—19.46%)	17.62%(11.01%—25.66%)	69.39%(54.07%—80.8%)	
**rSO_2_L Thresholds**							
**Parameter**	**rSO_2_L < 60**			**rSO_2_L ≥ 60**			**p-value**
rSO_2_L	54.89(49.31—57.96)			71.0(66.34—76.44)			**1.01e-13**
RAP	0.856(0.623—0.941)			0.846(0.585—0.945)			0.973
% time spent within RAP thresholds	RAP < 0	0 ≤ RAP ≤ 0.4	RAP > 0.4	RAP < 0	0 ≤ RAP ≤ 0.4	RAP > 0.4	
	2.84%(0.47%—10.01%)	8.66%(2.13%—18.37%)	85.76%(70.49%—96.75%)	4.93%(2.4%—10.93%)	10.32%(5.33%—17.25%)	84.17%(71.05%—91.67%)	
**Parameter**	**rSO_2_L < 70**			**rSO_2_L ≥ 70**			**p-value**
rSO_2_L	63.29(58.11—66.91)			75.42(71.63—79.97)			**1.43e-24**
RAP	0.864(0.622—0.949)			0.824(0.554—0.937)			**0.044**
% time spent within RAP thresholds	RAP < 0	0 ≤ RAP ≤ 0.4	RAP > 0.4	RAP < 0	0 ≤ RAP ≤ 0.4	RAP > 0.4	
	3.49%(1.44%—8.83%)	8.67%(3.82%—19.34%)	86.18%(72.84%—93.69%)	5.76%(2.18%—11.85%)	11.51%(4.89%—20.15%)	82.2%(65.31%—89.78%)	
**Parameter**	**rSO_2_L < 80**			**rSO_2_L ≥ 80**			**p-value**
rSO_2_L	67.99(62.0—72.13)			84.03(81.67—88.6)			**2.49e-11**
RAP	0.858(0.607—0.948)			0.767(0.499—0.904)			0.097
% time spent within RAP thresholds	RAP < 0	0 ≤ RAP ≤ 0.4	RAP > 0.4	RAP < 0	0 ≤ RAP ≤ 0.4	RAP > 0.4	
	4.63%(2.14%—10.22%)	9.37%(4.9%—17.73%)	84.8%(72.34%—92.33%)	6.06%(2.01%—13.87%)	13.7%(6.71%—23.28%)	78.68%(59.04%—88.96%)	

<table>
<tr><td colspan="8"></td></tr>
<tr><th>Parameter</th><th colspan="3">rSO₂_L < 90</th><th colspan="3">rSO₂_L ≥ 90</th><th>p-value</th></tr>
<tr><td>rSO_2_L</td><td colspan="3">69.08(62.94—74.5)</td><td colspan="3">94.35(91.99—94.99)</td><td>0.003</td></tr>
<tr><td>RAP</td><td colspan="3">0.851(0.595—0.945)</td><td colspan="3">0.75(0.466—0.875)</td><td>0.328</td></tr>
<tr><td rowspan="2">% time spent within RAP thresholds</td><td>RAP < 0</td><td>0 ≤ RAP ≤ 0.4</td><td>RAP > 0.4</td><td>RAP < 0</td><td>0 ≤ RAP ≤ 0.4</td><td>RAP > 0.4</td><td rowspan="2"></td></tr>
<tr><td>4.75%(2.17%—10.11%)</td><td>10.21%(5.11%—18.45%)</td><td>84.17%(72.01%—91.98%)</td><td>2.16%(0.0%—12.92%)</td><td>8.47%(0.0%—19.51%)</td><td>82.91%(58.58%—100.0%)</td></tr>
<tr><th colspan="8">rSO₂_R Thresholds</th></tr>
<tr><th>Parameter</th><th colspan="3">rSO₂_R < 60</th><th colspan="3">rSO₂_R ≥ 60</th><th>p-value</th></tr>
<tr><td>rSO_2_R</td><td colspan="3">55.36(50.46—58.07)</td><td colspan="3">74.03(67.99—79.0)</td><td>3.20e-09</td></tr>
<tr><td>RAP</td><td colspan="3">0.825(0.558—0.935)</td><td colspan="3">0.845(0.584—0.943)</td><td>0.490</td></tr>
<tr><td rowspan="2">% time spent within RAP thresholds</td><td>RAP < 0</td><td>0 ≤ RAP ≤ 0.4</td><td>RAP > 0.4</td><td>RAP < 0</td><td>0 ≤ RAP ≤ 0.4</td><td>RAP > 0.4</td><td rowspan="2"></td></tr>
<tr><td>3.48%(0.14%—9.49%)</td><td>8.88%(2.14%—25.45%)</td><td>85.07%(51.55%—94.75%)</td><td>4.96%(2.36%—10.27%)</td><td>9.64%(4.53%—18.95%)</td><td>84.28%(69.81%—92.36%)</td></tr>
<tr><td colspan="8"></td></tr>
<tr><th>Parameter</th><th colspan="3">rSO₂_R < 70</th><th colspan="3">rSO₂_R ≥ 70</th><th>p-value</th></tr>
<tr><td>rSO_2_R</td><td colspan="3">63.81(58.99—66.99)</td><td colspan="3">78.0(74.04—80.32)</td><td>4.18e-24</td></tr>
<tr><td>RAP</td><td colspan="3">0.86(0.603—0.949)</td><td colspan="3">0.827(0.565—0.936)</td><td>0.375</td></tr>
<tr><td rowspan="2">% time spent within RAP thresholds</td><td>RAP < 0</td><td>0 ≤ RAP ≤ 0.4</td><td>RAP > 0.4</td><td>RAP < 0</td><td>0 ≤ RAP ≤ 0.4</td><td>RAP > 0.4</td><td rowspan="2"></td></tr>
<tr><td>3.82%(1.79%—10.68%)</td><td>8.53%(3.97%—18.01%)</td><td>86.07%(69.49%—94.03%)</td><td>5.06%(2.52%—11.35%)</td><td>12.77%(5.11%—21.49%)</td><td>80.68%(65.59%—90.98%)</td></tr>
<tr><td colspan="8"></td></tr>
<tr><th>Parameter</th><th colspan="3">rSO₂_R < 80</th><th colspan="3">rSO₂_R ≥ 80</th><th>p-value</th></tr>
<tr><td>rSO_2_R</td><td colspan="3">70.26(63.97—76.16)</td><td colspan="3">84.0(81.67—87.5)</td><td>2.67e-11</td></tr>
<tr><td>RAP</td><td colspan="3">0.853(0.597—0.946)</td><td colspan="3">0.781(0.501—0.911)</td><td>0.002</td></tr>
<tr><td rowspan="2">% time spent within RAP thresholds</td><td>RAP < 0</td><td>0 ≤ RAP ≤ 0.4</td><td>RAP > 0.4</td><td>RAP < 0</td><td>0 ≤ RAP ≤ 0.4</td><td>RAP > 0.4</td><td rowspan="2"></td></tr>
<tr><td>4.75%(2.27%—11.31%)</td><td>8.83%(4.87%—19.22%)</td><td>85.47%(67.95%—91.91%)</td><td>6.71%(3.43%—12.95%)</td><td>11.61%(6.15%—25.61%)</td><td>79.17%(60.59%—88.93%)</td></tr>
<tr><td colspan="8"></td></tr>
<tr><th>Parameter</th><th colspan="3">rSO₂_R < 90</th><th colspan="3">rSO₂_R ≥ 90</th><th>p-value</th></tr>
<tr><td>rSO_2_R</td><td colspan="3">72.0(65.0—78.83)</td><td colspan="3">93.5(91.39—94.99)</td><td>0.003</td></tr>
<tr><td>RAP</td><td colspan="3">0.844(0.584—0.943)</td><td colspan="3">0.757(0.454—0.897)</td><td>0.037</td></tr>
<tr><td></td><td>RAP < 0</td><td>0 ≤ RAP ≤ 0.4</td><td>RAP > 0.4</td><td>RAP < 0</td><td>0 ≤ RAP ≤ 0.4</td><td>RAP > 0.4</td><td></td></tr>
</table>

% time spent within RAP thresholds	4.65%(2.4%—10.78%)	9.54%(5.14%—18.61%)	84.58%(72.38%—92.05%)	5.36%(0.0%—16.67%)	9.53%(0.0%—28.38%)	79.35%(52.9%—100.0%)	
**COx_L Thresholds**							
**Parameter**	**COx_L < 0**			**COx_L ≥ 0**			**p-value**
COx_L	-0.217(-0.379—-0.1)			0.231(0.107—0.414)			**5.90e-23**
RAP	0.87(0.634—0.953)			0.822(0.547—0.934)			**0.023**
% time spent within RAP thresholds	RAP < 0	0 ≤ RAP ≤ 0.4	RAP > 0.4	RAP < 0	0 ≤ RAP ≤ 0.4	RAP > 0.4	
	4.12%(1.99%—10.4%)	9.66%(4.5%—17.03%)	86.33%(73.37%—93.25%)	5.64%(2.55%—10.65%)	11.01%(5.65%—19.87%)	81.92%(68.46%—90.45%)	
**Parameter**	**COx_L < 0.20**			**COx_L ≥ 0.20**			**p-value**
COx_L	-0.105(-0.292—0.043)			0.386(0.281—0.554)			**3.61e-05**
RAP	0.861(0.618—0.949)			0.807(0.513—0.93)			0.781
% time spent within RAP thresholds	RAP < 0	0 ≤ RAP ≤ 0.4	RAP > 0.4	RAP < 0	0 ≤ RAP ≤ 0.4	RAP > 0.4	
	4.28%(2.0%—10.02%)	9.42%(4.83%—16.2%)	84.92%(71.34%—93.17%)	6.25%(3.27%—12.44%)	12.4%(7.13%—20.19%)	80.05%(65.99%—89.25%)	
**COx_R Thresholds**							
**Parameter**	**COx_R < 0**			**COx_R ≥ 0**			**p-value**
COx_R	-0.212(-0.372—-0.098)			0.228(0.107—0.409)			**1.62e-22**
RAP	0.865(0.624—0.951)			0.818(0.536—0.932)			0.157
% time spent within RAP thresholds	RAP < 0	0 ≤ RAP ≤ 0.4	RAP > 0.4	RAP < 0	0 ≤ RAP ≤ 0.4	RAP > 0.4	
	4.36%(1.98%—9.71%)	8.97%(4.33%—18.53%)	86.04%(72.04%—93.49%)	5.31%(2.75%—11.34%)	10.9%(5.96%—20.53%)	83.33%(67.38%—90.38%)	
**Parameter**	**COx_R < 0.20**			**COx_R ≥ 0.20**			**p-value**
COx_R	-0.1(-0.283—0.046)			0.384(0.279—0.549)			**1.49e-07**
RAP	0.855(0.607—0.946)			0.805(0.5—0.929)			0.245
% time spent within RAP thresholds	RAP < 0	0 ≤ RAP ≤ 0.4	RAP > 0.4	RAP < 0	0 ≤ RAP ≤ 0.4	RAP > 0.4	
	4.06%(2.1%—10.23%)	8.7%(4.87%—18.39%)	86.63%(72.87%—93.26%)	6.61%(3.33%—12.39%)	11.68%(6.79%—21.33%)	78.93%(64.53%—88.56%)	
**COx-a_L Thresholds**							
**Parameter**	**COx-a_L < 0**			**COx-a_L ≥ 0**			**p-value**

COx-a_L	-0.197(-0.356—-0.089)			0.247(0.118—0.424)			**1.10e-09**
RAP	0.855(0.607—0.947)			0.849(0.579—0.946)			0.087
% time spent within RAP thresholds	RAP < 0	0 ≤ RAP ≤ 0.4	RAP > 0.4	RAP < 0	0 ≤ RAP ≤ 0.4	RAP > 0.4	
	4.16%(1.87%—10.0%)	10.32%(4.53%—19.37%)	84.24%(73.27%—93.48%)	4.87%(2.29%—10.25%)	9.85%(5.03%—18.6%)	82.96%(70.33%—92.46%)	
**Parameter**	**COx-a_L < 0.20**			**COx-a_L ≥ 0.20**			**p-value**
COx-a_L	-0.073(-0.252—0.064)			0.386(0.283—0.545)			**3.62e-13**
RAP	0.858(0.612—0.948)			0.838(0.548—0.944			0.260
% time spent within RAP thresholds	RAP < 0	0 ≤ RAP ≤ 0.4	RAP > 0.4	RAP < 0	0 ≤ RAP ≤ 0.4	RAP > 0.4	
	4.11%(1.71%—9.76%)	10.27%(4.83%—17.31%)	85.05%(70.15%—93.78%)	6.42%(3.03%—13.21%)	10.83%(6.78%—20.08%)	82.27%(65.16%—90.21%)	
**COx-a_R Thresholds**							
**Parameter**	**COx-a_R < 0**			**COx-a_R ≥ 0**			**p-value**
COx-a_R	-0.19(-0.342—-0.086)			0.238(0.114—0.413)			**1.08e-15**
RAP	0.848(0.597—0.945)			0.843(0.566—0.944)			0.296
% time spent within RAP thresholds	RAP < 0	0 ≤ RAP ≤ 0.4	RAP > 0.4	RAP < 0	0 ≤ RAP ≤ 0.4	RAP > 0.4	
	4.32%(2.01%—9.96%)	9.45%(4.87%—18.73%)	86.17%(69.57%—92.45%)	4.95%(2.62%—11.85%)	10.1%(5.5%—20.15%)	83.66%(68.85%—91.91%)	
**Parameter**	**COx-a_R < 0.20**			**COx-a_R ≥ 0.20**			**p-value**
COx-a_R	-0.069(-0.242—0.064)			0.382(0.28—0.54)			**3.40e-09**
RAP	0.851(0.601—0.945)			0.833(0.533—0.943)			0.401
% time spent within RAP thresholds	RAP < 0	0 ≤ RAP ≤ 0.4	RAP > 0.4	RAP < 0	0 ≤ RAP ≤ 0.4	RAP > 0.4	
	3.72%(1.87—10.38)	9.14(4.39—18.19)	86.38(71.31—93.11)	6.07(3.33—12.33)	10.6(6.73—21.04)	82.78(66.06—89.92)	
**$PbtO_2$ Thresholds**							
**Parameter**	**$PbtO_2$ < 15**			**$PbtO_2$ ≥ 15**			**p-value**
$PbtO_2$	8.775(5.288—11.99)			27.0(21.26—33.37)			**2.91e-18**
RAP	0.774(0.494—0.926)			0.828(0.57—0.935)			0.939
% time spent within RAP thresholds	RAP < 0	0 ≤ RAP ≤ 0.4	RAP > 0.4	RAP < 0	0 ≤ RAP ≤ 0.4	RAP > 0.4	
	5.15%(2.29%—10.49%)	9.96%(3.18%—21.1%)	82.08%(62.5%—91.63%)	5.78%(2.37%—13.88%)	9.98%(4.87%—17.09%)	83.21%(65.2%—92.13%)	

Parameter	$PbtO_2 < 20$			$PbtO_2 \geq 20$			p-value
$PbtO_2$	12.99(7.732—17.0)			29.0(24.31—35.0)			**7.78e-20**
RAP	0.767(0.485—0.92)			0.84(0.596—0.938)			0.601
% time spent within RAP thresholds	RAP < 0	0 ≤ RAP ≤ 0.4	RAP > 0.4	RAP < 0	0 ≤ RAP ≤ 0.4	RAP > 0.4	
	4.76%(2.37%—12.56%)	11.9%(4.97%—22.7%)	81.07%(63.96%—90.73%)	5.66%(2.27%—12.5%)	10.04%(4.64%—18.66%)	84.22%(64.24%—93.21%)	

Supplementary B: Application of Semi-supervised Machine Learning Models for Clustering Parameters - Agglomerative Hierarchical Clustering (AHC)

This supplemantray presents the AHC dendrograms for the entire cohort at lower temporal resolutions, along with the dendrograms derived from the sub-group analyses. A summary table of the cophenetic correlation coefficients for these dendrograms is provided at the end of the supplemantray.

ABP, arterial blood pressure; AMP, pulse amplitude of ICP; COx_L, cerebral oxygenation index of left hemisphere; COx_R, cerebral oxygenation index of right hemisphere; COx-a_L, COx with ABP of left hemisphere; COx-a_R, COx with ABP of the right hemisphere; CPP, cerebral perfusion pressure; ICP, intracranial pressure; MAP, mean arterial pressure; NIRS, near-infrared spectroscopy; PAx, pulse amplitude index; $PbtO_2$, brain tissue oxygenation ; PRx, pressure reactivity index; RAC, a cerebral autoregulation index; RAP, index of cerebral compensatory reserve; rSO_2_L, regional cerebral oxygen saturation of left hemisphere; rSO_2_R, regional cerebral oxygen saturation of the right hemisphere.

Figure S.1 Dendrograms at 5-minute-by-5-minute resolution across whole population

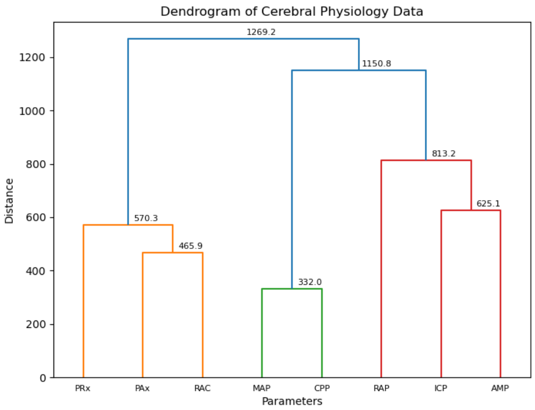

(a) ICP-ABP-derived parameters

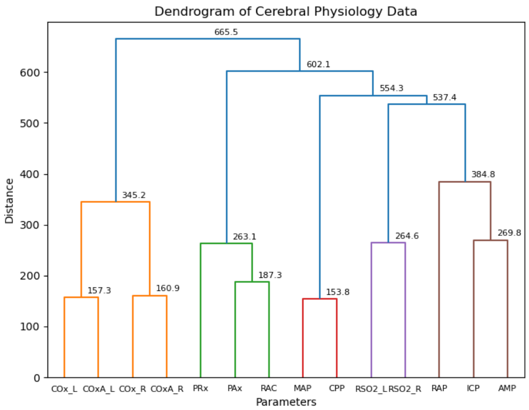

(b) ICP-ABP-derived and NIRS-derived parameters

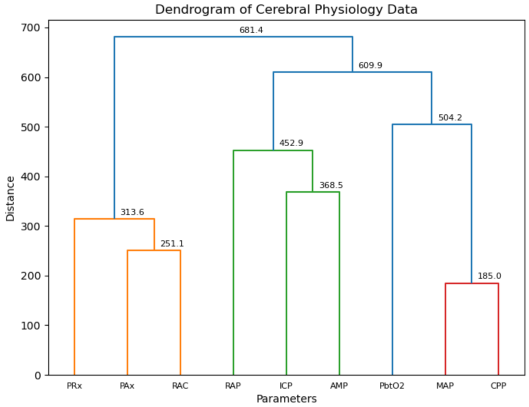

(c) ICP-ABP-derived parameters and $PbtO_2$

Figure S.2 Dendrograms at 10-minute-by-10-minute resolution across whole population

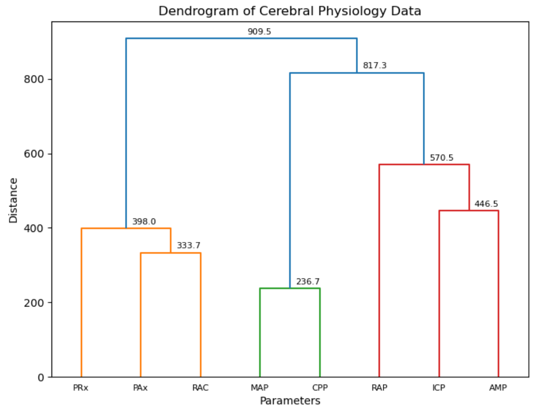

(a) ICP-ABP-derived parameters

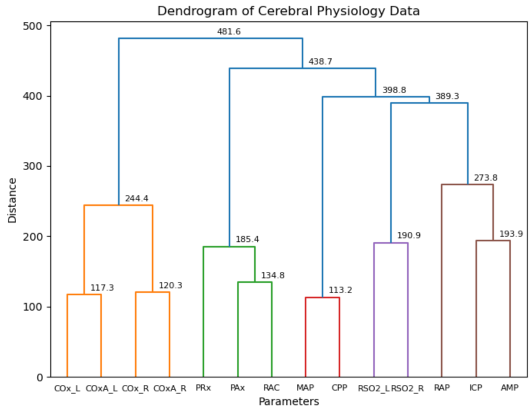

(b) ICP-ABP-derived and NIRS-derived parameters

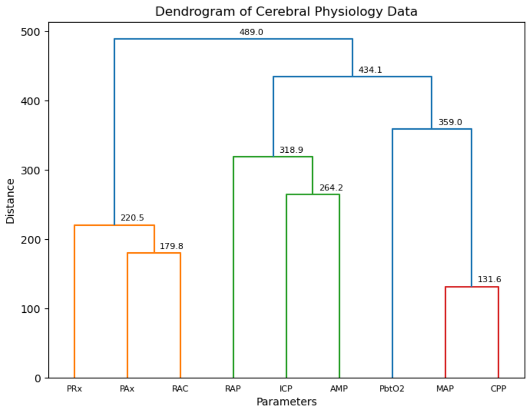

(c) ICP-ABP-derived parameters and $PbtO_2$

Figure S.3 Dendrograms at 30-minute-by-30-minute resolution across whole population

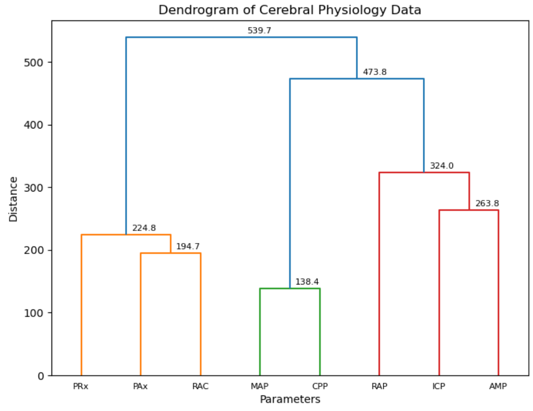

(a) ICP-ABP-derived parameters

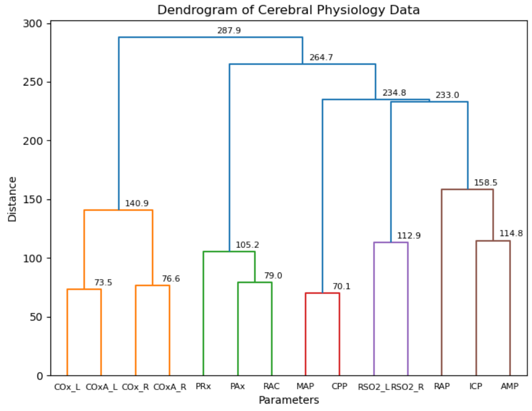

(b) ICP-ABP-derived and NIRS-derived parameters

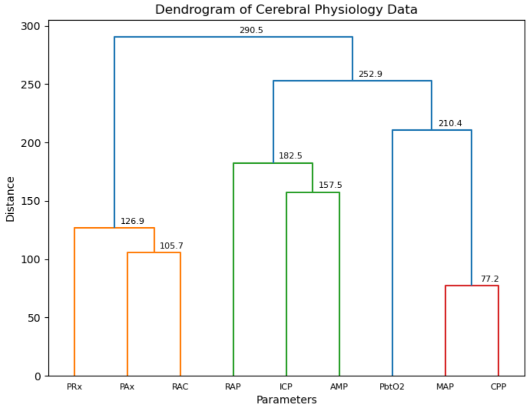

(c) ICP-ABP-derived parameters and $PbtO_2$

Figure S.4 Dendrograms at hour-by-hour resolution across whole population

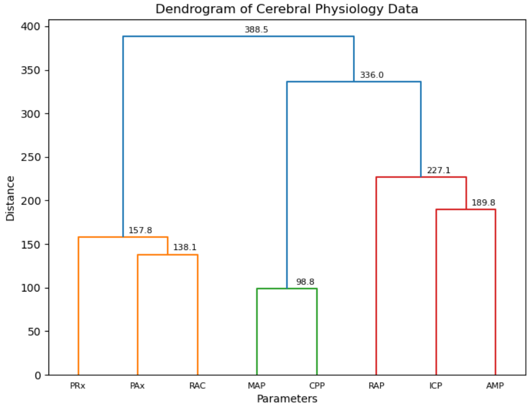

(a) ICP-ABP-derived parameters

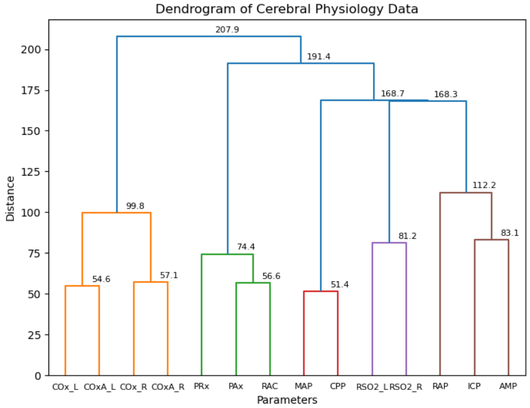

(b) ICP-ABP-derived and NIRS-derived parameters

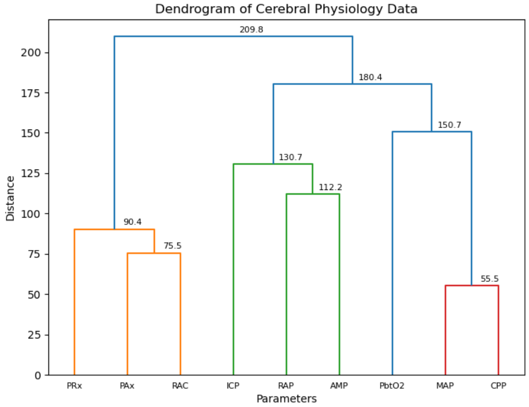

(c) ICP-ABP-derived parameters and $PbtO_2$

Figure S.5 Dendrograms at minute-by-minute resolution across RAP < 0 state

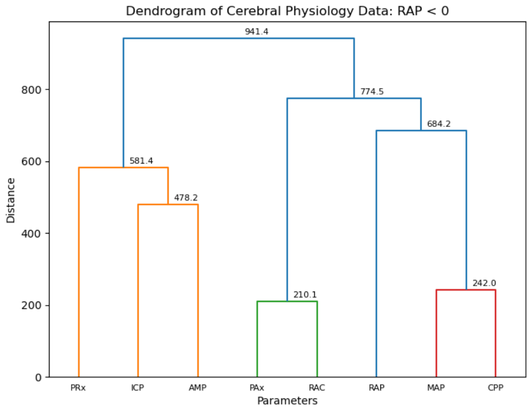

(a) ICP-ABP-derived parameters

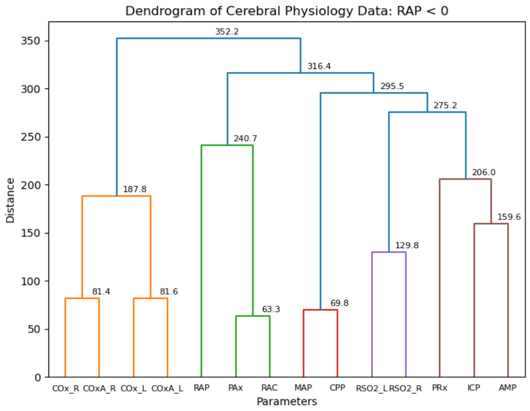

(b) ICP-ABP-derived and NIRS-derived parameters

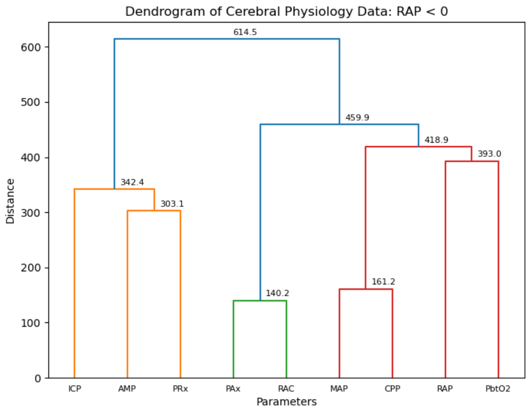

(c) ICP-ABP-derived parameters and $PbtO_2$

Figure S.6 Dendrograms at 5-minute-by-5-minute resolution across RAP < 0 state

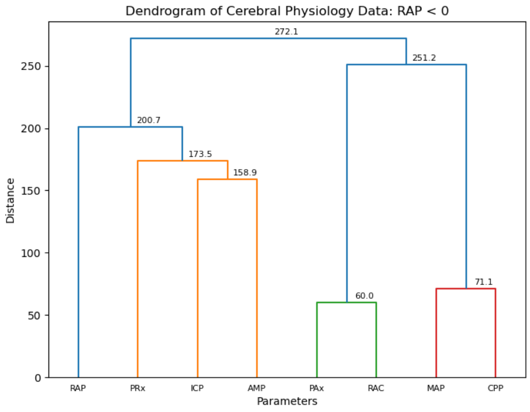

(a) ICP-ABP-derived parameters

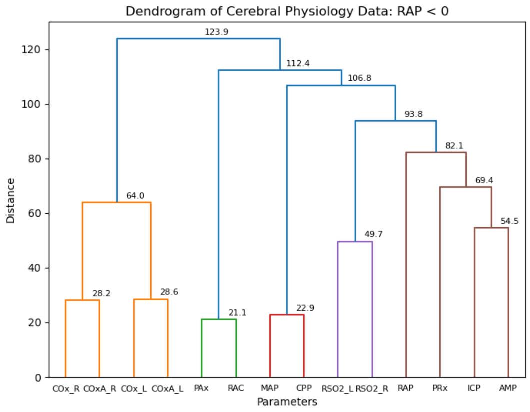

(b) ICP-ABP-derived and NIRS-derived parameters

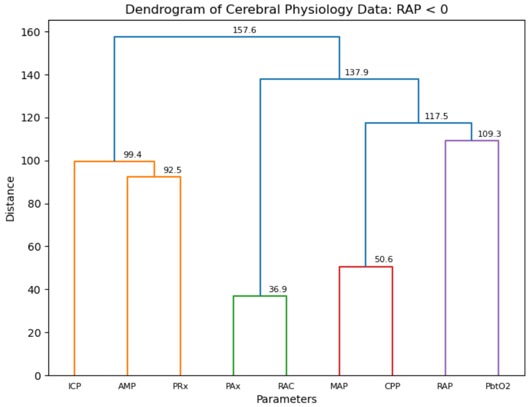

(c) ICP-ABP-derived parameters and $PbtO_2$

Figure S.7 Dendrograms at 10-minute-by-10-minute resolution across RAP < 0 state

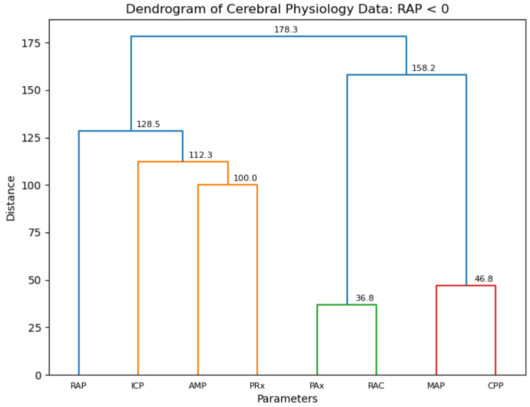

(a) ICP-ABP-derived parameters

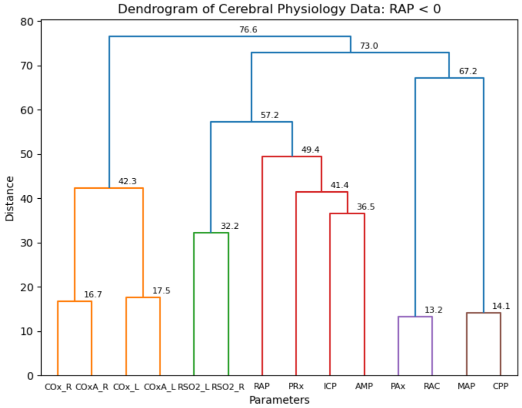

(b) ICP-ABP-derived and NIRS-derived parameters

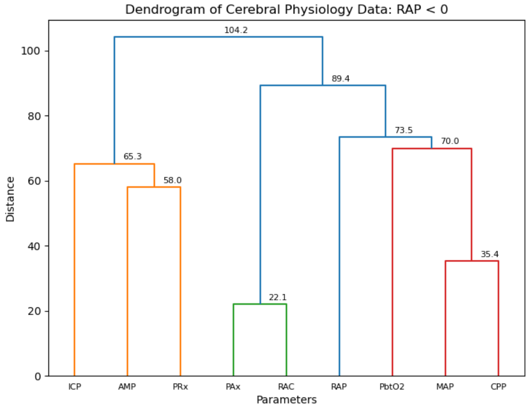

(c) ICP-ABP-derived parameters and $PbtO_2$

Figure S.8 Dendrograms at 30-minute-by-30-minute resolution across RAP < 0 state

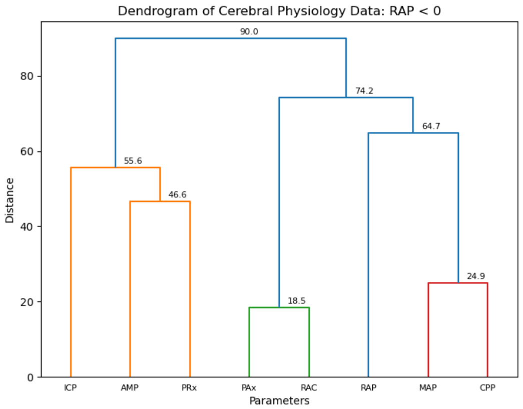

(a) ICP-ABP-derived parameters

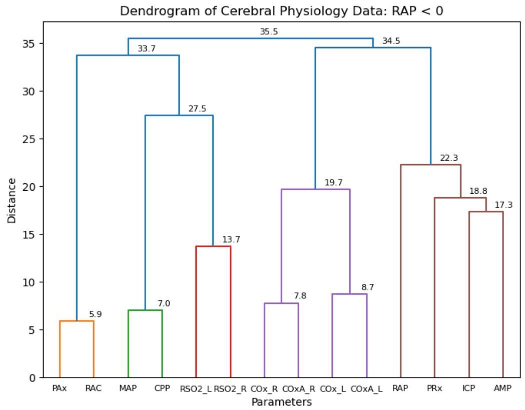

(b) ICP-ABP-derived and NIRS-derived parameters

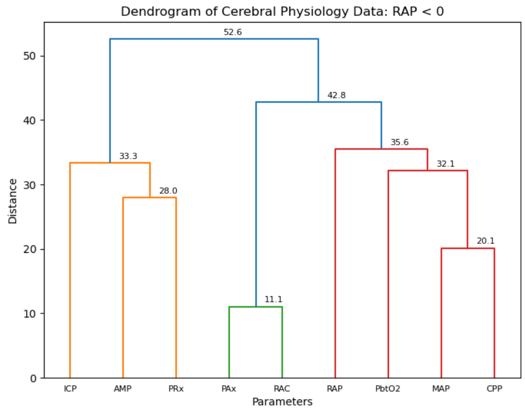

(c) ICP-ABP-derived parameters and $PbtO_2$

Figure S.9 Dendrograms at hour-by-hour resolution across RAP < 0 state

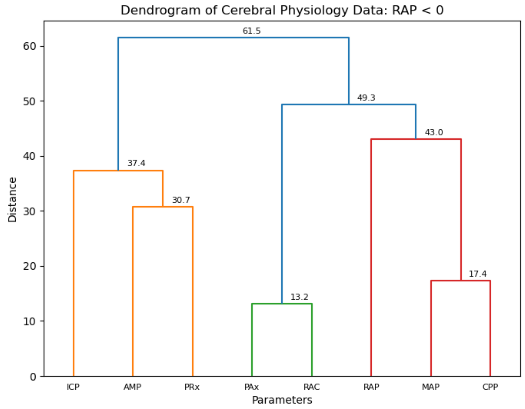

(a) ICP-ABP-derived parameters

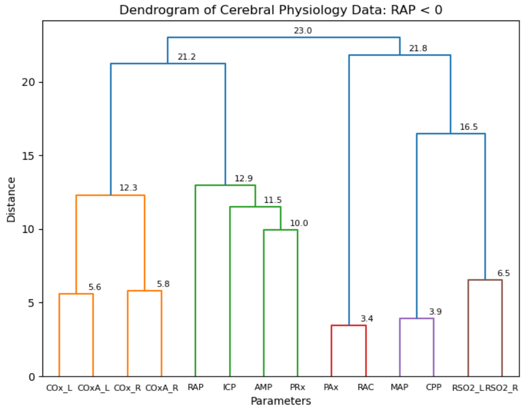

(b) ICP-ABP-derived and NIRS-derived parameters

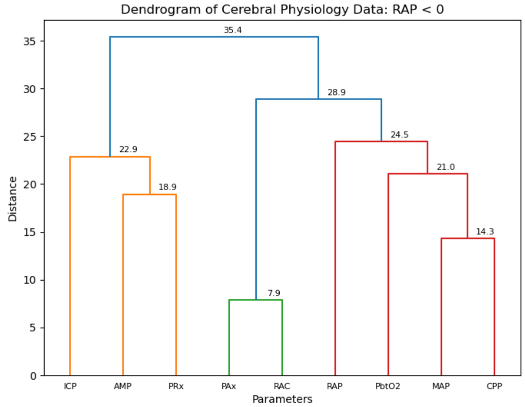

(c) ICP-ABP-derived parameters and $PbtO_2$

Figure S.10 Dendrograms at minute-by-minute resolution across 0 ≤ RAP ≤ 0.4 state

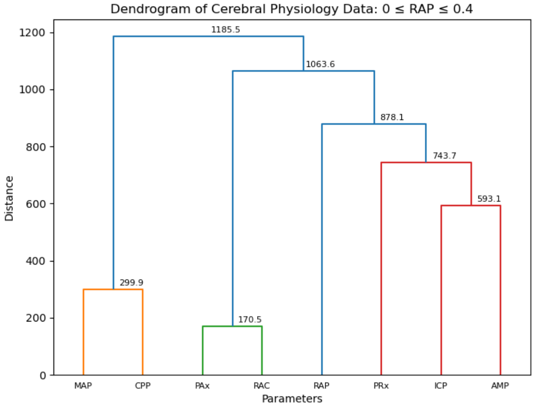

(a) ICP-ABP-derived parameters

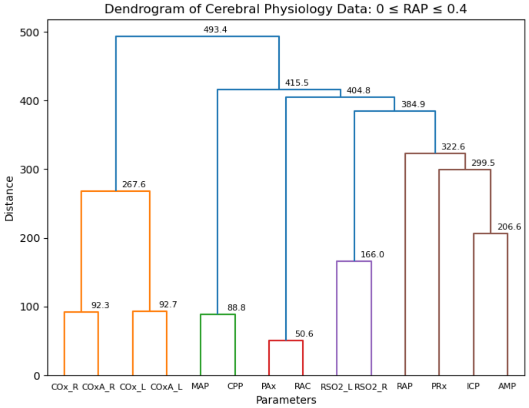

(b) ICP-ABP-derived and NIRS-derived parameters

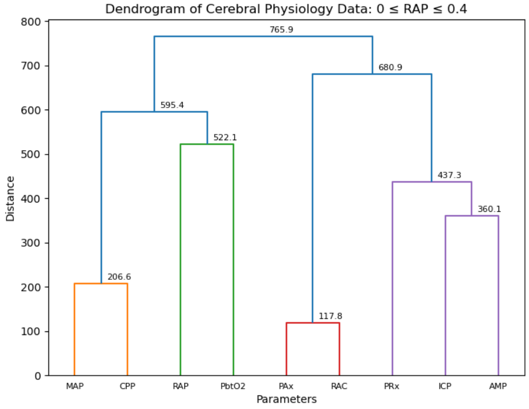

(c) ICP-ABP-derived parameters and $PbtO_2$

Figure S.11 Dendrograms at 5-minute-by-5-minute resolution across 0 ≤ RAP ≤ 0.4 state

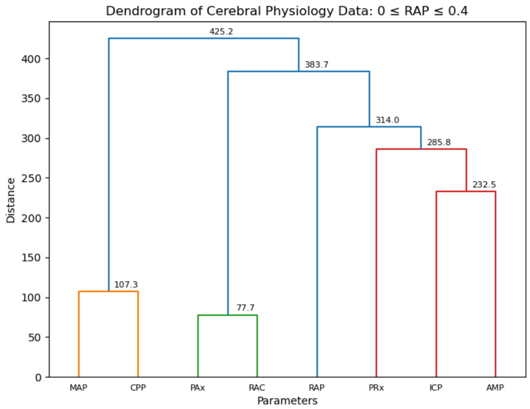

(a) ICP-ABP-derived parameters

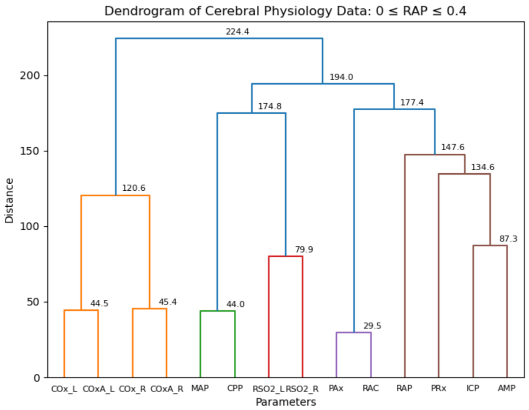

(b) ICP-ABP-derived and NIRS-derived parameters

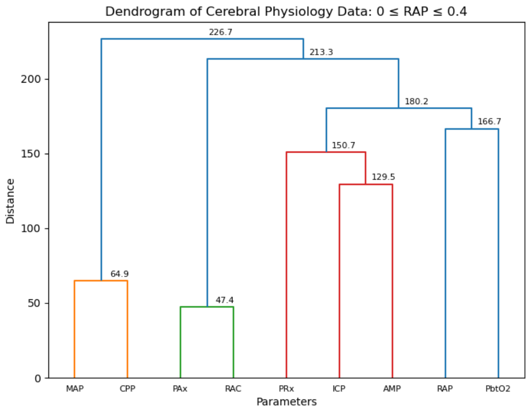

(c) ICP-ABP-derived parameters and $PbtO_2$

Figure S.12 Dendrograms at 10-minute-by-10-minute resolution across 0 ≤ RAP ≤ 0.4 state

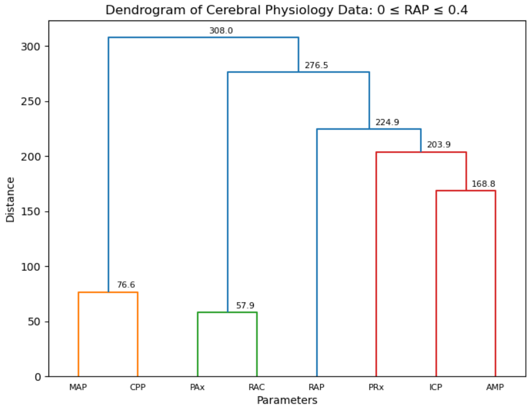

(a) ICP-ABP-derived parameters

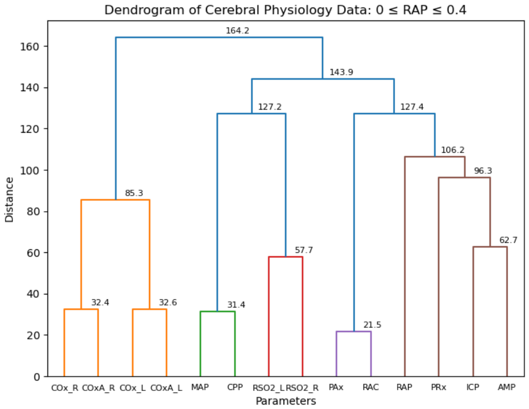

(b) ICP-ABP-derived and NIRS-derived parameters

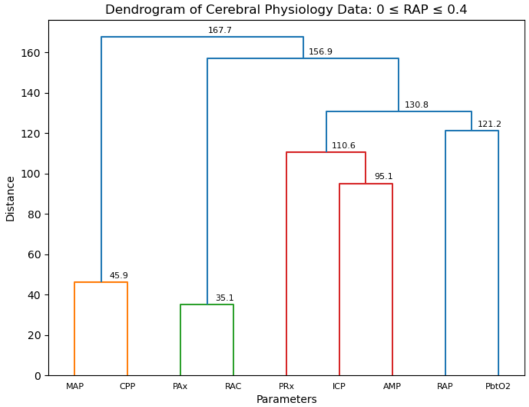

(c) ICP-ABP-derived parameters and $PbtO_2$

Figure S.13 Dendrograms at 30-minute-by-30-minute resolution across $0 \leq RAP \leq 0.4$ state

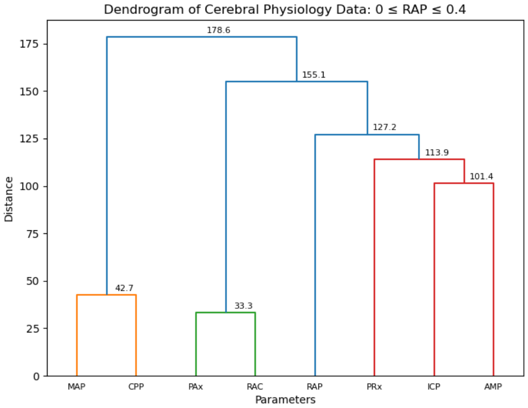

(a) ICP-ABP-derived parameters

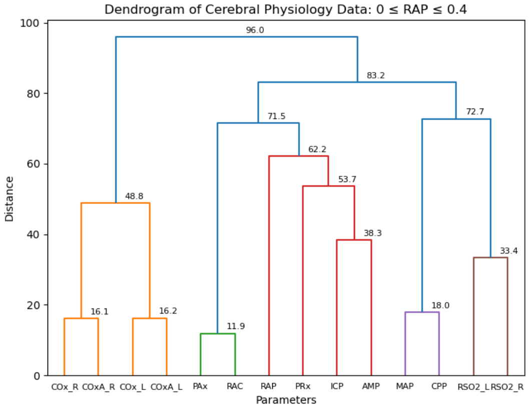

(b) ICP-ABP-derived and NIRS-derived parameters

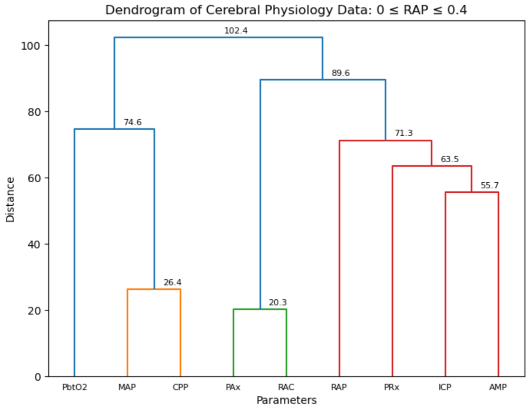

(c) ICP-ABP-derived parameters and $PbtO_2$

Figure S.14 Dendrograms at hour-by-hour resolution across $0 \leq RAP \leq 0.4$ state

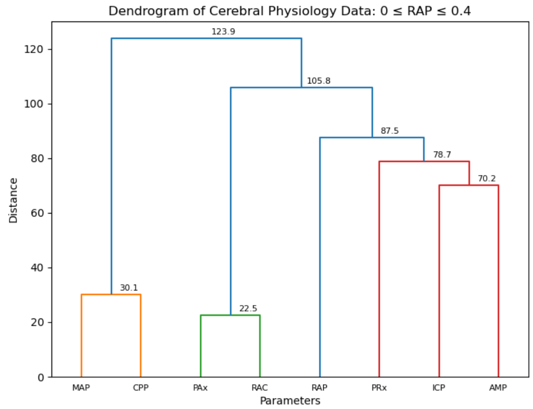

(a) ICP-ABP-derived parameters

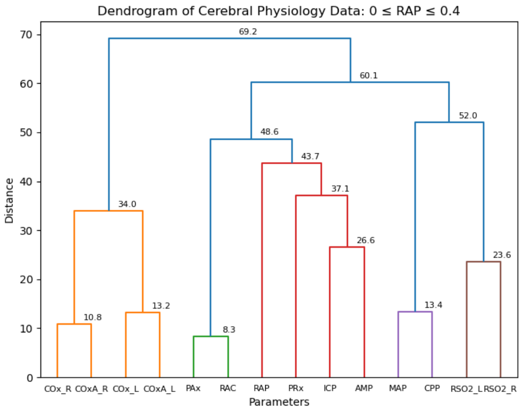

(b) ICP-ABP-derived and NIRS-derived parameters

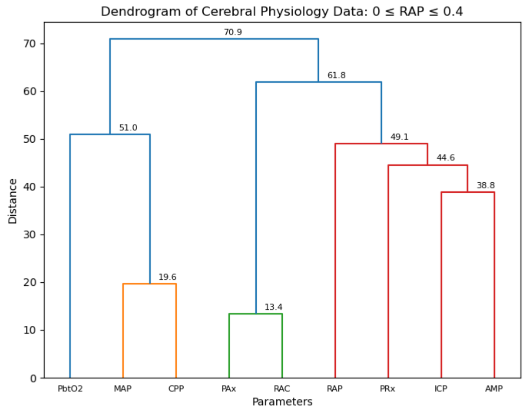

(c) ICP-ABP-derived parameters and $PbtO_2$

Figure S.15 Dendrograms at minute-by-minute resolution across $RAP > 0.4$ state

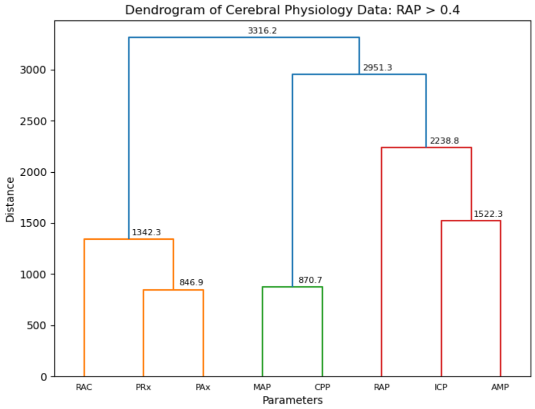

(a) ICP-ABP-derived parameters

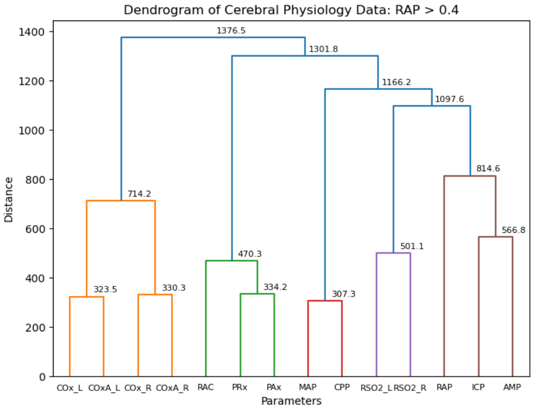

(b) ICP-ABP-derived and NIRS-derived parameters

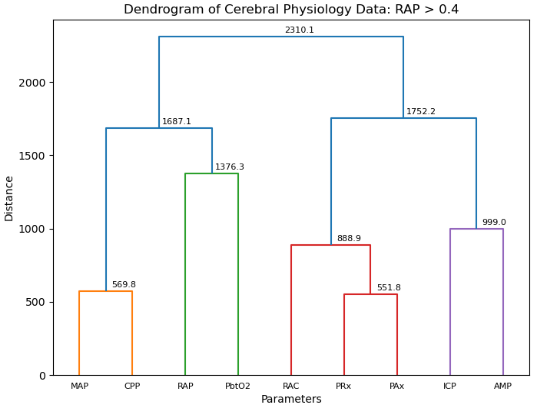

(c) ICP-ABP-derived parameters and $PbtO_2$

Figure S.16 Dendrograms at 5-minute-by-5-minute resolution across RAP > 0.4 state

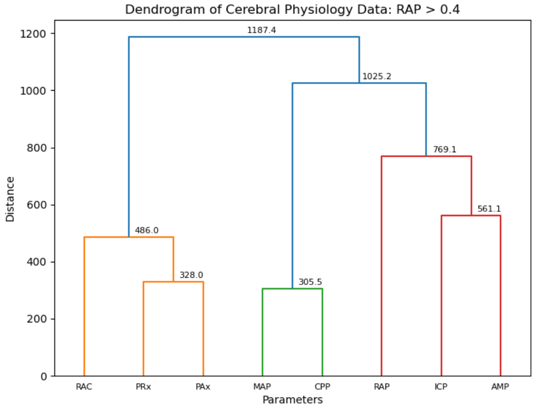

(a) ICP-ABP-derived parameters

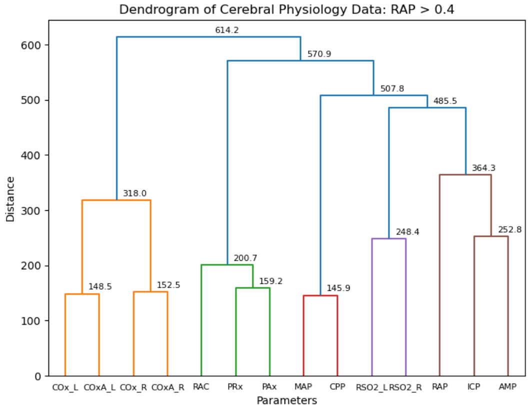

(b) ICP-ABP-derived and NIRS-derived parameters

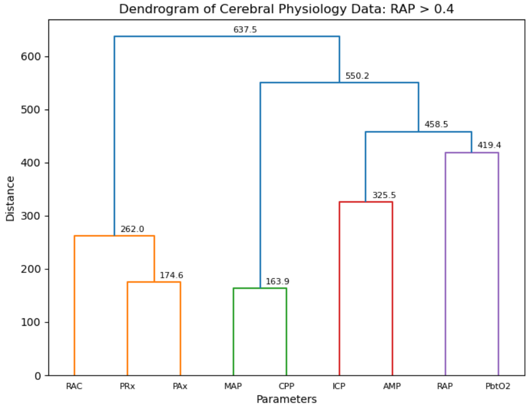

(c) ICP-ABP-derived parameters and $PbtO_2$

Figure S.17 Dendrograms at 10-minute-by-10-minute resolution across RAP > 0.4 state

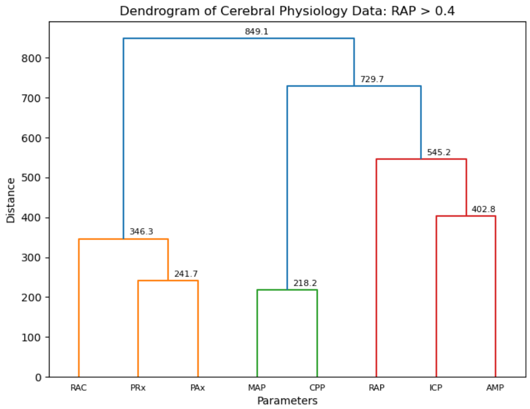

(a) ICP-ABP-derived parameters

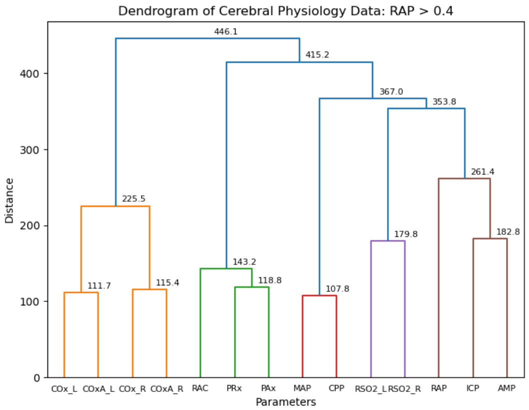

(b) ICP-ABP-derived and NIRS-derived parameters

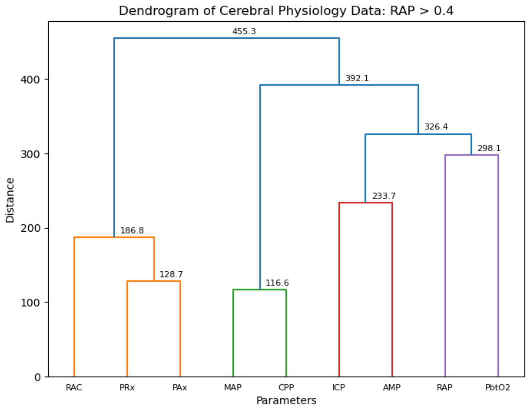

(c) ICP-ABP-derived parameters and $PbtO_2$

Figure S.18 Dendrograms at 30-minute-by-30-minute resolution across RAP > 0.4 state

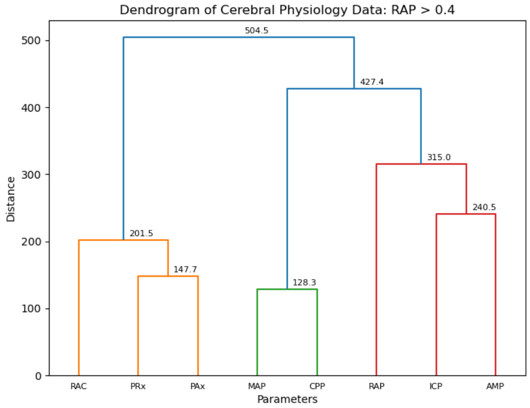

(a) ICP-ABP-derived parameters

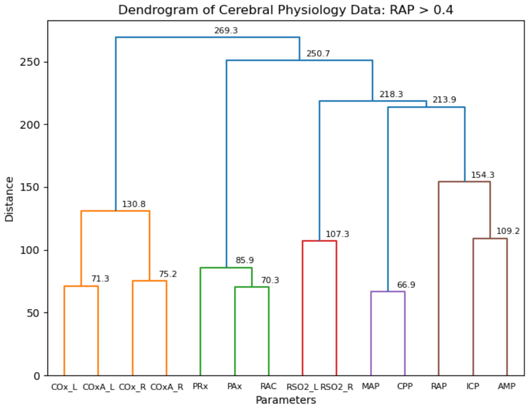

(b) ICP-ABP-derived and NIRS-derived parameters

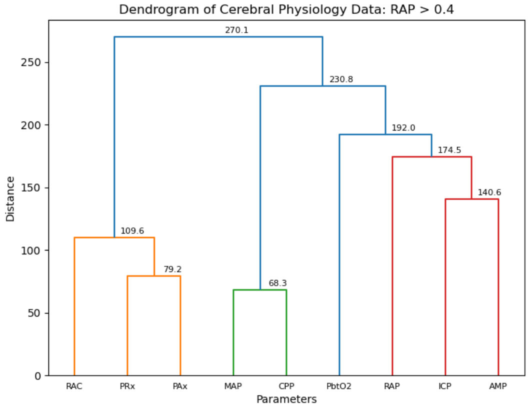

(c) ICP-ABP-derived parameters and $PbtO_2$

Figure S.19 Dendrograms at hour-by-hour resolution across RAP > 0.4 state

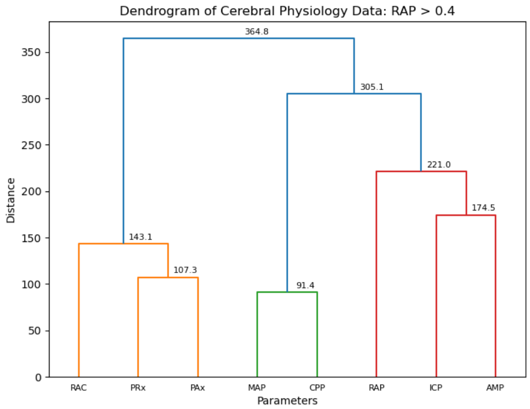

(a) ICP-ABP-derived parameters

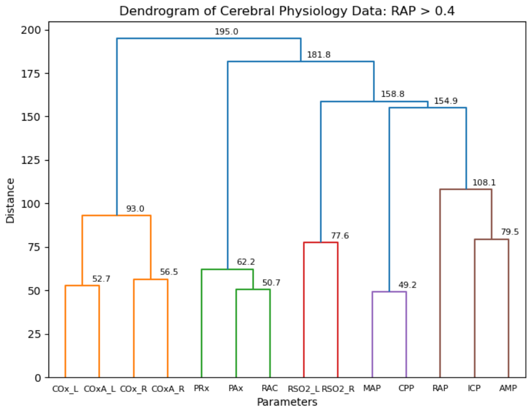

(b) ICP-ABP-derived and NIRS-derived parameters

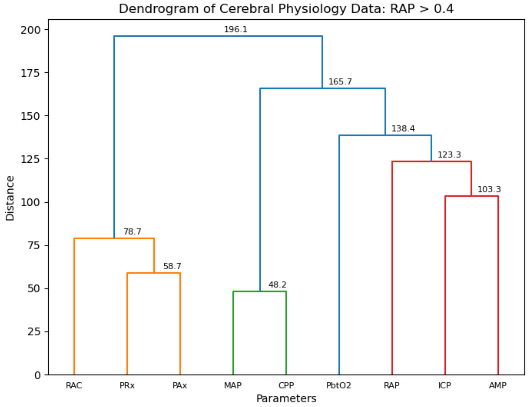

(c) ICP-ABP-derived parameters and $PbtO_2$

Table S.1 The cophenetic correlations for the dendrograms across all the resolutions at sub-group level

**Minute-by-minute**			
**Sub-groups**	**ICP-ABP-derived parameters**	**ICP-ABP-derived and NIRS-derived parameters**	**ICP-ABP-derived parameters and $PbtO_2$**
RAP < 0	0.9	0.86	0.86
0 ≤ RAP ≤ 0.4	0.91	0.86	0.88
RAP > 0.4	0.89	0.9	0.91
**5-minute-by-5-minute**			
**Sub-groups**	**ICP-ABP-derived parameters**	**ICP-ABP-derived and NIRS-derived parameters**	**ICP-ABP-derived parameters and $PbtO_2$**
RAP < 0	0.87	0.86	0.87
0 ≤ RAP ≤ 0.4	0.9	0.88	0.84
RAP > 0.4	0.89	0.89	0.84
**10-minute-by-10-minute**			
**Sub-groups**	**ICP-ABP-derived parameters**	**ICP-ABP-derived and NIRS-derived parameters**	**ICP-ABP-derived parameters and $PbtO_2$**
RAP < 0	0.87	0.87	0.86
0 ≤ RAP ≤ 0.4	0.9	0.88	0.84
RAP > 0.4	0.89	0.89	0.83
**30-minute-by-30-minute**			
**Sub-groups**	**ICP-ABP-derived parameters**	**ICP-ABP-derived and NIRS-derived parameters**	**ICP-ABP-derived parameters and $PbtO_2$**
RAP < 0	0.91	0.89	0.85
0 ≤ RAP ≤ 0.4	0.89	0.88	0.87
RAP > 0.4	0.88	0.88	0.82
**Hour-by-hour**			
**Sub-groups**	**ICP-ABP-derived parameters**	**ICP-ABP-derived and NIRS-derived parameters**	**ICP-ABP-derived parameters and $PbtO_2$**
RAP < 0	0.91	0.86	0.83
0 ≤ RAP ≤ 0.4	0.88	0.88	0.85
RAP > 0.4	0.88	0.88	0.82

Supplementary C: Application of Semi-supervised Machine Learning Models - Principal Component Analysis (PCA)

This supplemantray displays the scree plots of the explained variance ratio and cumulative explained variance ratio across all data resolutions, derived from the principal components following PCA application to the full cohort. Additionally, it includes PCA biplots for the lower resolutions, based on the first two principal components. Biplots for the sub-group analyses are also presented in this supplemantray.

ABP, arterial blood pressure; AMP, pulse amplitude of ICP; COx_L, cerebral oxygenation index of left hemisphere; COx_R, cerebral oxygenation index of right hemisphere; COx-a_L, COx with ABP of left hemisphere; COx-a_R, COx with ABP of the right hemisphere; CPP, cerebral perfusion pressure; ICP, intracranial pressure; MAP, mean arterial pressure; NIRS, near-infrared spectroscopy; PAx, pulse amplitude index; PbtO$_2$*, brain tissue oxygenation ; PRx, pressure reactivity index; RAC, a cerebral autoregulation index; RAP, index of cerebral compensatory reserve; rSO*$_2$*_L, regional cerebral oxygen saturation of left hemisphere; rSO*$_2$*_R, regional cerebral oxygen saturation of the right hemisphere.*

Figure S.1 Scree plots of explained variance ratio at minute-by-minute resolution across whole population

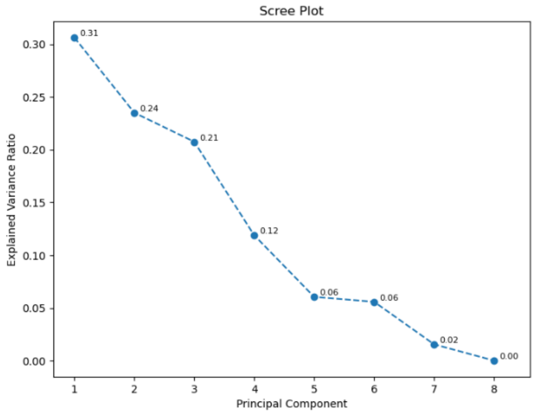

(a) ICP-ABP-derived parameters

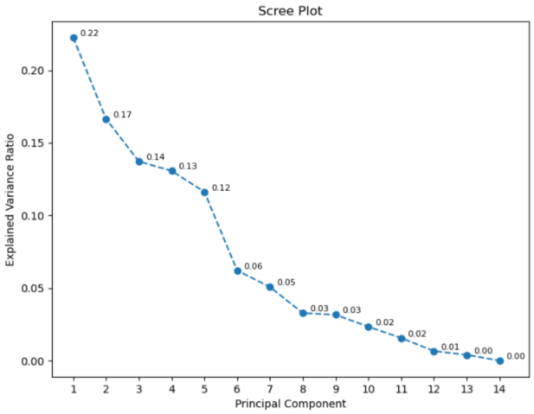

(b) ICP-ABP-derived and NIRS-derived parameters

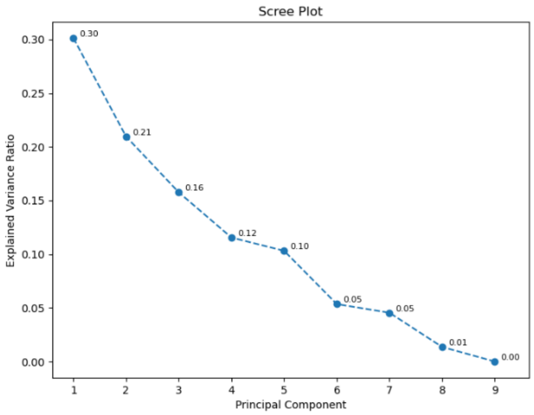

(c) ICP-ABP-derived parameters and PbtO$_2$

Figure S.2 Scree plots of cumulative explained variance at minute-by-minute resolution across whole population

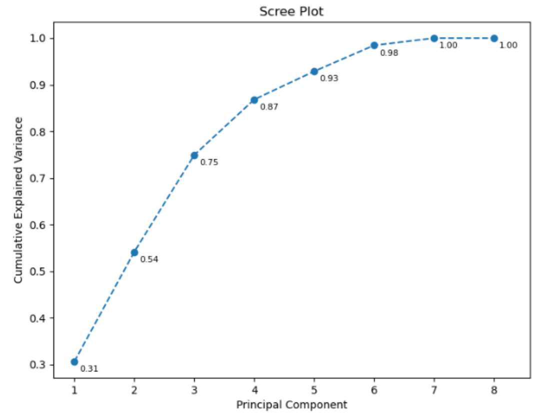

(a) ICP-ABP-derived parameters

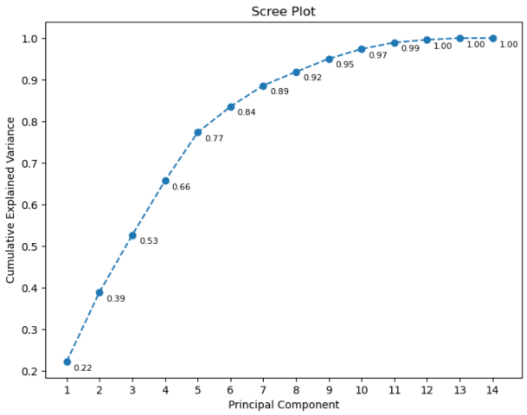

(b) ICP-ABP-derived and NIRS-derived parameters

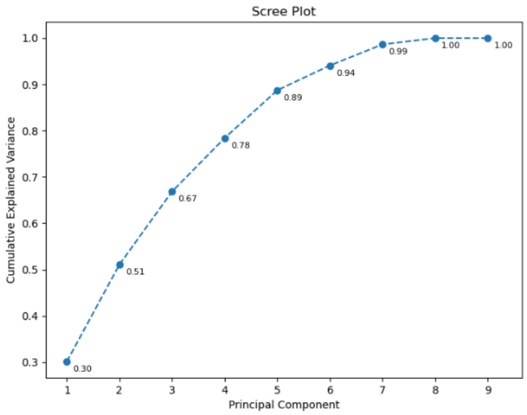

(c) ICP-ABP-derived parameters and $PbtO_2$

Figure S.3 Scree plots of explained variance ratio at 5-minute-by-5-minute resolution across whole population

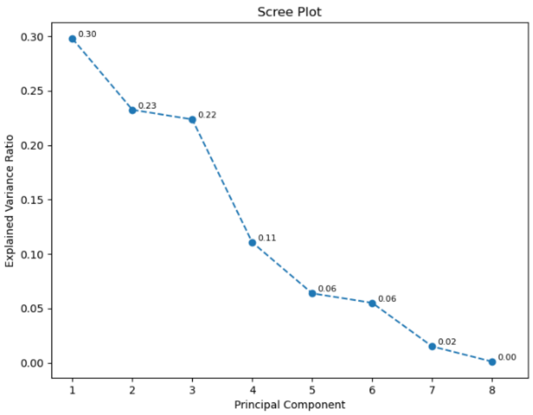

(a) ICP-ABP-derived parameters

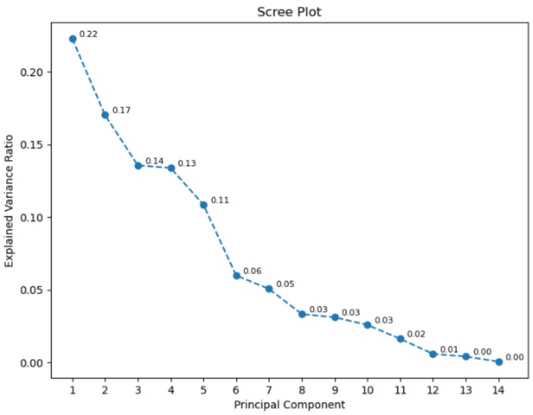

(b) ICP-ABP-derived and NIRS-derived parameters

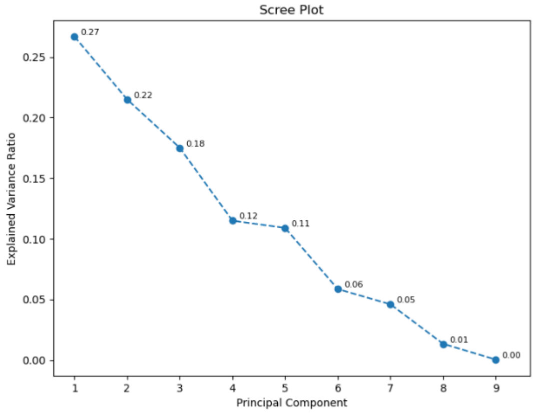

(c) ICP-ABP-derived parameters and $PbtO_2$

Figure S.4 Scree plots of cumulative explained variance at 5-minute-by-5-minute resolution across whole population

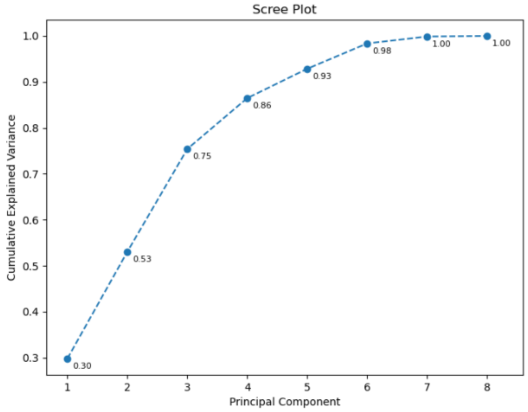

(a) ICP-ABP-derived parameters

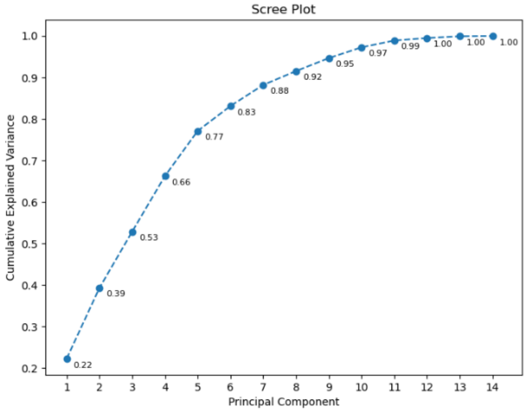

(b) ICP-ABP-derived and NIRS-derived parameters

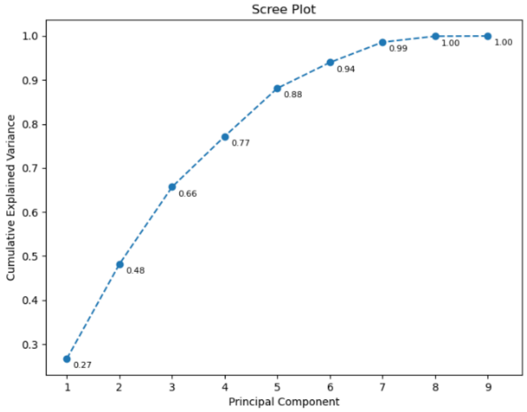

(c) ICP-ABP-derived parameters and $PbtO_2$

Figure S.5 Scree plots of explained variance ratio at 10-minute-by-10-minute resolution across whole population

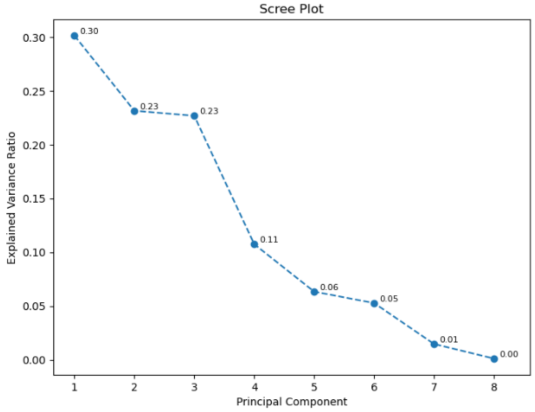

(a) ICP-ABP-derived parameters

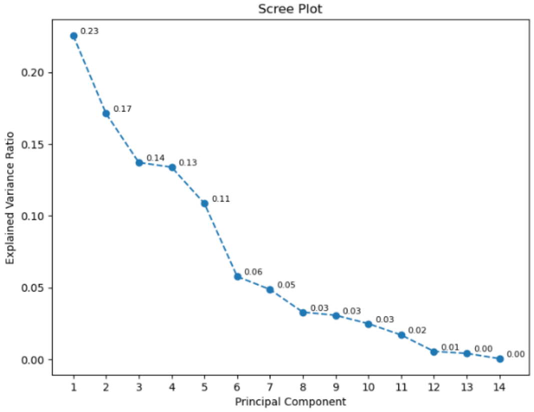

(b) ICP-ABP-derived and NIRS-derived parameters

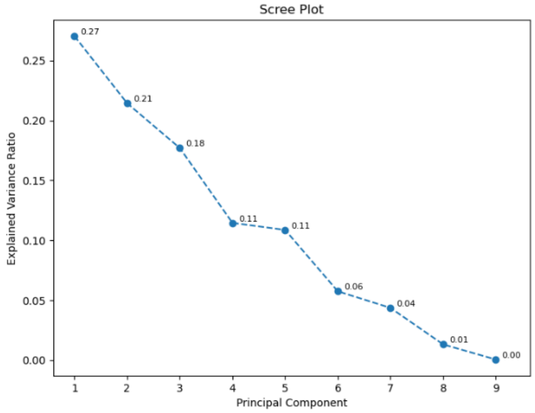

(c) ICP-ABP-derived parameters and $PbtO_2$

Figure S.6 Scree plots of cumulative explained variance at 10-minute-by-10-minute resolution across the whole population

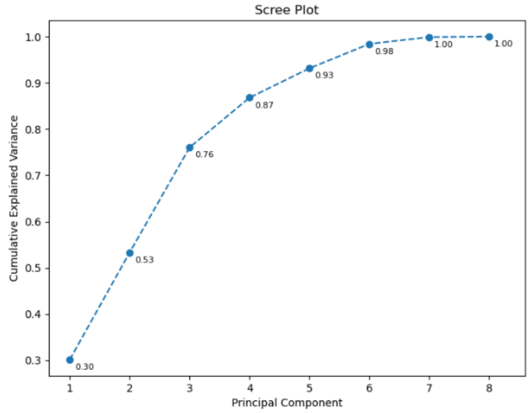

(a) ICP-ABP-derived parameters

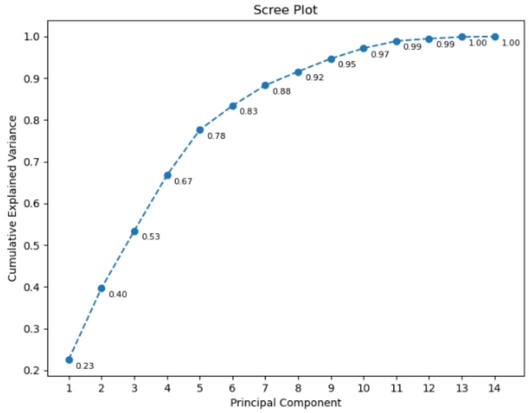

(b) ICP-ABP-derived and NIRS-derived parameters

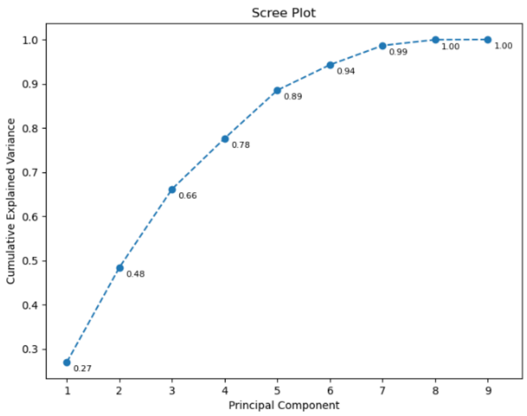

(c) ICP-ABP-derived parameters and $PbtO_2$

Figure S.7 Scree plots of explained variance ratio at 30-minute-by-30-minute resolution across whole population

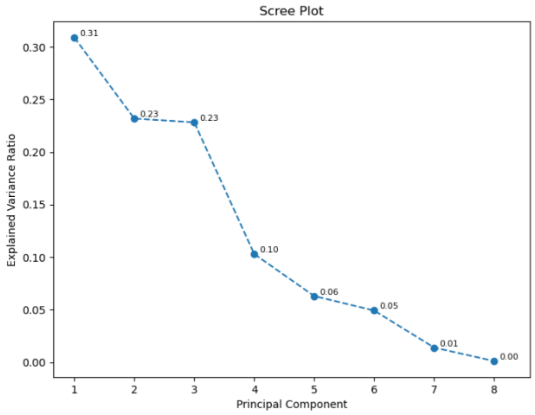

(a) ICP-ABP-derived parameters

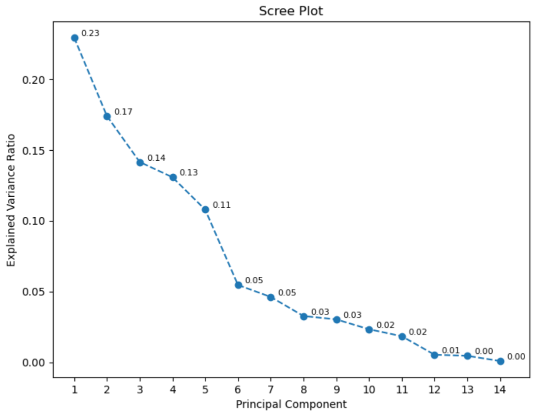

(b) ICP-ABP-derived and NIRS-derived parameters

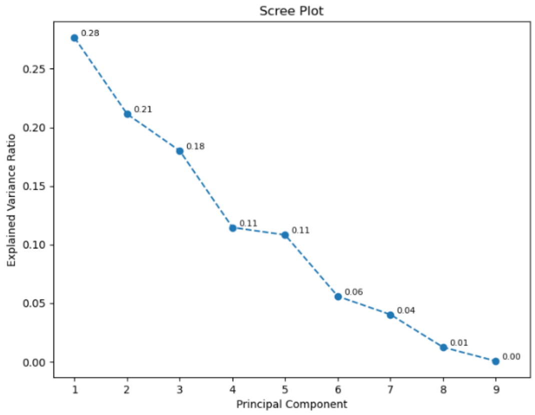

(c) ICP-ABP-derived parameters and $PbtO_2$

Figure S.8 Scree plots of cumulative explained variance at 30-minute-by-30-minute resolution across whole population

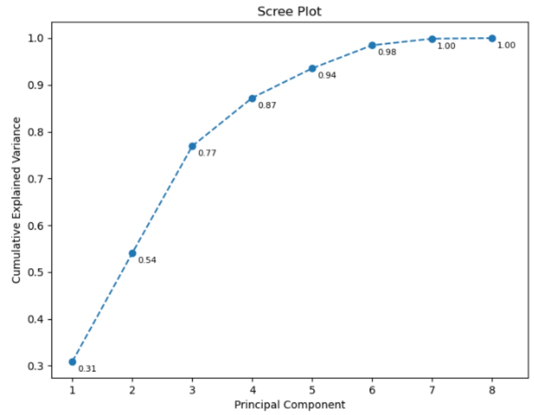

(a) ICP-ABP-derived parameters

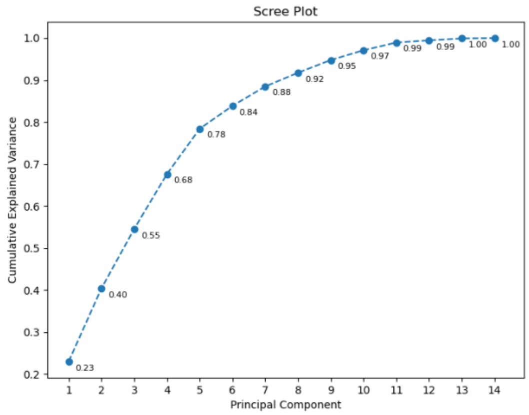

(b) ICP-ABP-derived and NIRS-derived parameters

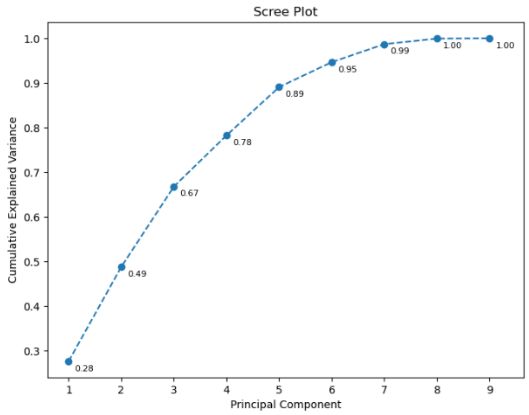

(c) ICP-ABP-derived parameters and $PbtO_2$

Figure S.9 Scree plots of explained variance ratio at hour-by-hour resolution across whole population

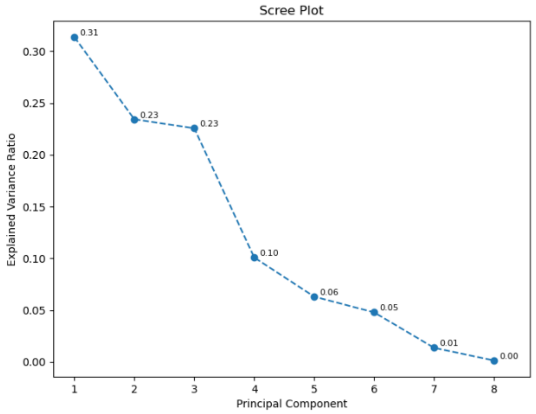

(a) ICP-ABP-derived parameters

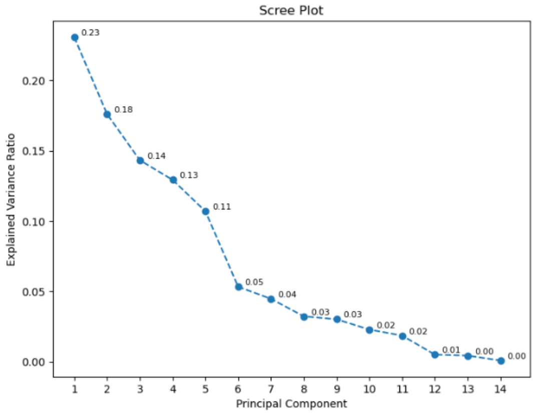

(b) ICP-ABP-derived and NIRS-derived parameters

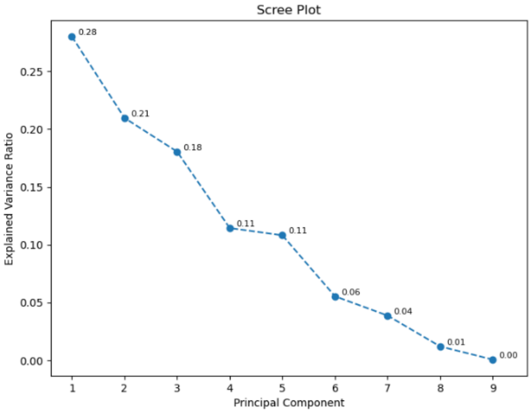

(c) ICP-ABP-derived parameters and $PbtO_2$

Figure S.10 Scree plots of cumulative explained variance at hour-by-hour resolution across whole population

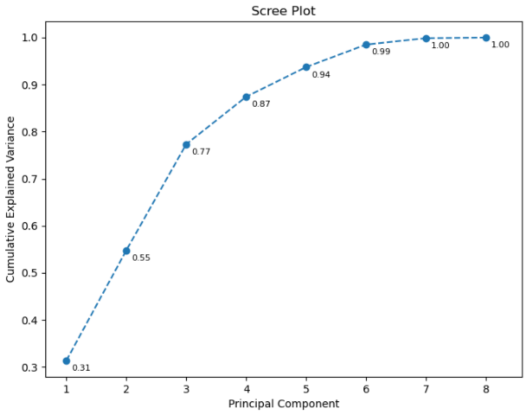

(a) ICP-ABP-derived parameters

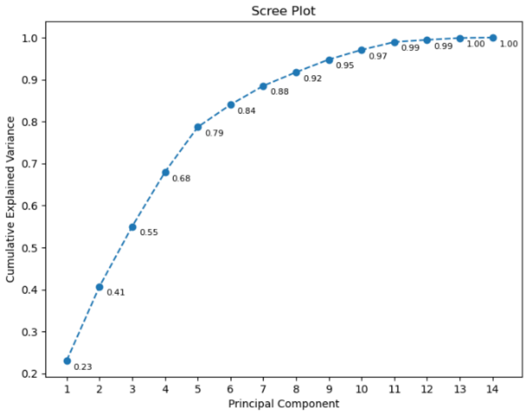

(b) ICP-ABP-derived and NIRS-derived parameters

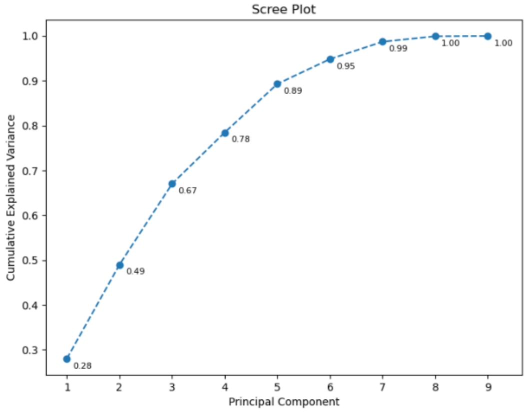

(c) ICP-ABP-derived parameters and $PbtO_2$

Figure S.11 PCA biplots at 5-minute-by-5-minute resolution across whole population

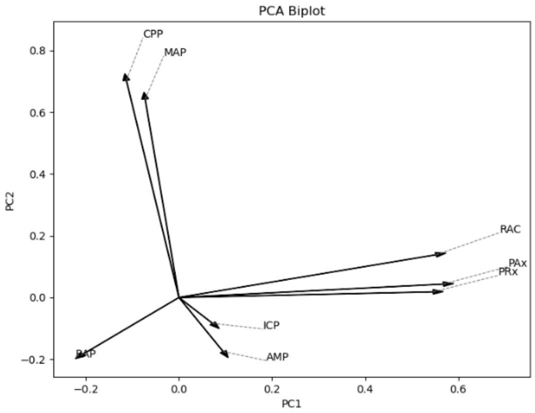

(a) ICP-ABP-derived parameters

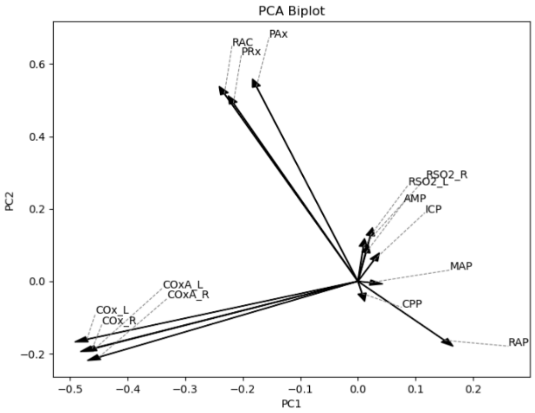

(b) ICP-ABP-derived and NIRS-derived parameters

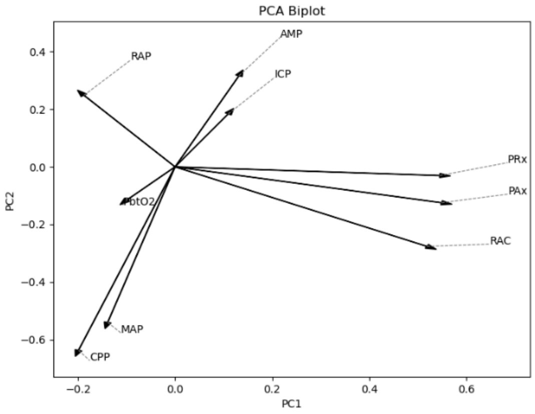

(c) ICP-ABP-derived parameters and $PbtO_2$

Figure S.12 PCA biplots at 10-minute-by-10-minute resolution across whole population

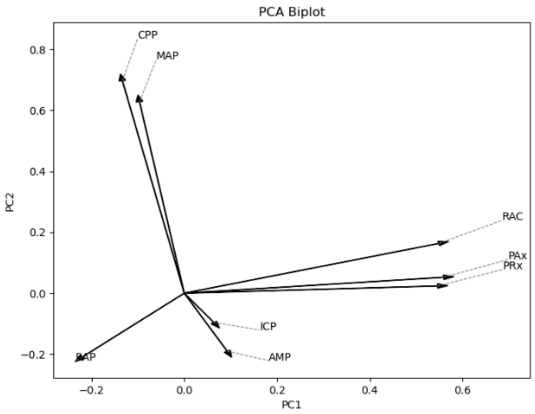

(a) ICP-ABP-derived parameters

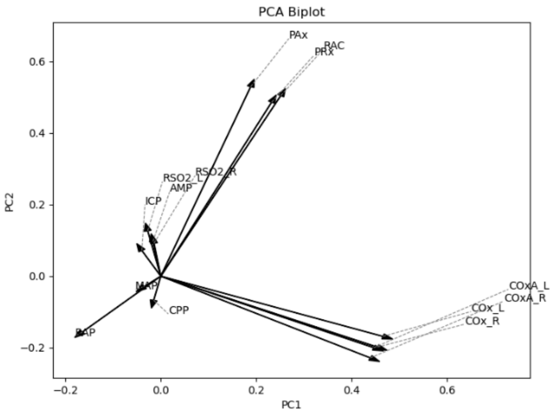

(b) ICP-ABP-derived and NIRS-derived parameters

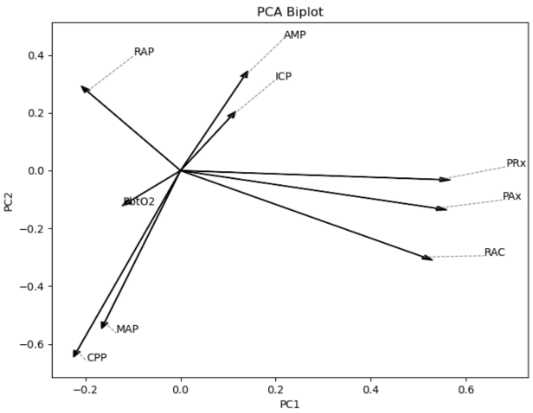

(c) ICP-ABP-derived parameters and $PbtO_2$

Figure S.13 PCA biplots at 30-minute-by-30-minute resolution across whole population

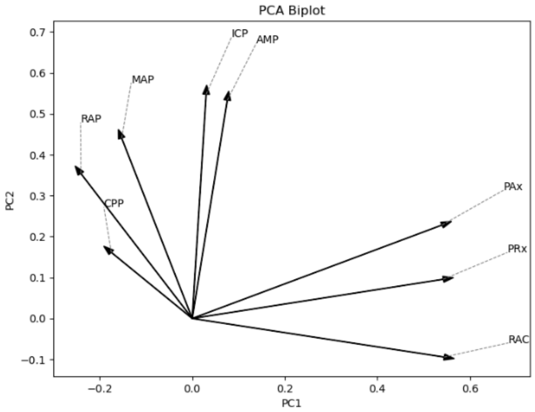

(a) ICP-ABP-derived parameters

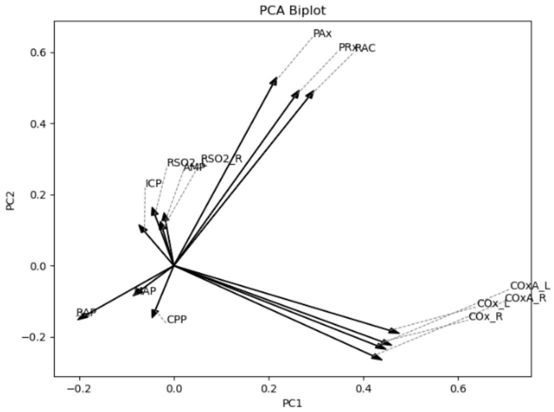

(b) ICP-ABP-derived and NIRS-derived parameters

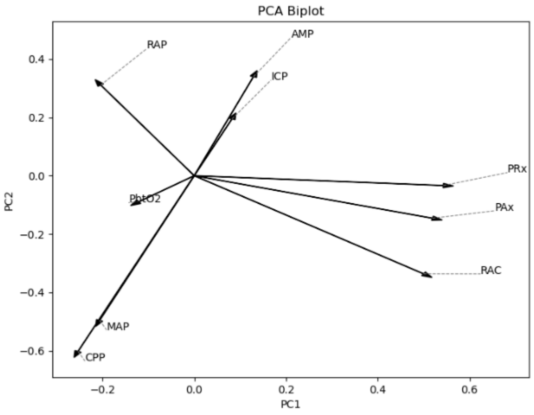

(c) ICP-ABP-derived parameters and $PbtO_2$

Figure S.14 PCA biplots at hour-by-hour resolution across whole population

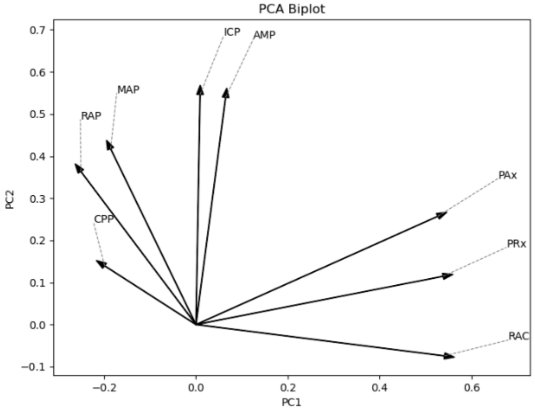

(a) ICP-ABP-derived parameters

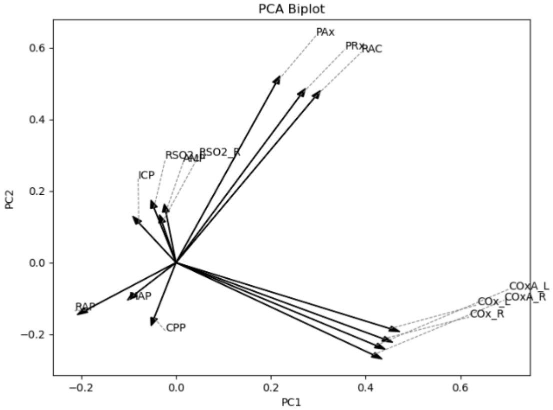

(b) ICP-ABP-derived and NIRS-derived parameters

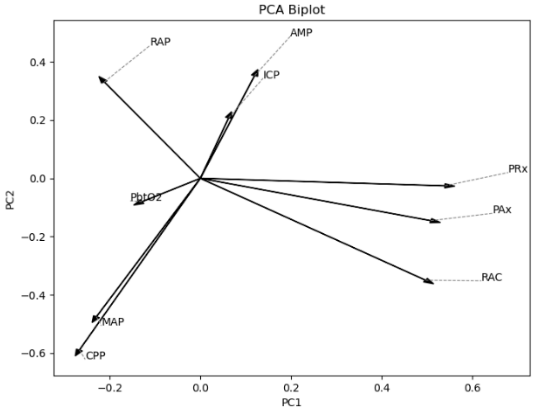

(c) ICP-ABP-derived parameters and $PbtO_2$

Figure S.15 PCA biplots at minute-by-minute resolution across RAP < 0 state

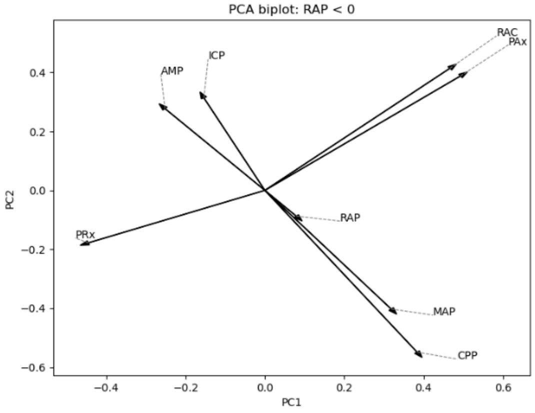

(a) ICP-ABP-derived parameters

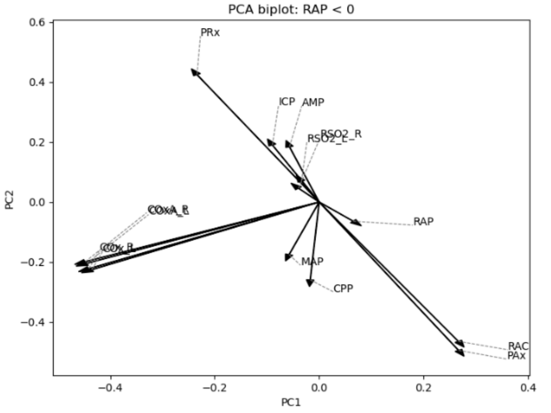

(b) ICP-ABP-derived and NIRS-derived parameters

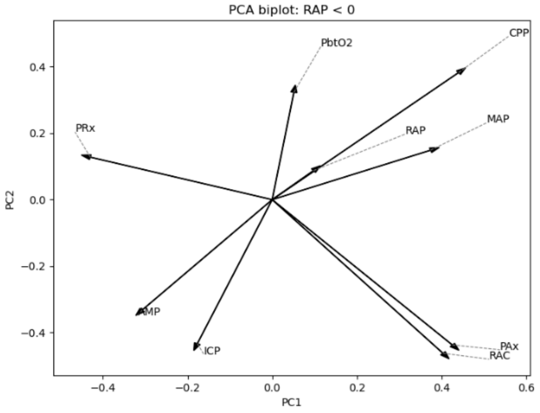

(c) ICP-ABP-derived parameters and $PbtO_2$

Figure S.16 PCA biplots at 5-minute-by-5-minute resolution across RAP < 0 state

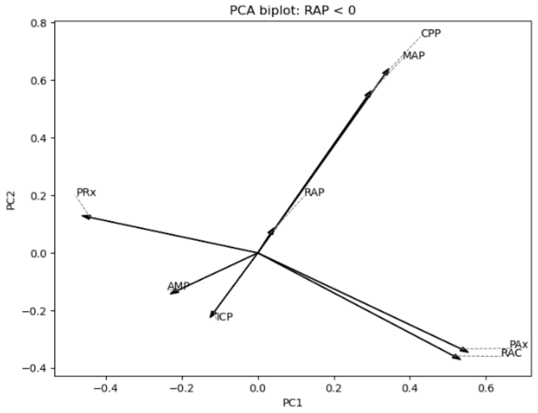

(a) ICP-ABP-derived parameters

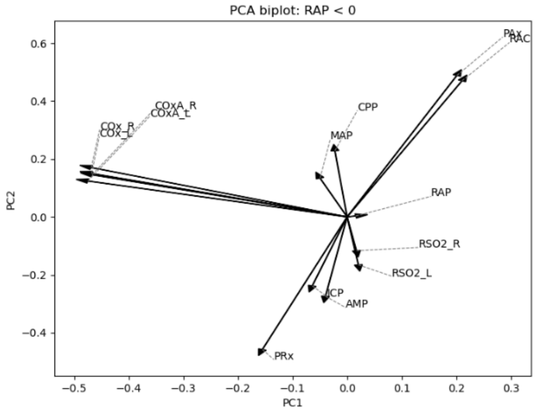

(b) ICP-ABP-derived and NIRS-derived parameters

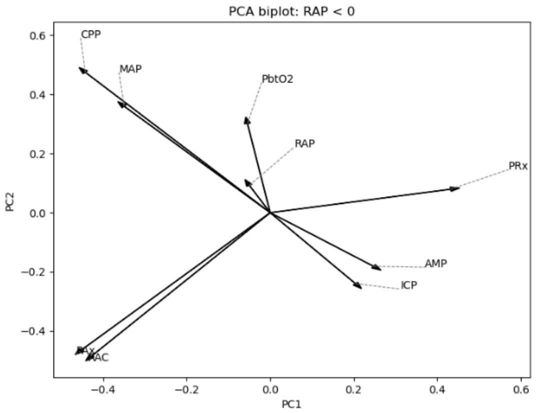

(c) ICP-ABP-derived parameters and $PbtO_2$

Figure S.17 PCA biplots at 10-minute-by-10-minute resolution across RAP < 0 state

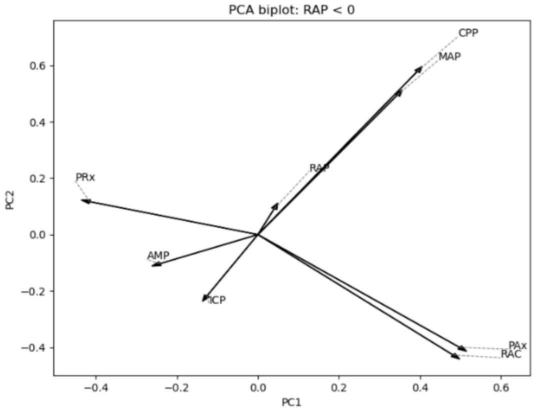

(a) ICP-ABP-derived parameters

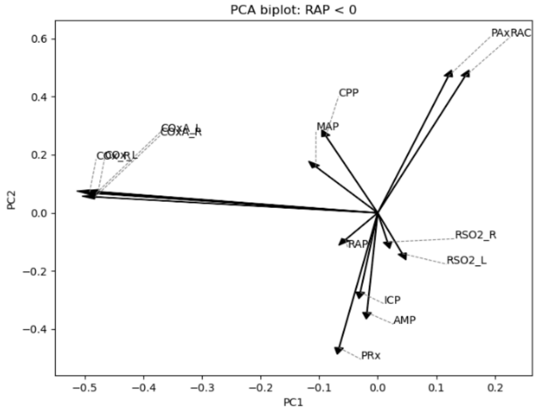

(b) ICP-ABP-derived and NIRS-derived parameters

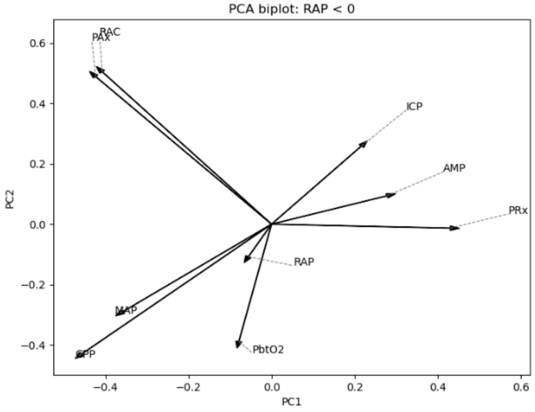

(c) ICP-ABP-derived parameters and $PbtO_2$

Figure S.18 PCA biplots at 30-minute-by-30-minute resolution across RAP < 0 state

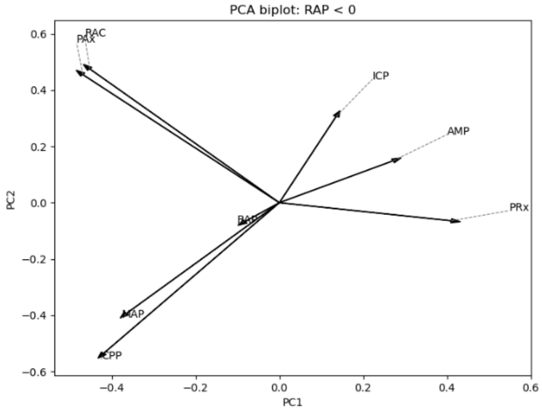

(a) ICP-ABP-derived parameters

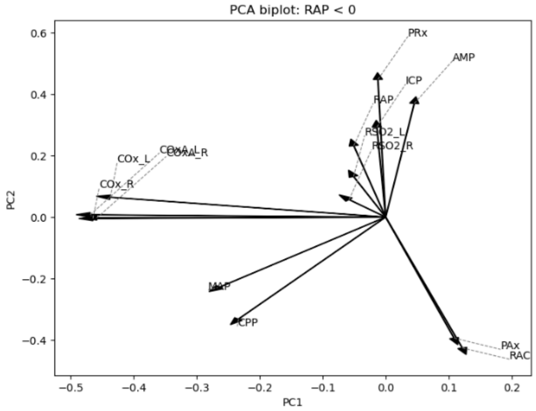

(b) ICP-ABP-derived and NIRS-derived parameters

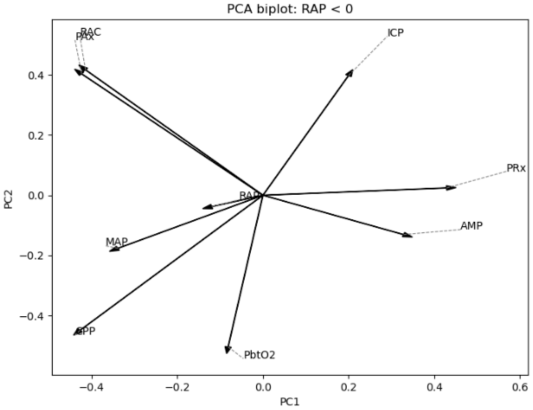

(c) ICP-ABP-derived parameters and $PbtO_2$

Figure S.19 PCA biplots at hour-by-hour resolution across RAP < 0 state

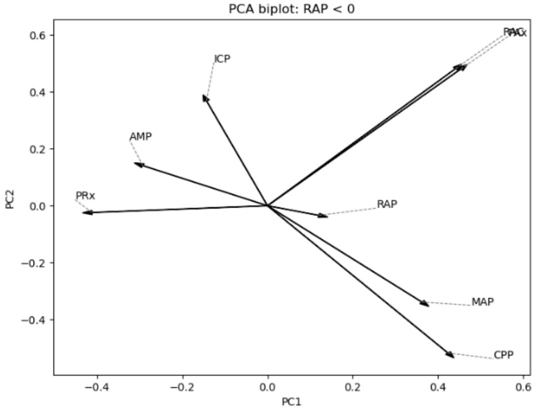

(a) ICP-ABP-derived parameters

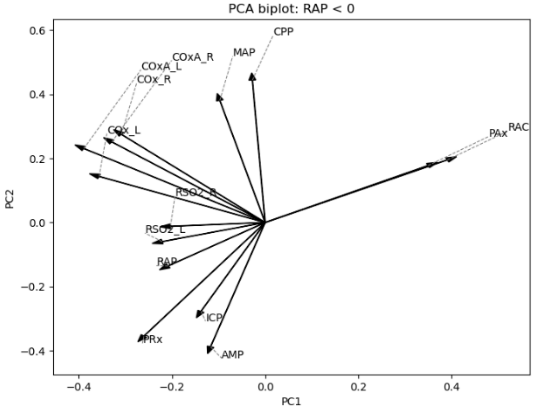

(b) ICP-ABP-derived and NIRS-derived parameters

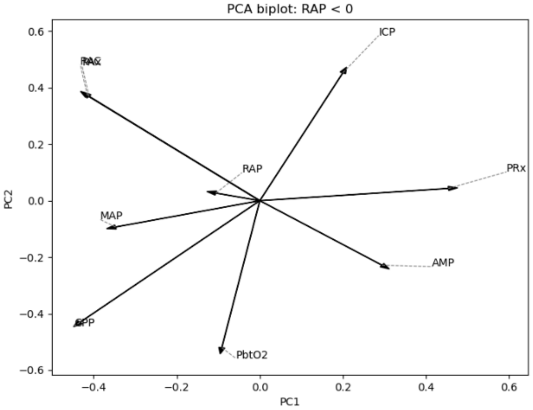

(c) ICP-ABP-derived parameters and $PbtO_2$

Figure S.20 PCA biplots at minute-by-minute resolution across 0 ≤ RAP ≤ 0.4 state

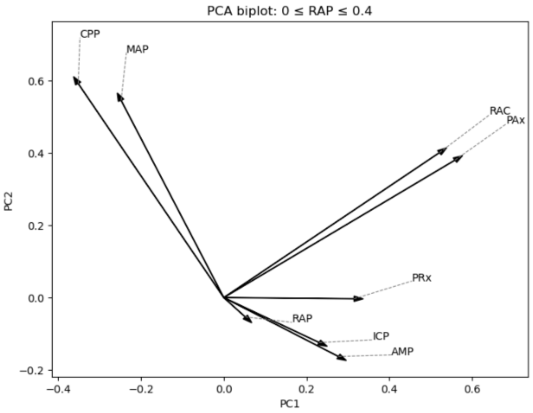

(a) ICP-ABP-derived parameters

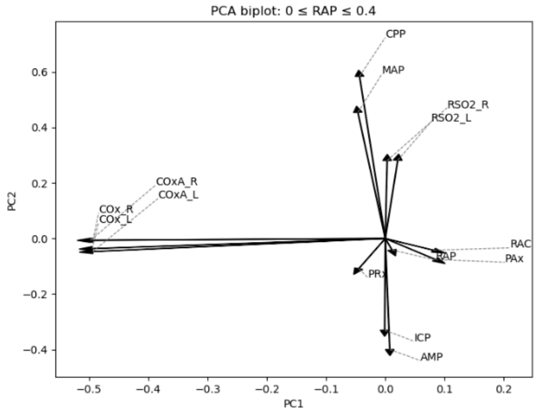

(b) ICP-ABP-derived and NIRS-derived parameters

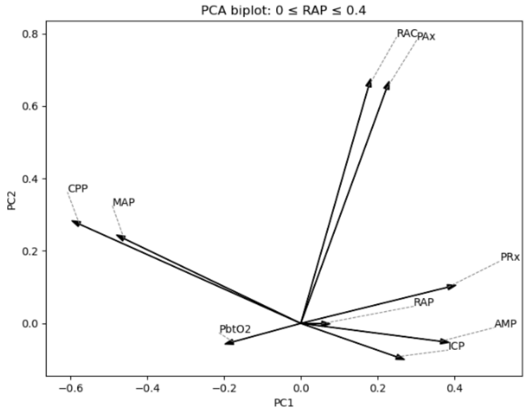

(c) ICP-ABP-derived parameters and $PbtO_2$

Figure S.21 PCA biplots at 5-minute-by-5-minute resolution across 0 ≤ RAP ≤ 0.4 state

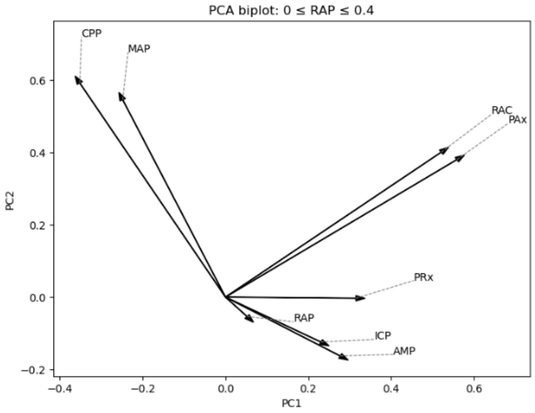

(a) ICP-ABP-derived parameters

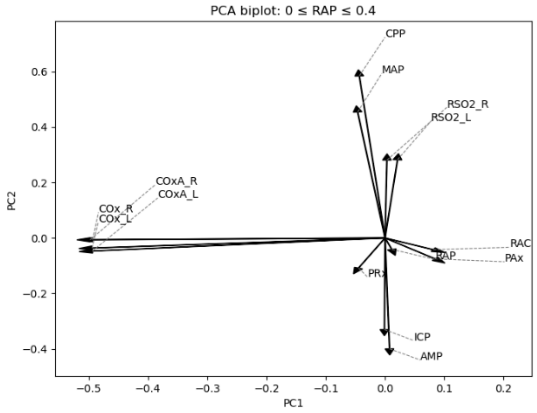

(b) ICP-ABP-derived and NIRS-derived parameters

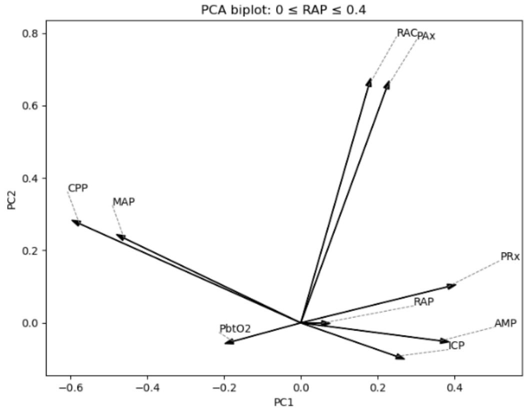

(c) ICP-ABP-derived parameters and $PbtO_2$

Figure S.22 PCA biplots at 10-minute-by-10-minute resolution across 0 ≤ RAP ≤ 0.4 state

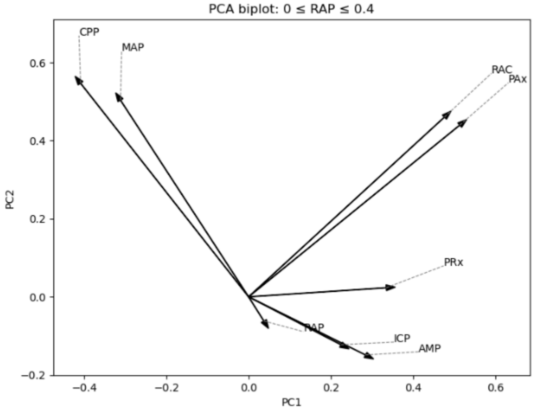

(a) ICP-ABP-derived parameters

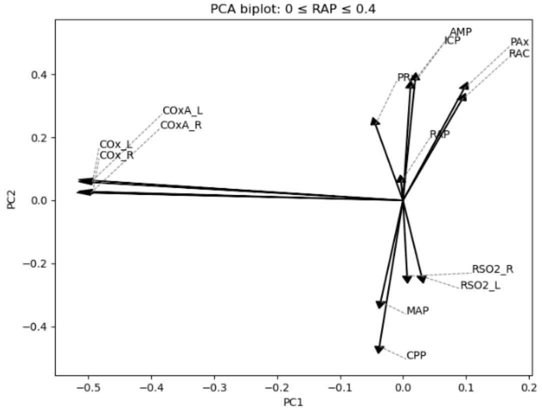

(b) ICP-ABP-derived and NIRS-derived parameters

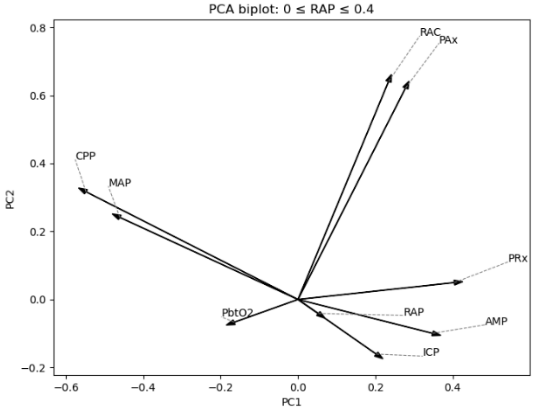

(c) ICP-ABP-derived parameters and $PbtO_2$

Figure S.23 PCA biplots at 30-minute-by-30-minute resolution across 0 ≤ RAP ≤ 0.4 state

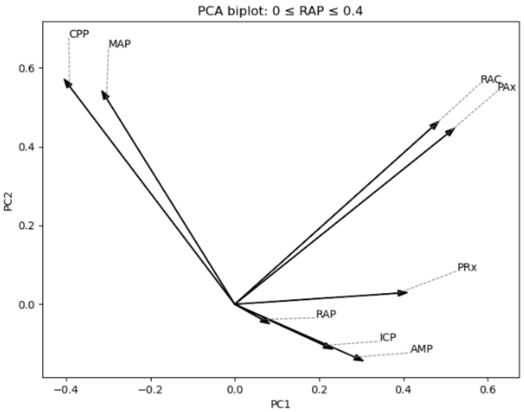

(a) ICP-ABP-derived parameters

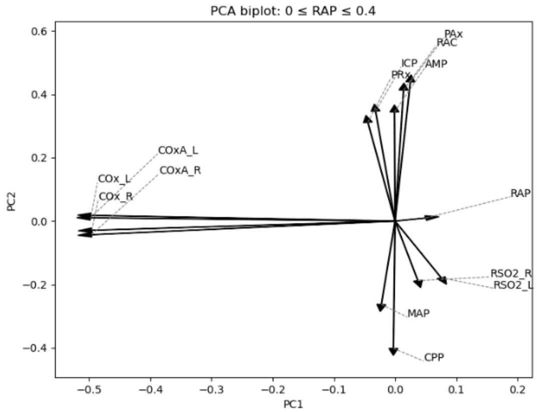

(b) ICP-ABP-derived and NIRS-derived parameters

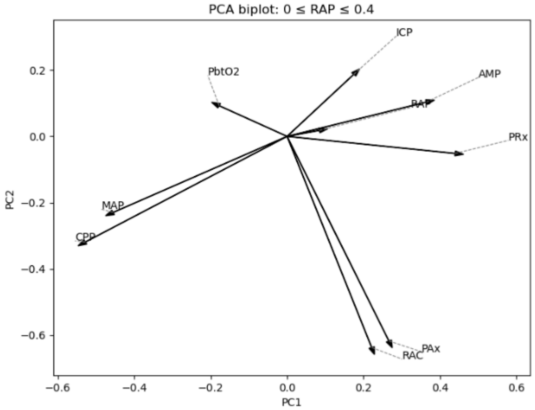

(c) ICP-ABP-derived parameters and $PbtO_2$

Figure S.24 PCA biplots at hour-by-hour resolution across 0 ≤ RAP ≤ 0.4 state

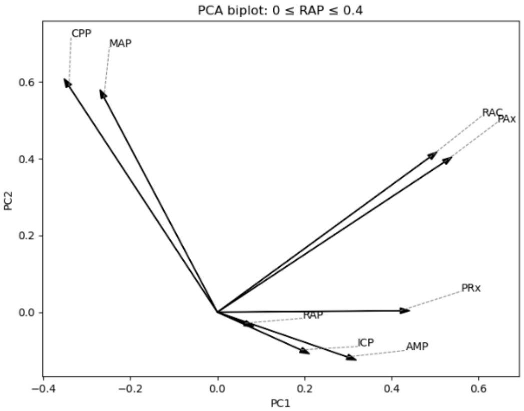

(a) ICP-ABP-derived parameters

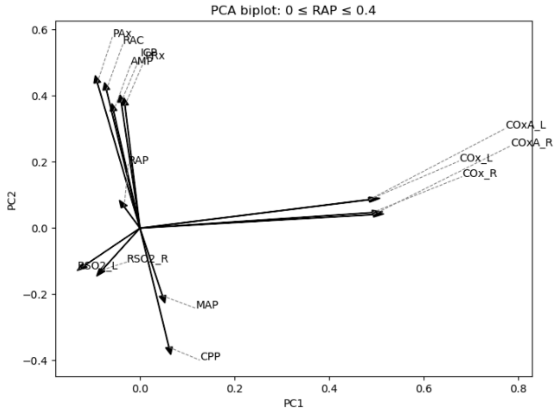

(b) ICP-ABP-derived and NIRS-derived parameters

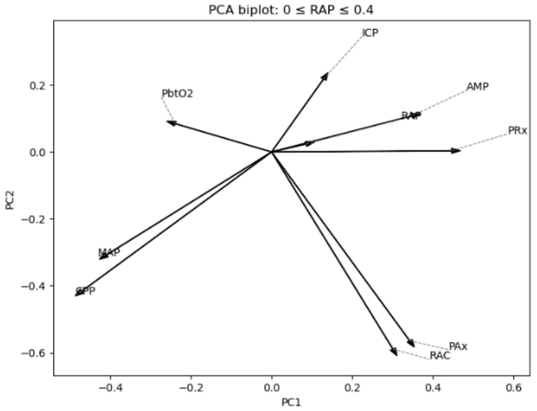

(c) ICP-ABP-derived parameters and $PbtO_2$

Figure S.25 PCA biplots at minute-by-minute resolution across RAP > 0.4 state

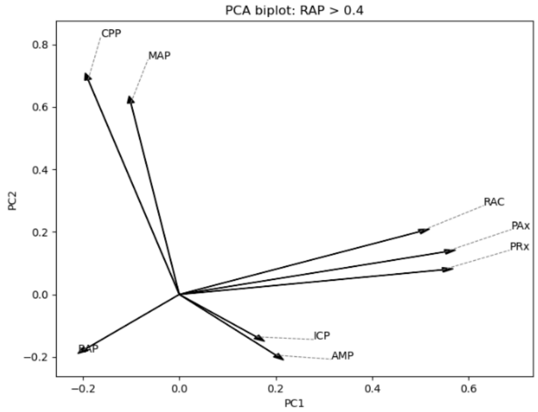

(a) ICP-ABP-derived parameters

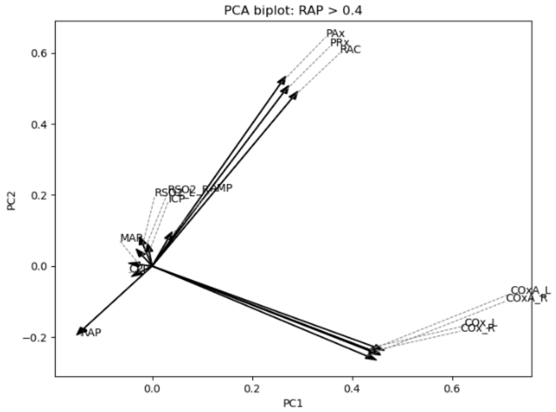

(b) ICP-ABP-derived and NIRS-derived parameters

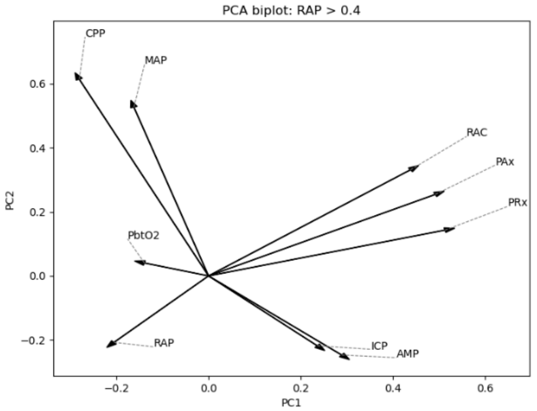

(c) ICP-ABP-derived parameters and $PbtO_2$

Figure S.26 PCA biplots at 5-minute-by-5-minute resolution across RAP > 0.4 state

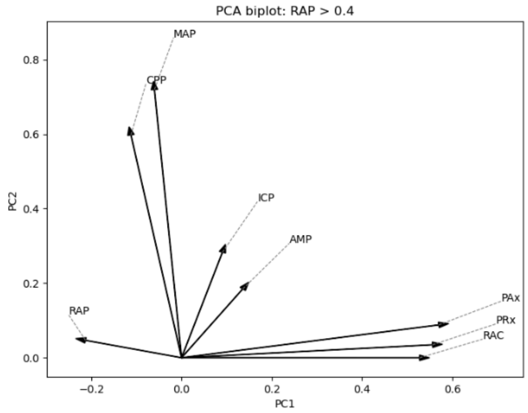

(a) ICP-ABP-derived parameters

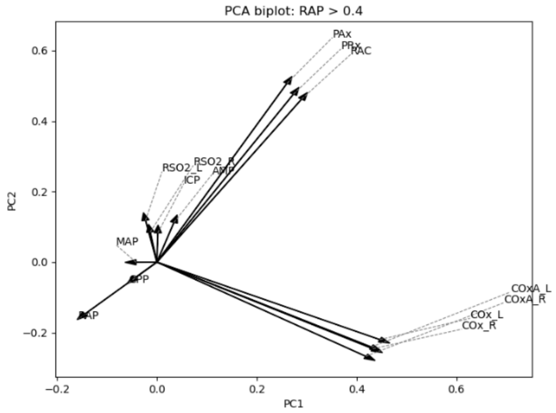

(b) ICP-ABP-derived and NIRS-derived parameters

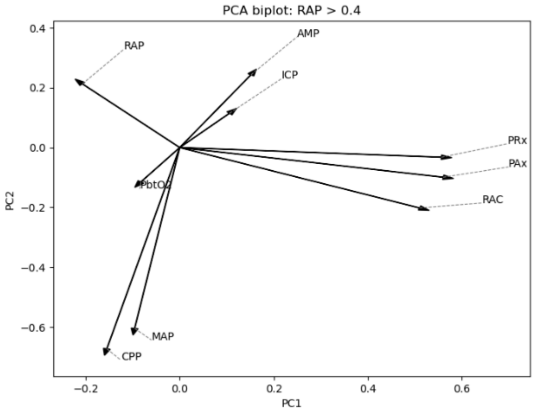

(c) ICP-ABP-derived parameters and $PbtO_2$

Figure S.27 PCA biplots at 5-minute-by-5-minute resolution across RAP > 0.4 state

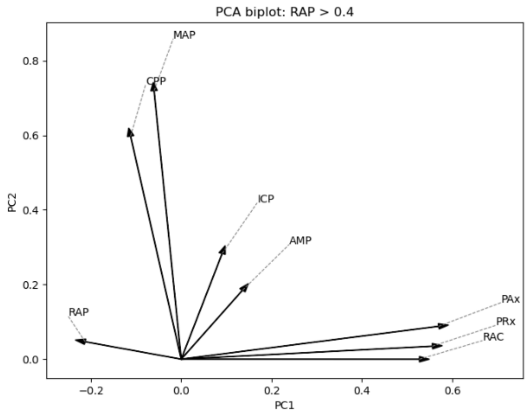

(a) ICP-ABP-derived parameters

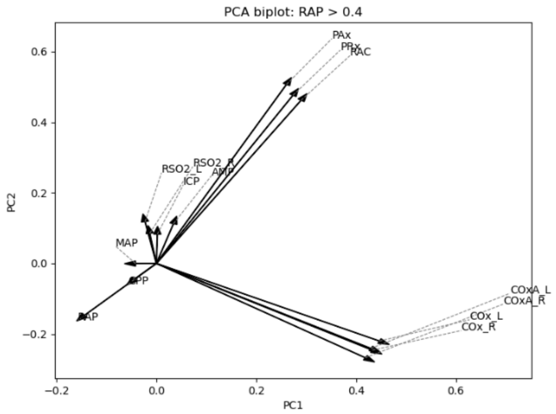

(b) ICP-ABP-derived and NIRS-derived parameters

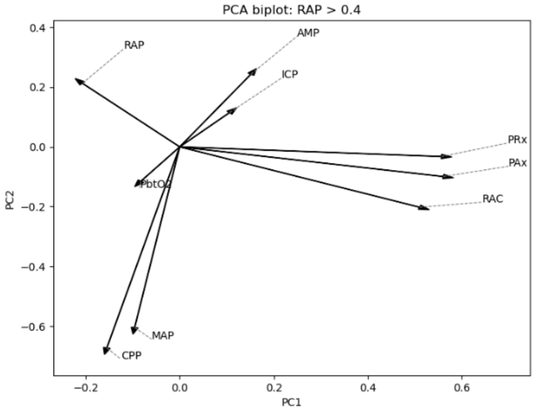

(c) ICP-ABP-derived parameters and $PbtO_2$

Figure S.28 PCA biplots at 10-minute-by-10-minute resolution across RAP > 0.4 state

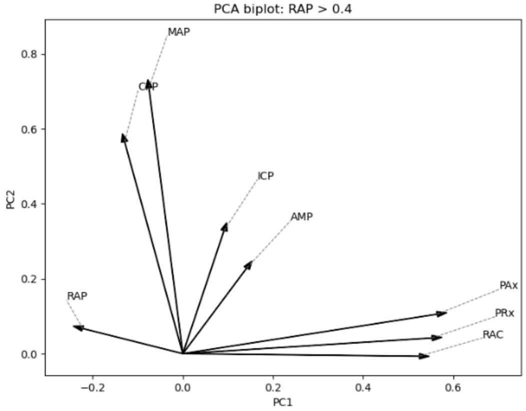

(a) ICP-ABP-derived parameters

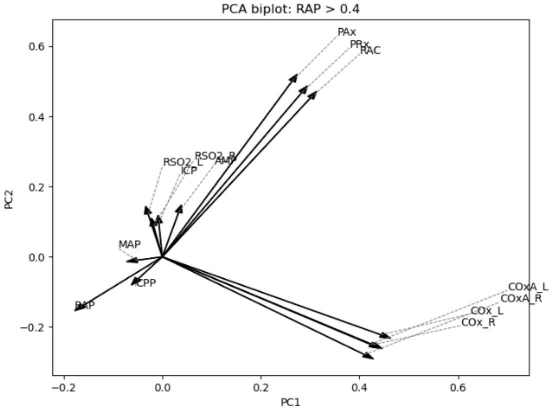

(b) ICP-ABP-derived and NIRS-derived parameters

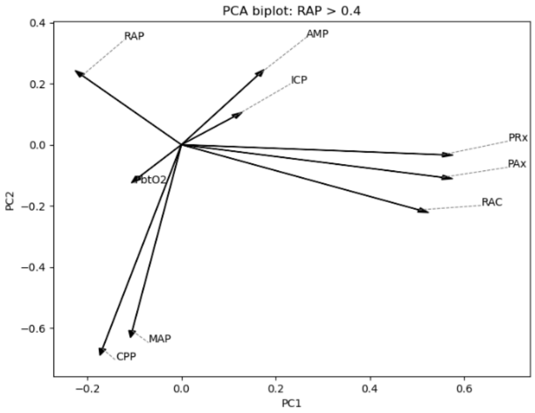

(c) ICP-ABP-derived parameters and $PbtO_2$

Figure S.29 PCA biplots at 30-minute-by-30-minute resolution across RAP > 0.4 state

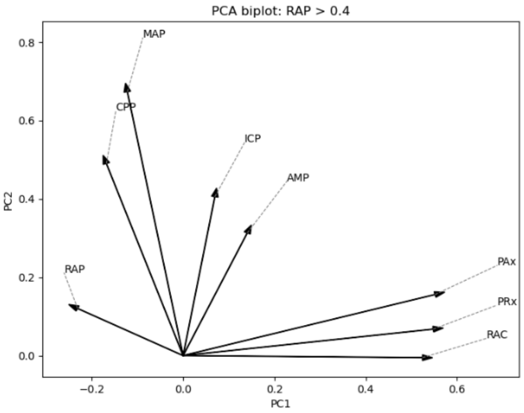

(a) ICP-ABP-derived parameters

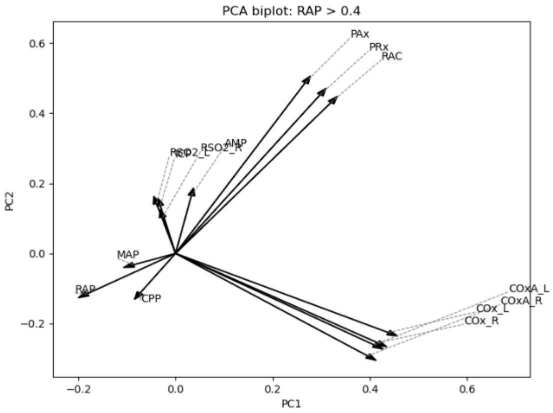

(b) ICP-ABP-derived and NIRS-derived parameters

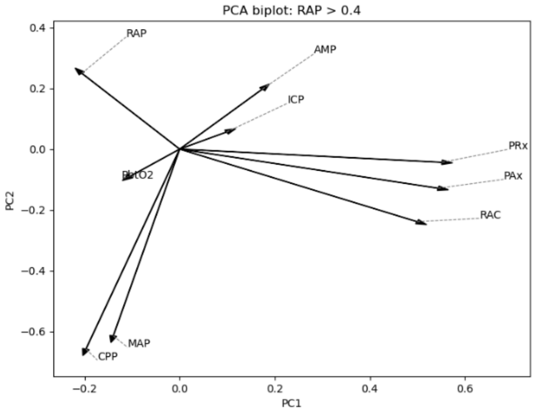

(c) ICP-ABP-derived parameters and $PbtO_2$

Figure S.30 PCA biplots at hour-by-hour resolution across RAP > 0.4 state

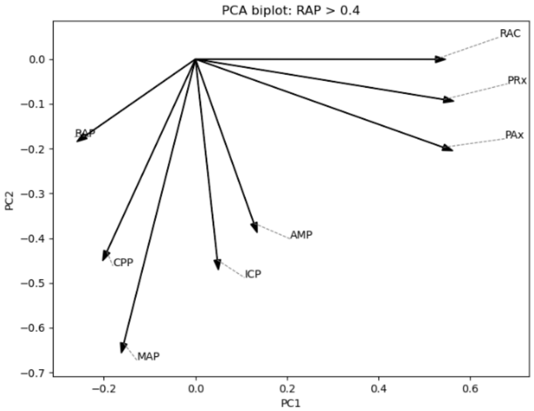

(a) ICP-ABP-derived parameters

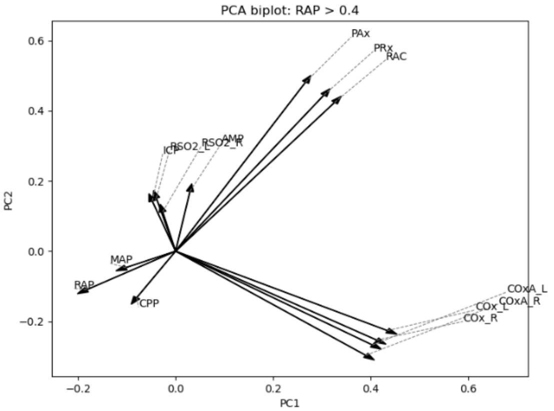

(b) ICP-ABP-derived and NIRS-derived parameters

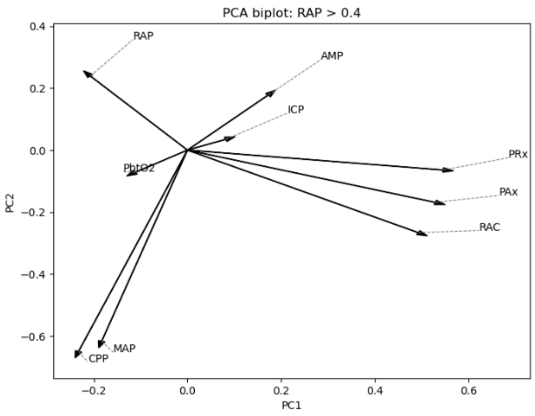

(c) ICP-ABP-derived parameters and $PbtO_2$

Supplementary D: Application of Semi-supervised Machine Learning Models – K-Means Clustering Analysis (KMCA)

This supplemantray contains the number of clusters (k) vs within-cluster sum of squared errors (WCSS) graphs for the application of the elbow method to determine the optimal k for KMCA. These plots are provided for both lower-resolution data and sub-group analyses. The KMCA results for sub-group analyses are also summarized at the end of the supplemantray.

ABP, arterial blood pressure; AMP, pulse amplitude of ICP; COx_L, cerebral oxygenation index of left hemisphere; COx_R, cerebral oxygenation index of right hemisphere; COx-a_L, COx with ABP of left hemisphere; COx-a_R, COx with ABP of the right hemisphere; CPP, cerebral perfusion pressure; ICP, intracranial pressure; MAP, mean arterial pressure; PAx, pulse amplitude index; $PbtO_2$, brain tissue oxygenation ; PRx, pressure reactivity index; RAC, a cerebral autoregulation index; RAP, index of cerebral compensatory reserve; rSO_2_L, regional cerebral oxygen saturation of left hemisphere; rSO_2_R, regional cerebral oxygen saturation of the right hemispher; WCSS, within-cluster sum of squared errors

Figure S.1 Application of the elbow method at 5-minute-by-5-minute resolution across whole population

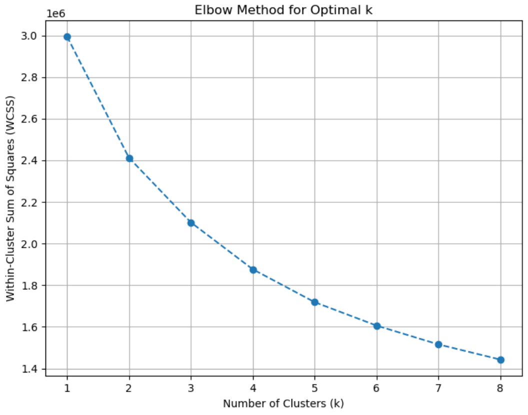

(a) ICP-ABP-derived parameters

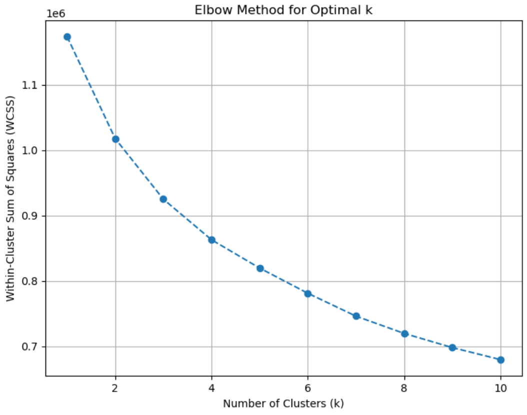

(b) ICP-ABP-derived and NIRS-derived parameters

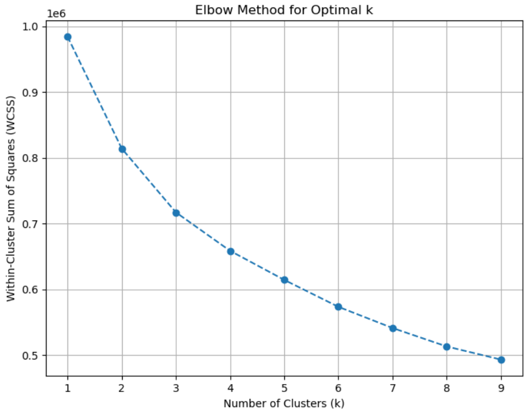

(c) ICP-ABP-derived parameters and $PbtO_2$

Figure S.2 Application of the elbow method at 10-minute-by-10-minute resolution across whole population

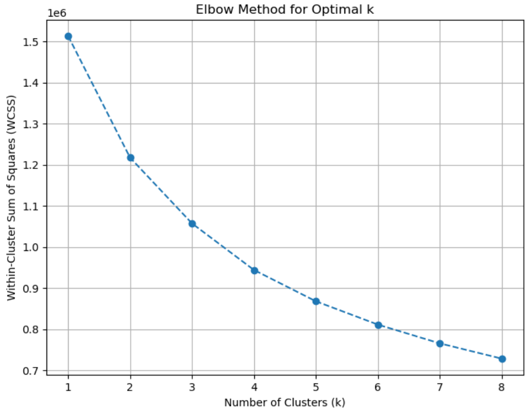

(a) ICP-ABP-derived parameters

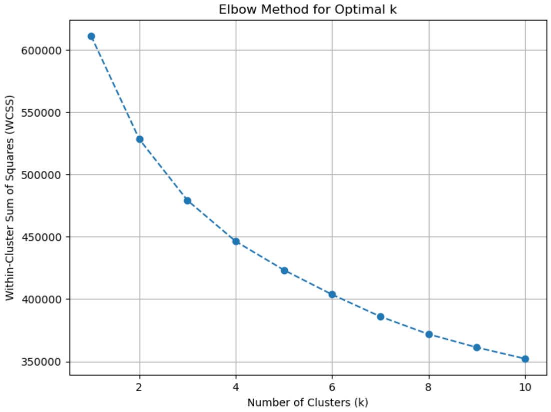

(b) ICP-ABP-derived and NIRS-derived parameters

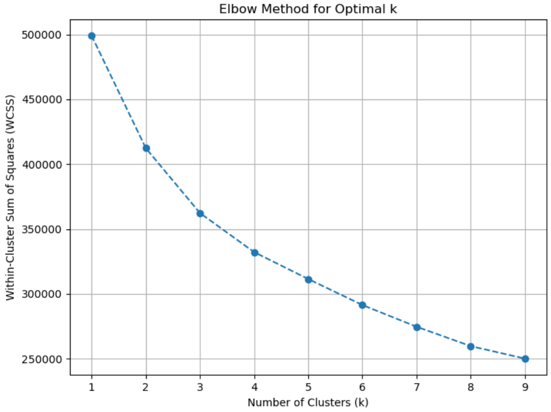

(c) ICP-ABP-derived parameters and $PbtO_2$

Figure S.3 Application of the elbow method at 30-minute-by-30-minute resolution across whole population

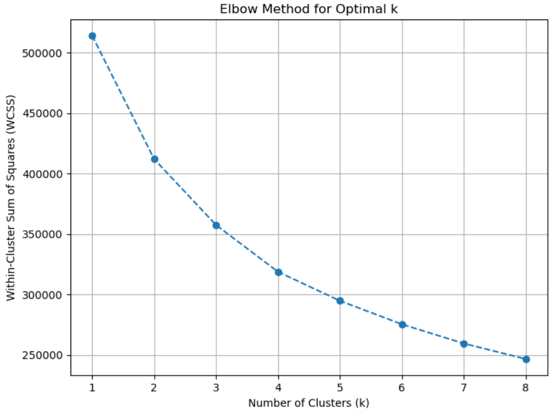

(a) ICP-ABP-derived parameters

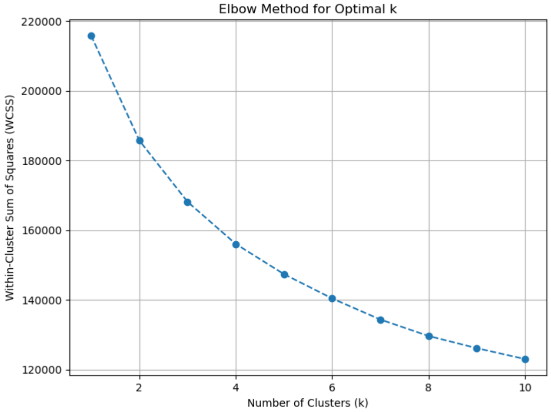

(b) ICP-ABP-derived and NIRS-derived parameters

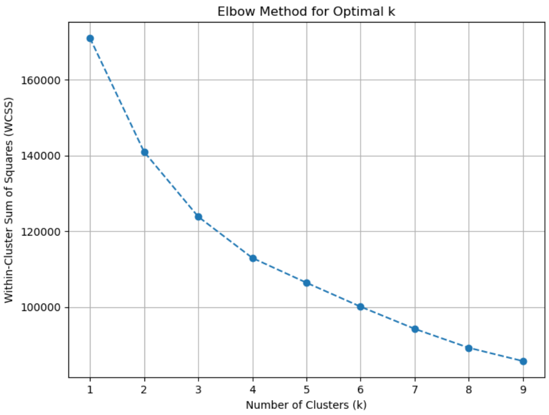

(c) ICP-ABP-derived parameters and $PbtO_2$

Figure S.4 Application of the elbow method at hour-by-hour resolution across whole population

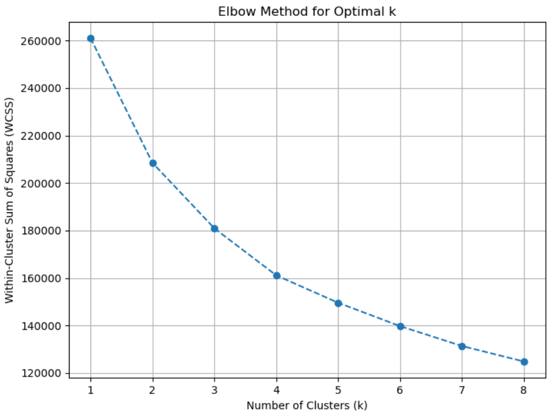

(a) ICP-ABP-derived parameters

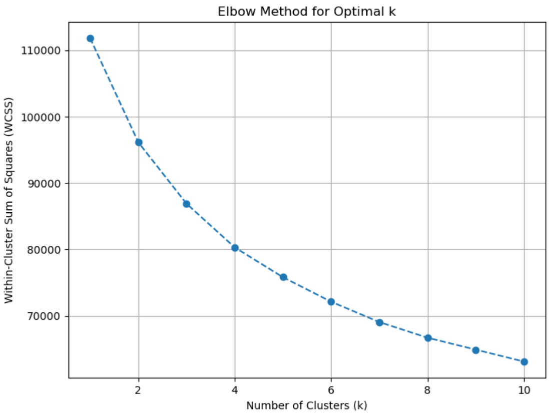

(b) ICP-ABP-derived and NIRS-derived parameters

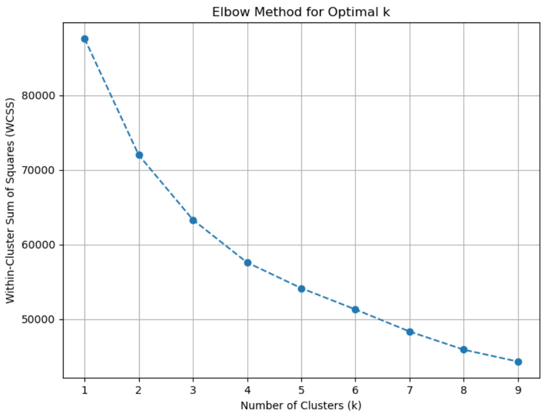

(c) ICP-ABP-derived parameters and $PbtO_2$

Figure S.5 Application of the elbow method at minute-by-minute resolution across RAP < 0 state

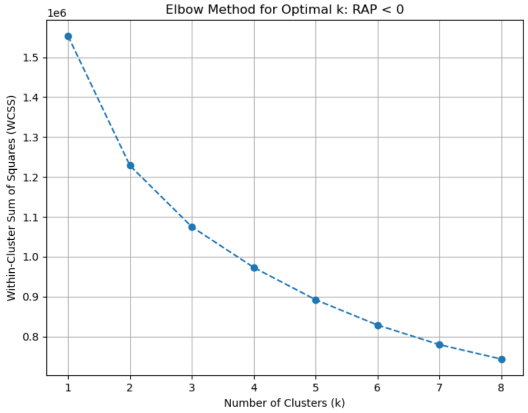

(a) ICP-ABP-derived parameters

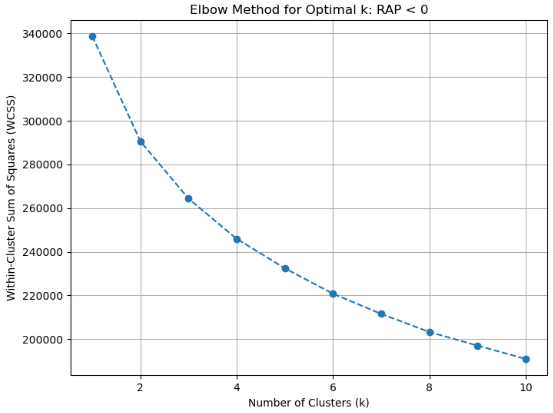

(b) ICP-ABP-derived and NIRS-derived parameters

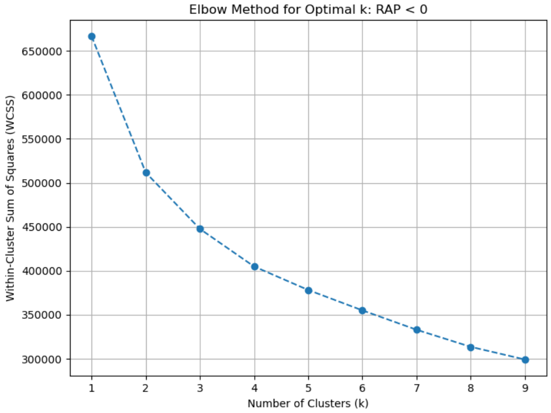

(c) ICP-ABP-derived parameters and $PbtO_2$

Figure S.6 Application of the elbow method at 5-minute-by-5-minute resolution across RAP < 0 state

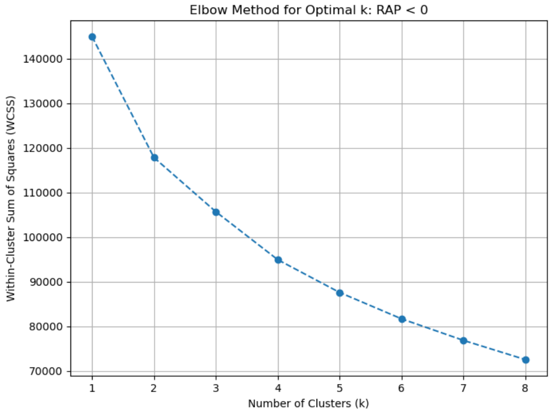

(a) ICP-ABP-derived parameters

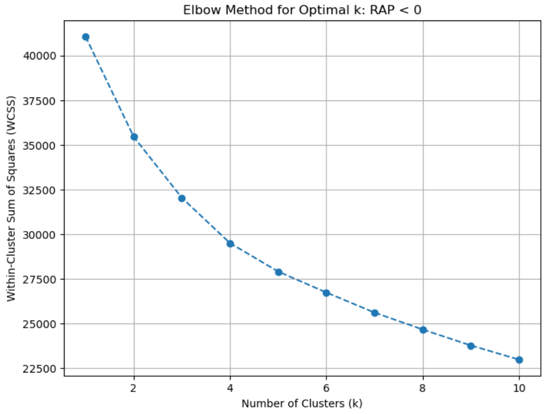

(b) ICP-ABP-derived and NIRS-derived parameters

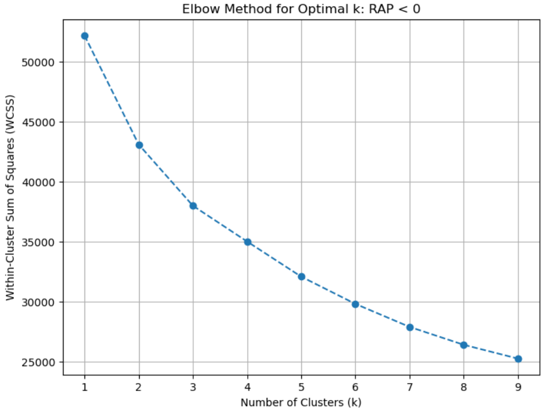

(c) ICP-ABP-derived parameters and $PbtO_2$

Figure S.7 Application of the elbow method at 10-minute-by-10-minute resolution across RAP < 0 state

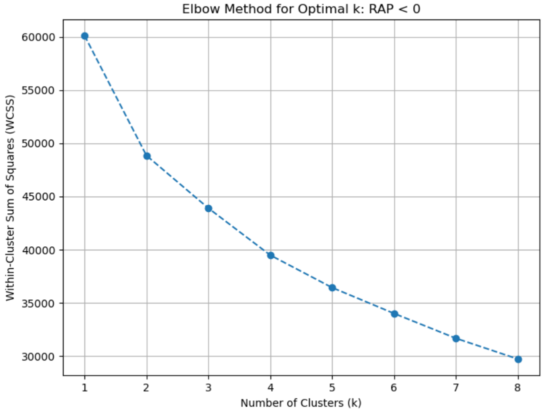

(a) ICP-ABP-derived parameters

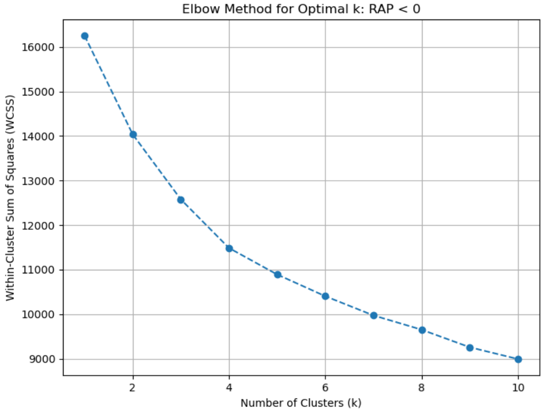

(b) ICP-ABP-derived and NIRS-derived parameters

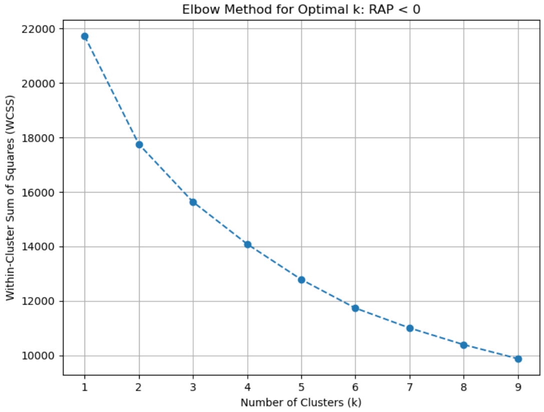

(c) ICP-ABP-derived parameters and $PbtO_2$

Figure S.8 Application of the elbow method at 30-minute-by-30-minute resolution across RAP < 0 state

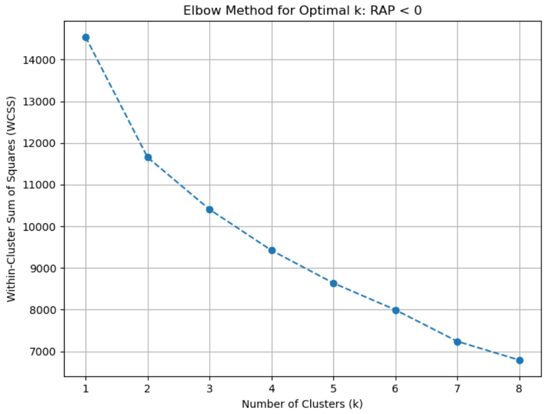

(a) ICP-ABP-derived parameters

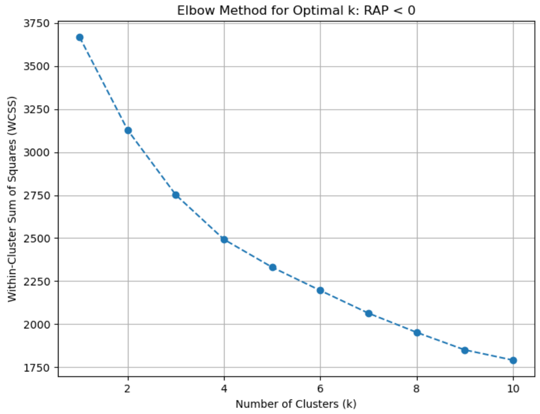

(b) ICP-ABP-derived and NIRS-derived parameters

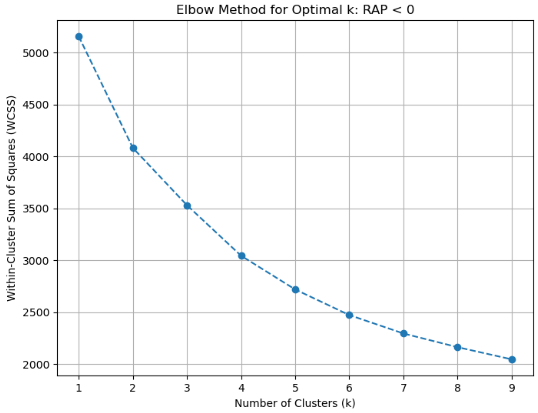

(c) ICP-ABP-derived parameters and $PbtO_2$

Figure S.9 Application of the elbow method at hour-by-hour resolution across RAP < 0 state

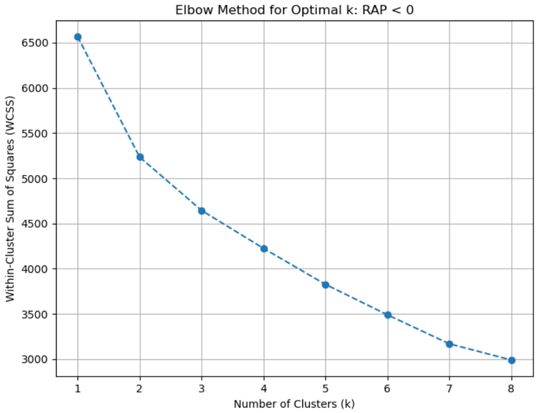

(a) ICP-ABP-derived parameters

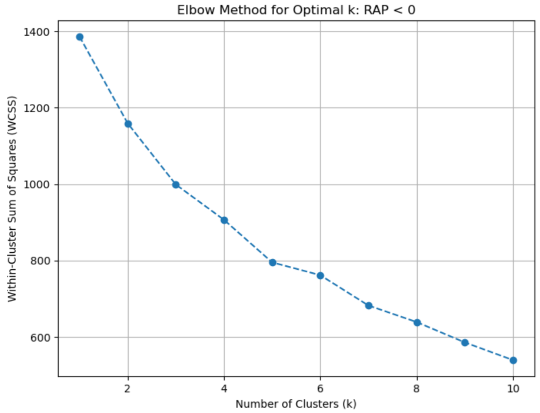

(b) ICP-ABP-derived and NIRS-derived parameters

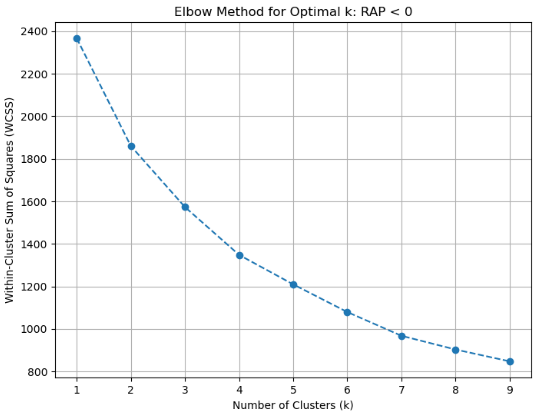

(c) ICP-ABP-derived parameters and $PbtO_2$

Figure S.10 Application of the elbow method at minute-by-minute resolution across 0 ≤ RAP ≤ 0.4 state

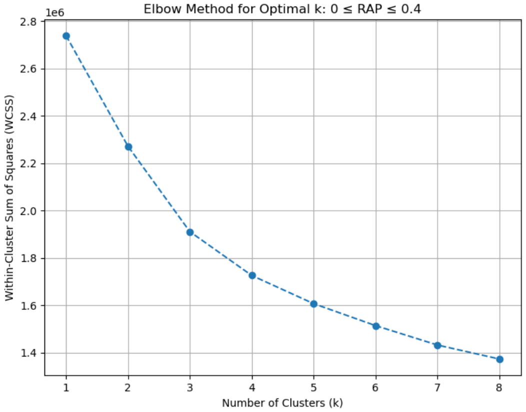

(a) ICP-ABP-derived parameters

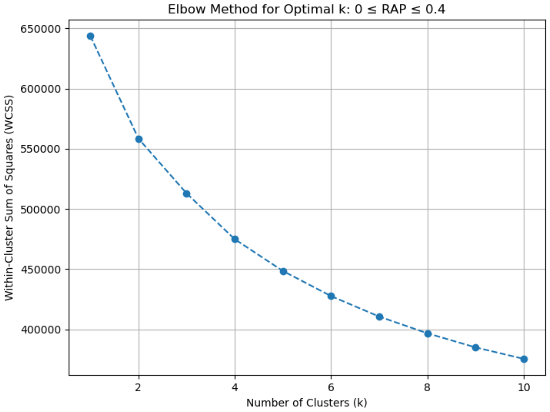

(b) ICP-ABP-derived and NIRS-derived parameters

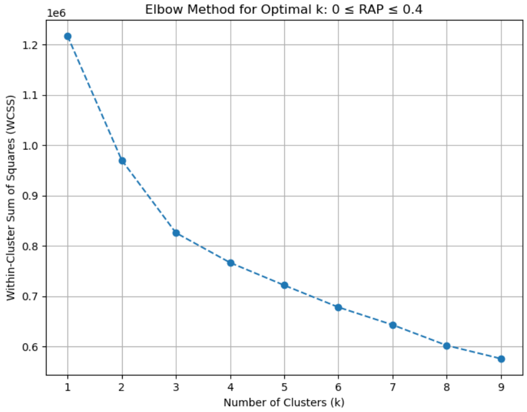

(c) ICP-ABP-derived parameters and $PbtO_2$

Figure S.11 Application of the elbow method at 5-minute-by-5-minute resolution across $0 \leq RAP \leq 0.4$ state

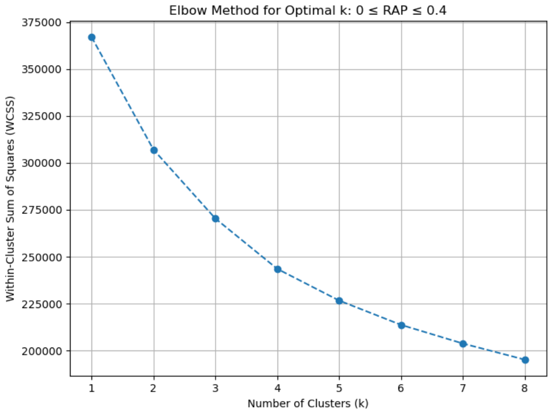

(a) ICP-ABP-derived parameters

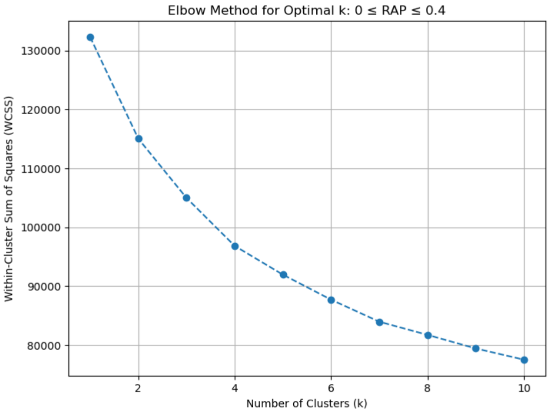

(b) ICP-ABP-derived and NIRS-derived parameters

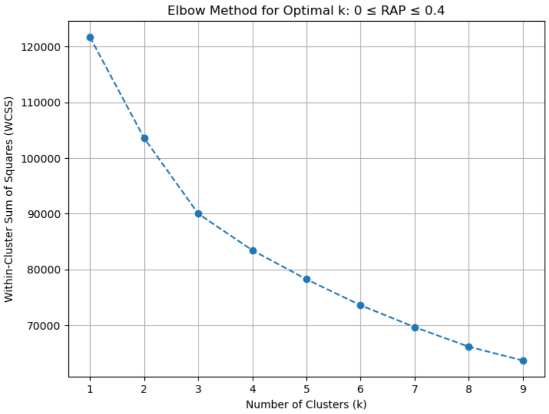

(c) ICP-ABP-derived parameters and $PbtO_2$

Figure S.12 Application of the elbow method at 10-minute-by-10-minute resolution across $0 \leq RAP \leq 0.4$ state

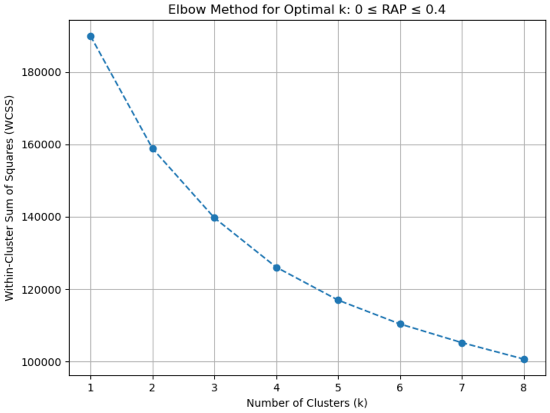

(a) ICP-ABP-derived parameters

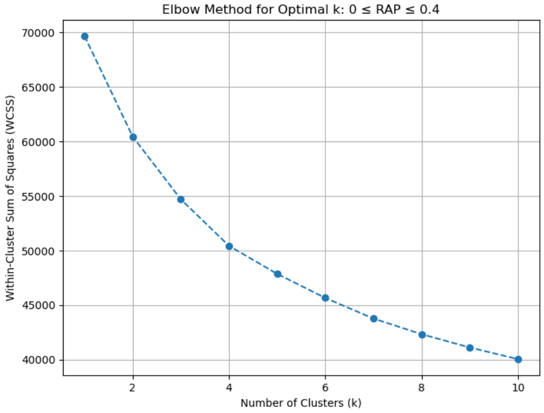

(b) ICP-ABP-derived and NIRS-derived parameters

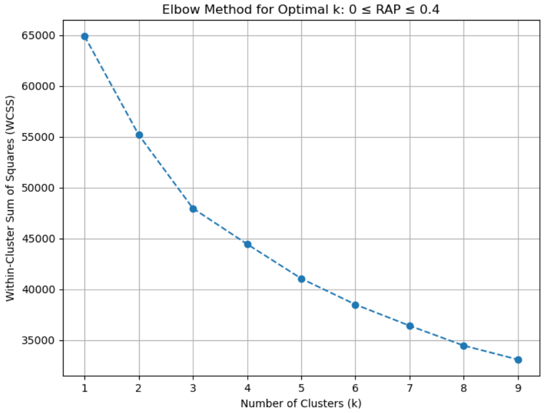

(c) ICP-ABP-derived parameters and $PbtO_2$

Figure S.13 Application of the elbow method at 30-minute-by-30-minute resolution across 0 ≤ RAP ≤ 0.4 state

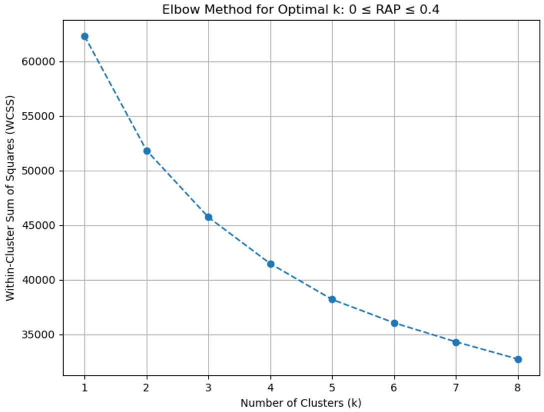

(a) ICP-ABP-derived parameters

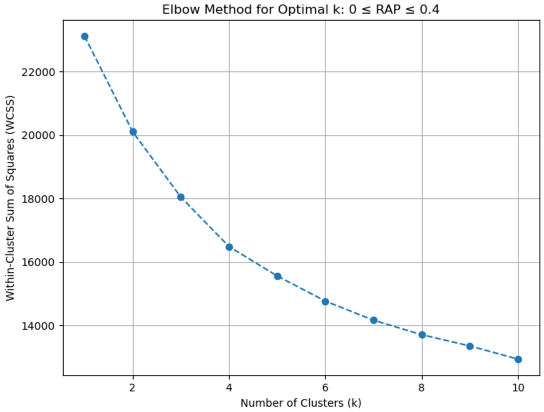

(b) ICP-ABP-derived and NIRS-derived parameters

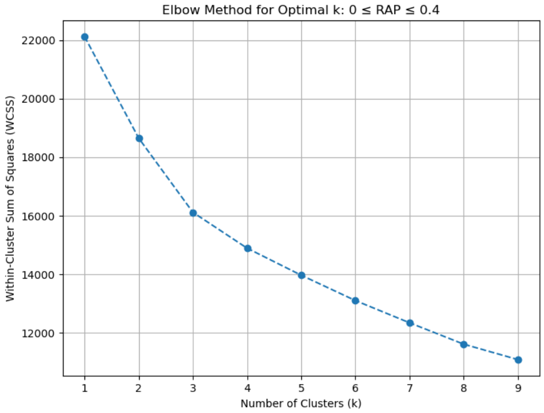

(c) ICP-ABP-derived parameters and $PbtO_2$

Figure S.14 Application of the elbow method at hour-by-hour resolution across 0 ≤ RAP ≤ 0.4 state

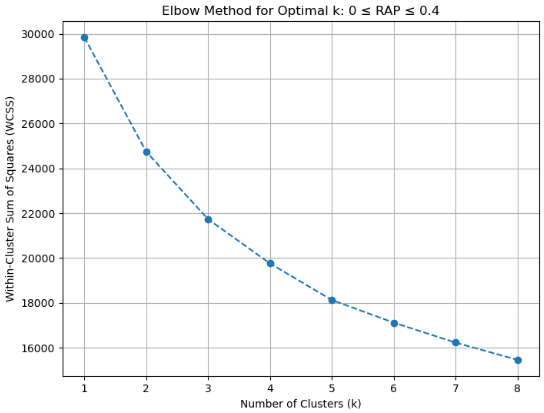

(a) ICP-ABP-derived parameters

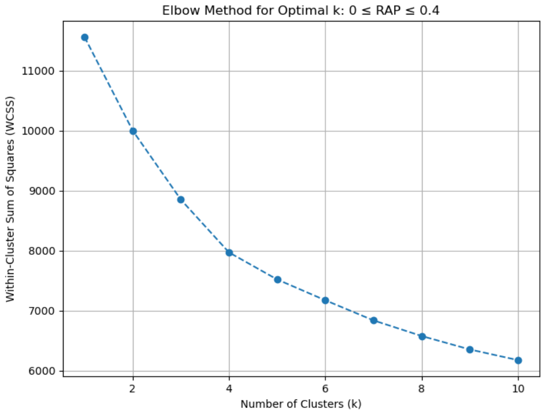

(b) ICP-ABP-derived and NIRS-derived parameters

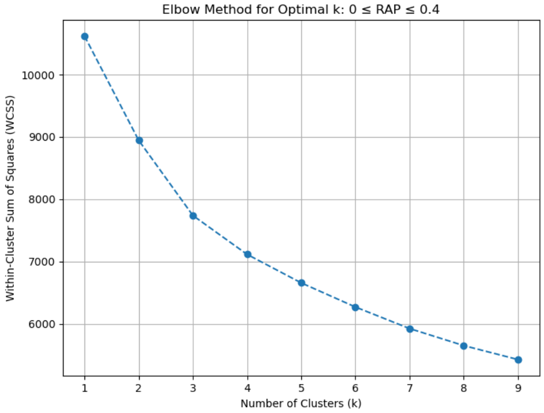

(c) ICP-ABP-derived parameters and $PbtO_2$

Figure S.15 Application of the elbow method at minute-by-minute resolution across RAP > 0.4 state

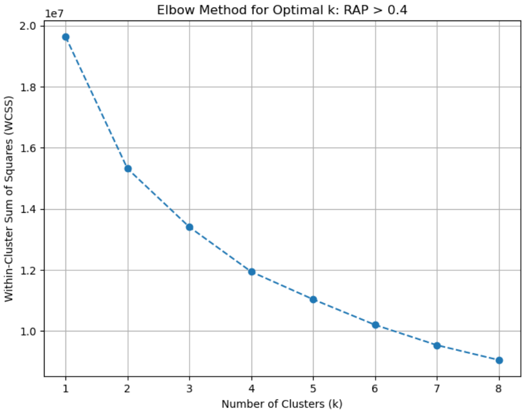

(a) ICP-ABP-derived parameters

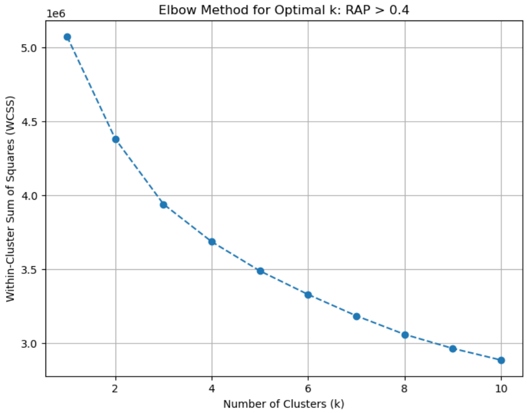

(b) ICP-ABP-derived and NIRS-derived parameters

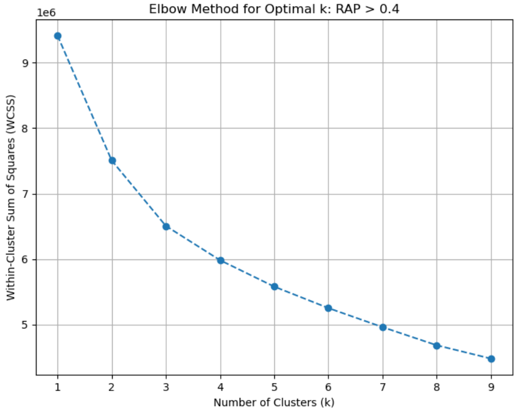

(c) ICP-ABP-derived parameters and $PbtO_2$

Figure S.16 Application of the elbow method at 5-minute-by-5-minute resolution across RAP > 0.4 state

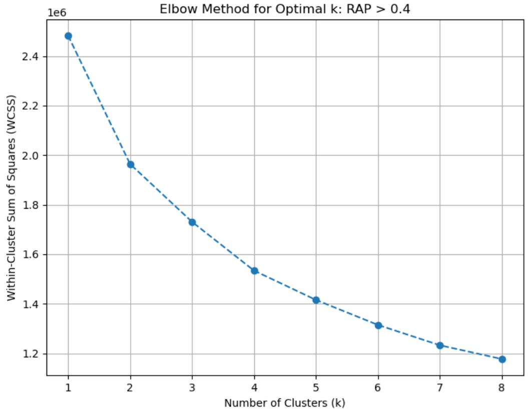

(a) ICP-ABP-derived parameters

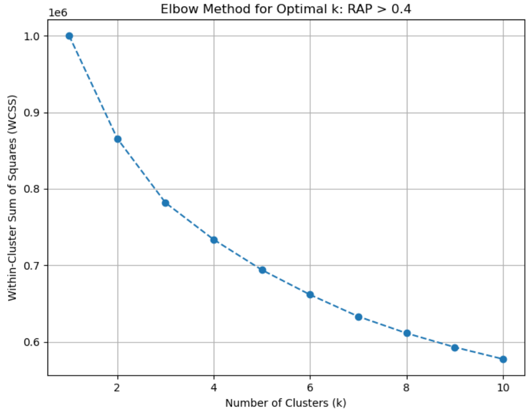

(b) ICP-ABP-derived and NIRS-derived parameters

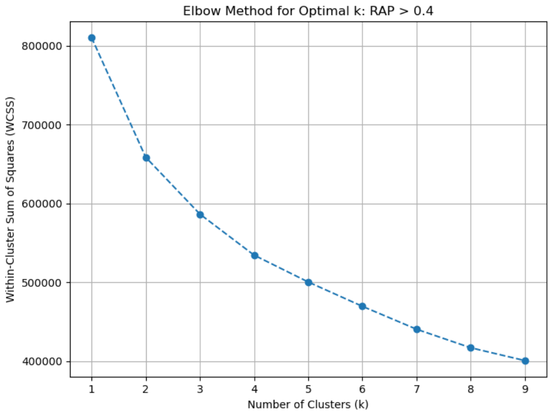

(c) ICP-ABP-derived parameters and $PbtO_2$

Figure S.17 Application of the elbow method at 10-minute-by-10-minute resolution across RAP > 0.4 state

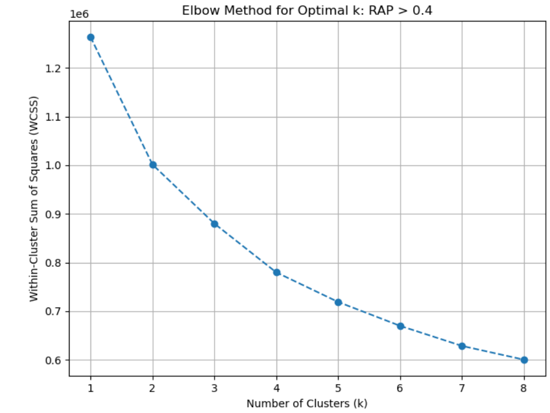

(a) ICP-ABP-derived parameters

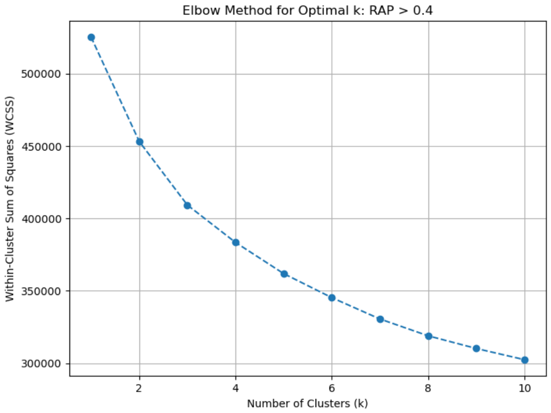

(b) ICP-ABP-derived and NIRS-derived parameters

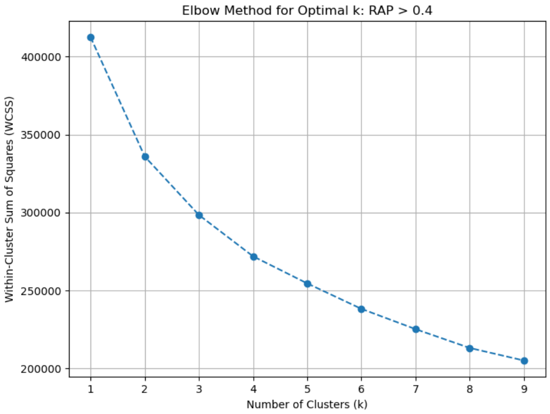

(c) ICP-ABP-derived parameters and $PbtO_2$

Figure S.18 Application of the elbow method at 30-minute-by-30-minute resolution across RAP > 0.4 state

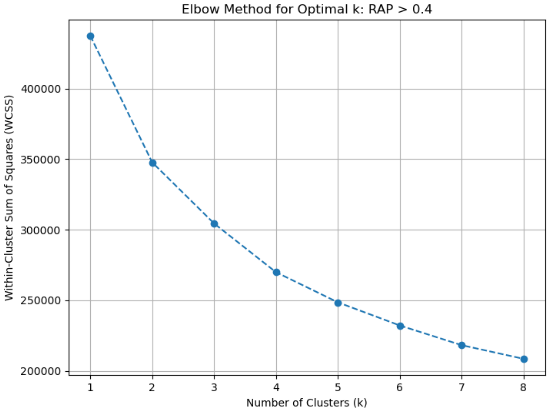

(a) ICP-ABP-derived parameters

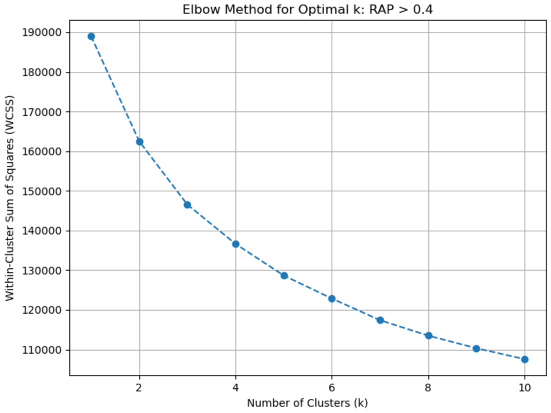

(b) ICP-ABP-derived and NIRS-derived parameters

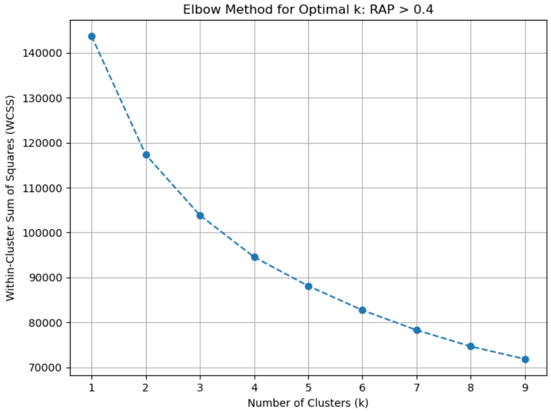

(c) ICP-ABP-derived parameters and $PbtO_2$

Figure S.19 Application of the elbow method at hour-by-hour resolution across RAP > 0.4 state

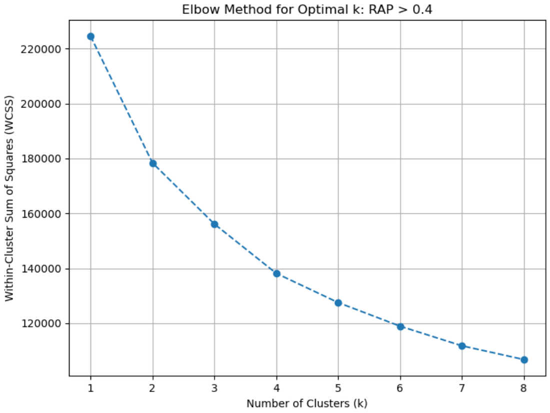

(a) ICP-ABP-derived parameters

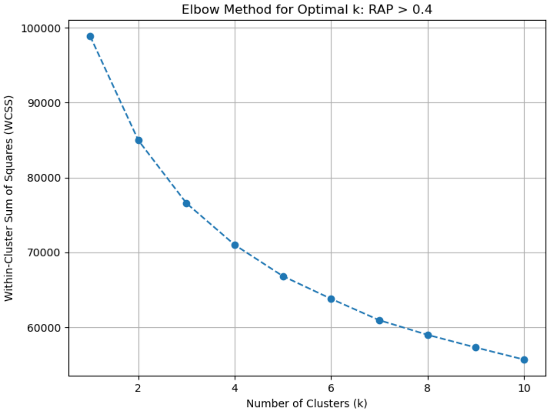

(b) ICP-ABP-derived and NIRS-derived parameters

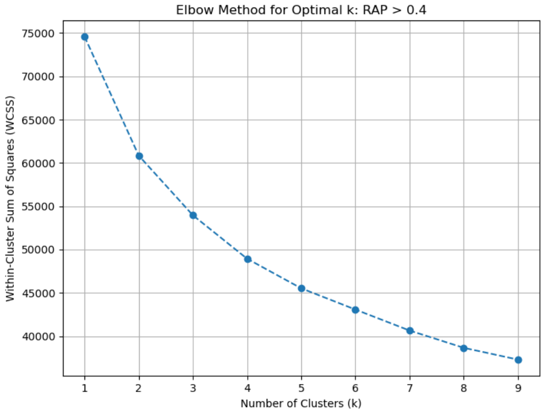

(c) ICP-ABP-derived parameters and $PbtO_2$

Table S.1 Clusters generated from KMCA across all resolutions at different RAP states

**RAP < 0 state**			
**Resolution**	**ICP-ABP-derived parameters**	**ICP-ABP-derived and NIRS-derived parameters**	**ICP-ABP-derived parameters and $PbtO_2$**
Minute-by-minute	0: ['RAP', 'AMP', 'PRx', 'PAx', 'RAC'] , 1: ['MAP', 'CPP'], 2: [ICP]	0: [rSO_2_L', rSO_2_R'], 1: ['RAP', 'ICP', 'AMP', 'PRx', 'PAx', 'RAC', 'COx_L', 'COx_R', 'COx-a_L', 'COx-a_R'], 2: ['MAP', 'CPP']	0: ['RAP', 'AMP', 'PRx', 'PAx', 'RAC'], 1: ['MAP', 'CPP'], 2: ['ICP', 'PbtO2']
5-minute-by-5-minute	0: ['ICP'], 1: ['MAP', 'CPP'], 2: ['RAP', 'AMP', 'PRx', 'PAx', 'RAC']	0: [rSO_2_L', rSO_2_R'], 1: ['RAP', 'ICP', 'AMP', 'PRx', 'PAx', 'RAC', 'COx_L', 'COx_R', 'COx-a_L', 'COx-a_R'], 2: ['MAP', 'CPP']	0: ['ICP', $PbtO_2$], 1: ['MAP', 'CPP'], 2: ['RAP', 'AMP', 'PRx', 'PAx', 'RAC']
10-minute-by-10-minute	0: ['ICP'], 1: ['MAP', 'CPP'], 2: ['RAP', 'AMP', 'PRx', 'PAx', 'RAC']	0: [rSO_2_L', rSO_2_R'], 1: ['RAP', 'ICP', 'AMP', 'PRx', 'PAx', 'RAC', 'COx_L', 'COx_R', 'COx-a_L', 'COx-a_R'], 2: ['MAP', 'CPP']	0: ['ICP', $PbtO_2$], 1: ['MAP', 'CPP'], 2: ['RAP', 'AMP', 'PRx', 'PAx', 'RAC']
30-minute-by-30-minute	0: ['ICP'], 1: ['MAP', 'CPP'], 2: ['RAP', 'AMP', 'PRx', 'PAx', 'RAC']	0: [rSO_2_L', rSO_2_R'], 1: ['RAP', 'ICP', 'AMP', 'PRx', 'PAx', 'RAC', 'COx_L', 'COx_R', 'COx-a_L', 'COx-a_R'], 2: ['MAP', 'CPP']	0: ['ICP', $PbtO_2$], 1: ['MAP', 'CPP'], 2: ['RAP', 'AMP', 'PRx', 'PAx', 'RAC']
Hour-by-hour	0: ['ICP'], 1: ['MAP', 'CPP'], 2: ['RAP', 'AMP', 'PRx', 'PAx', 'RAC']	0: [rSO_2_L', rSO_2_R'], 1: ['RAP', 'ICP', 'AMP', 'PRx', 'PAx', 'RAC', 'COx_L', 'COx_R', 'COx-a_L', 'COx-a_R'], 2: ['MAP', 'CPP']	0: ['RAP', 'AMP', 'PRx', 'PAx', 'RAC'], 1: ['MAP', 'CPP'], 2: ['ICP', 'PbtO2']
**0 ≤ RAP ≤ 0.4 state**			
**Resolution**	**ICP-ABP-derived parameters**	**ICP-ABP-derived and NIRS-derived parameters**	**ICP-ABP-derived parameters and $PbtO_2$**
Minute-by-minute	0: ['ICP'], 1: ['MAP', 'CPP'], 2: ['RAP', 'AMP', 'PRx', 'PAx', 'RAC']	0: [rSO_2_L', rSO_2_R'], 1: ['RAP', 'ICP', 'AMP', 'PRx', 'PAx', 'RAC', 'COx_L', 'COx_R', 'COx-a_L', 'COx-a_R'], 2: ['MAP', 'CPP']	0: ['RAP', 'AMP', 'PRx', 'PAx', 'RAC'], 1: ['MAP', 'CPP'], 2: ['ICP', 'PbtO2']
5-minute-by-5-minute	0: ['ICP'], 1: ['MAP', 'CPP'], 2: ['RAP', 'AMP', 'PRx', 'PAx', 'RAC']	0: [rSO_2_L', rSO_2_R'], 1: ['RAP', 'ICP', 'AMP', 'PRx', 'PAx', 'RAC', 'COx_L', 'COx_R', 'COx-a_L', 'COx-a_R'], 2: ['MAP', 'CPP']	0: ['ICP', $PbtO_2$], 1: ['MAP', 'CPP'], 2: ['RAP', 'AMP', 'PRx', 'PAx', 'RAC']
10-minute-by-10-minute	0: ['ICP'], 1: ['MAP', 'CPP'], 2: ['RAP', 'AMP', 'PRx', 'PAx', 'RAC']	0: [rSO_2_L', rSO_2_R'], 1: ['RAP', 'ICP', 'AMP', 'PRx', 'PAx', 'RAC', 'COx_L', 'COx_R', 'COx-a_L', 'COx-a_R'], 2: ['MAP', 'CPP']	0: ['ICP', $PbtO_2$], 1: ['MAP', 'CPP'], 2: ['RAP', 'AMP', 'PRx', 'PAx', 'RAC']
30-minute-by-30-minute	0: ['ICP'], 1: ['MAP', 'CPP'], 2: ['RAP', 'AMP', 'PRx', 'PAx', 'RAC']	0: [rSO_2_L', rSO_2_R'], 1: ['RAP', 'ICP', 'AMP', 'PRx', 'PAx', 'RAC', 'COx_L', 'COx_R', 'COx-a_L', 'COx-a_R'], 2: ['MAP', 'CPP']	0: ['ICP', $PbtO_2$], 1: ['MAP', 'CPP'], 2: ['RAP', 'AMP', 'PRx', 'PAx', 'RAC']
Hour-by-hour	0: ['ICP'], 1: ['MAP', 'CPP'], 2: ['RAP', 'AMP', 'PRx', 'PAx', 'RAC']	0: [rSO_2_L', rSO_2_R'], 1: ['RAP', 'ICP', 'AMP', 'PRx', 'PAx', 'RAC', 'COx_L', 'COx_R', 'COx-a_L', 'COx-a_R'], 2: ['MAP', 'CPP']	0: ['ICP', $PbtO_2$], 1: ['MAP', 'CPP'], 2: ['RAP', 'AMP', 'PRx', 'PAx', 'RAC']
**RAP > 0.4 state**			
**Resolution**	**ICP-ABP-derived parameters**	**ICP-ABP-derived and NIRS-derived parameters**	**ICP-ABP-derived parameters and $PbtO_2$**
Minute-by-minute	0: ['ICP'], 1: ['MAP', 'CPP'], 2: ['RAP', 'AMP', 'PRx', 'PAx', 'RAC']	0: [rSO_2_L', rSO_2_R'], 1: ['RAP', 'ICP', 'AMP', 'PRx', 'PAx', 'RAC', 'COx_L', 'COx_R', 'COx-a_L', 'COx-a_R'], 2: ['MAP', 'CPP']	0: ['ICP', $PbtO_2$], 1: ['MAP', 'CPP'], 2: ['RAP', 'AMP', 'PRx', 'PAx', 'RAC']

5-minute-by-5-minute	0: ['ICP'], 1: ['MAP', 'CPP'], 2: ['RAP', 'AMP', 'PRx', 'PAx', 'RAC']	0: [rSO_2_L', rSO_2_R'], 1: ['RAP', 'ICP', 'AMP', 'PRx', 'PAx', 'RAC', 'COx_L', 'COx_R', 'COx-a_L', 'COx-a_R'], 2: ['MAP', 'CPP']	0: ['ICP', $PbtO_2$], 1: ['MAP', 'CPP'], 2: ['RAP', 'AMP', 'PRx', 'PAx', 'RAC']
10-minute-by-10-minute	0: ['ICP'], 1: ['MAP', 'CPP'], 2: ['RAP', 'AMP', 'PRx', 'PAx', 'RAC']	0: [rSO_2_L', rSO_2_R'], 1: ['RAP', 'ICP', 'AMP', 'PRx', 'PAx', 'RAC', 'COx_L', 'COx_R', 'COx-a_L', 'COx-a_R'], 2: ['MAP', 'CPP']	0: ['ICP', $PbtO_2$], 1: ['MAP', 'CPP'], 2: ['RAP', 'AMP', 'PRx', 'PAx', 'RAC']
30-minute-by-30-minute	0: ['ICP'], 1: ['MAP', 'CPP'], 2: ['RAP', 'AMP', 'PRx', 'PAx', 'RAC']	0: [rSO_2_L', rSO_2_R'], 1: ['RAP', 'ICP', 'AMP', 'PRx', 'PAx', 'RAC', 'COx_L', 'COx_R', 'COx-a_L', 'COx-a_R'], 2: ['MAP', 'CPP']	0: ['ICP', $PbtO_2$], 1: ['MAP', 'CPP'], 2: ['RAP', 'AMP', 'PRx', 'PAx', 'RAC']
Hour-by-hour	0: ['ICP'], 1: ['MAP', 'CPP'], 2: ['RAP', 'AMP', 'PRx', 'PAx', 'RAC']	0: [rSO_2_L', rSO_2_R'], 1: ['RAP', 'ICP', 'AMP', 'PRx', 'PAx', 'RAC', 'COx_L', 'COx_R', 'COx-a_L', 'COx-a_R'], 2: ['MAP', 'CPP']	0: ['ICP', $PbtO_2$], 1: ['MAP', 'CPP'], 2: ['RAP', 'AMP', 'PRx', 'PAx', 'RAC']

Supplementary E: Application of Vector Autoregressive Integrated Moving Average (VARIMA) Impulse Response Function (IRF) Analysis

This supplemantray contains the IRF plots generated from the orthogonal impulse physiological parameters to RAP. Each IRF plot also demonstrates the 95% confidence interval range/boundary with red dashed lines. Prior to IRF analysis, the VARIMA optimal model was required to be calculated. The median optimal VARIMA models for each RAP-X pair (where X is other physiological parameters) are also depicted in this appendix. The results of the subgroup-level analysis for the percentage of RAP responsiveness are summarized in a table located at the end of the supplemantray.

Δ, first-order differenced; AMP, pulse amplitude of ICP; COx_L, cerebral oxygenation index of left hemisphere; COx_R, cerebral oxygenation index of right hemisphere; COx-a_L, COx with ABP of left hemisphere; COx-a_R, COx with ABP of the right hemisphere; CPP, cerebral perfusion pressure; ICP, intracranial pressure; MAP, mean arterial pressure; NA, not applicable, PAx, pulse amplitude index; $PbtO_2$, brain tissue oxygenation ; PRx, pressure reactivity index; RAC, a cerebral autoregulation index; RAP, index of cerebral compensatory reserve; rSO_2_L, regional cerebral oxygen saturation of left hemisphere; rSO_2_R, regional cerebral oxygen saturation of the right hemisphere.

Table S.1 Median optimal VARIMA models across whole population

Parameter pair	Minute-by-minute	5-minute-by-5-minute	10-minute-by-10-minute
RAP-ICP	[10, 1, 10]	[6, 1, 6]	[4, 1, 5]
RAP-AMP	[10, 1, 9]	[6, 1, 5]	[4, 1, 5]
RAP-MAP	[10, 1, 10]	[6, 1, 6]	[4, 1, 5]
RAP-CPP	[10, 1, 10]	[6, 1, 6]	[4, 1, 5]
RAP-PRx	[10, 1, 6]	[5, 1, 6]	[4, 1, 5]
RAP-PAx	[10, 1, 6]	[6, 1, 6]	[4, 1, 5]
RAP-RAC	[10, 1, 6]	[3, 1, 3]	[2, 1, 3]
RAP-rSO_2_L	[10, 1, 10]	[4, 1, 4]	[3, 1, 3]
RAP-rSO_2_R	[10, 1, 9]	[4, 1, 4]	[3, 1, 3]
RAP-COx_L	[10, 1, 6]	[4, 1, 4]	[2, 1, 3]
RAP-COx_R	[10, 1, 6]	[3, 1, 3]	[2, 1, 3]
RAP-COx-a_L	[10, 1, 6]	[4, 1, 4]	[2, 1, 3]
RAP-COx-a_R	[10, 1, 7]	[6, 1, 6]	[4, 1, 5]
RAP-$PbtO_2$	[10, 1, 10]	[5, 1, 4]	[3, 1, 4]

Figure S.1 VARIMA IRF plots at minute-by-minute resolution for patient CAHR-101

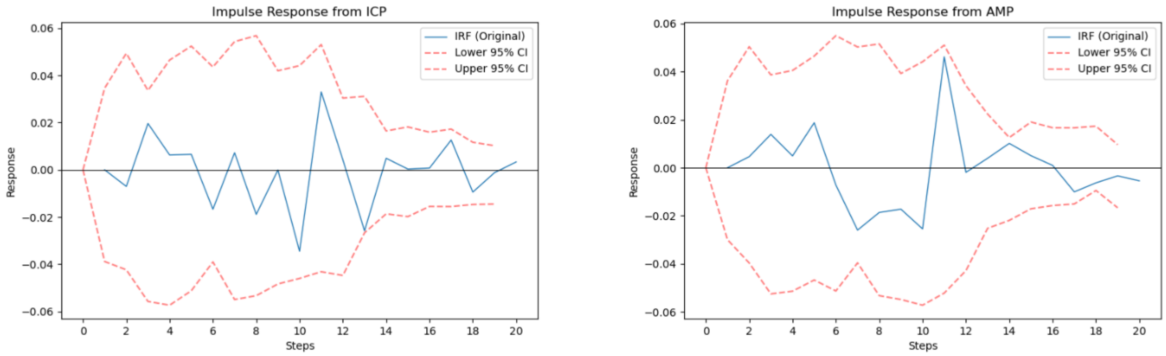

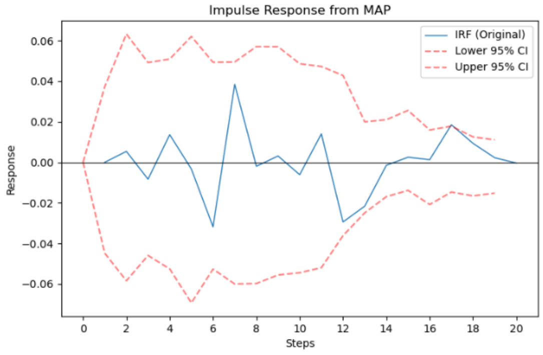
Impulse Response from MAP
IRF (Original)
Lower 95% CI
Upper 95% CI
Response
Steps

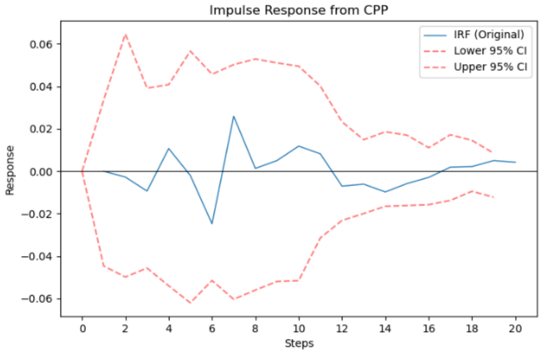
Impulse Response from CPP
IRF (Original)
Lower 95% CI
Upper 95% CI
Response
Steps

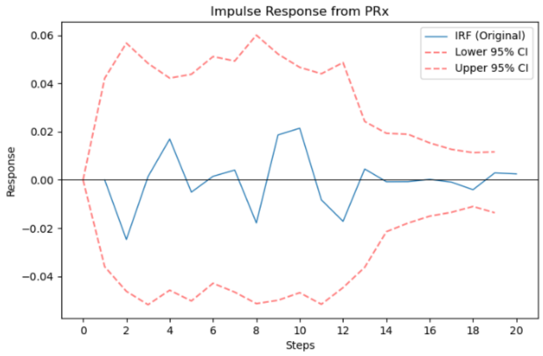
Impulse Response from PRx
IRF (Original)
Lower 95% CI
Upper 95% CI
Response
Steps

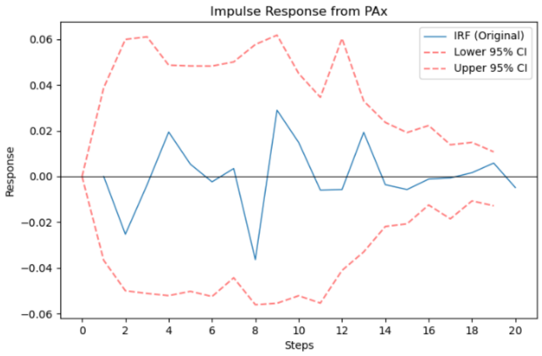
Impulse Response from PAx
IRF (Original)
Lower 95% CI
Upper 95% CI
Response
Steps

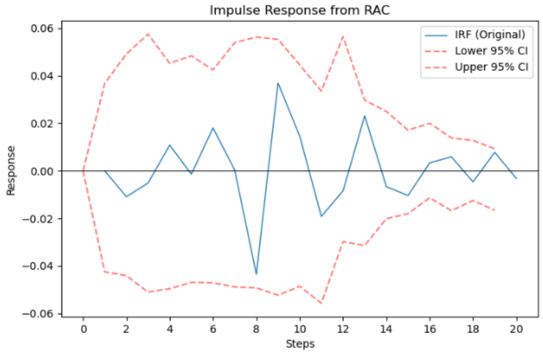
Impulse Response from RAC
IRF (Original)
Lower 95% CI
Upper 95% CI
Response
Steps

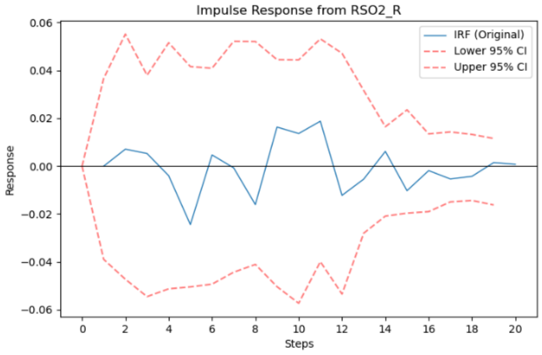
Impulse Response from RSO2_R
IRF (Original)
Lower 95% CI
Upper 95% CI
Response
Steps

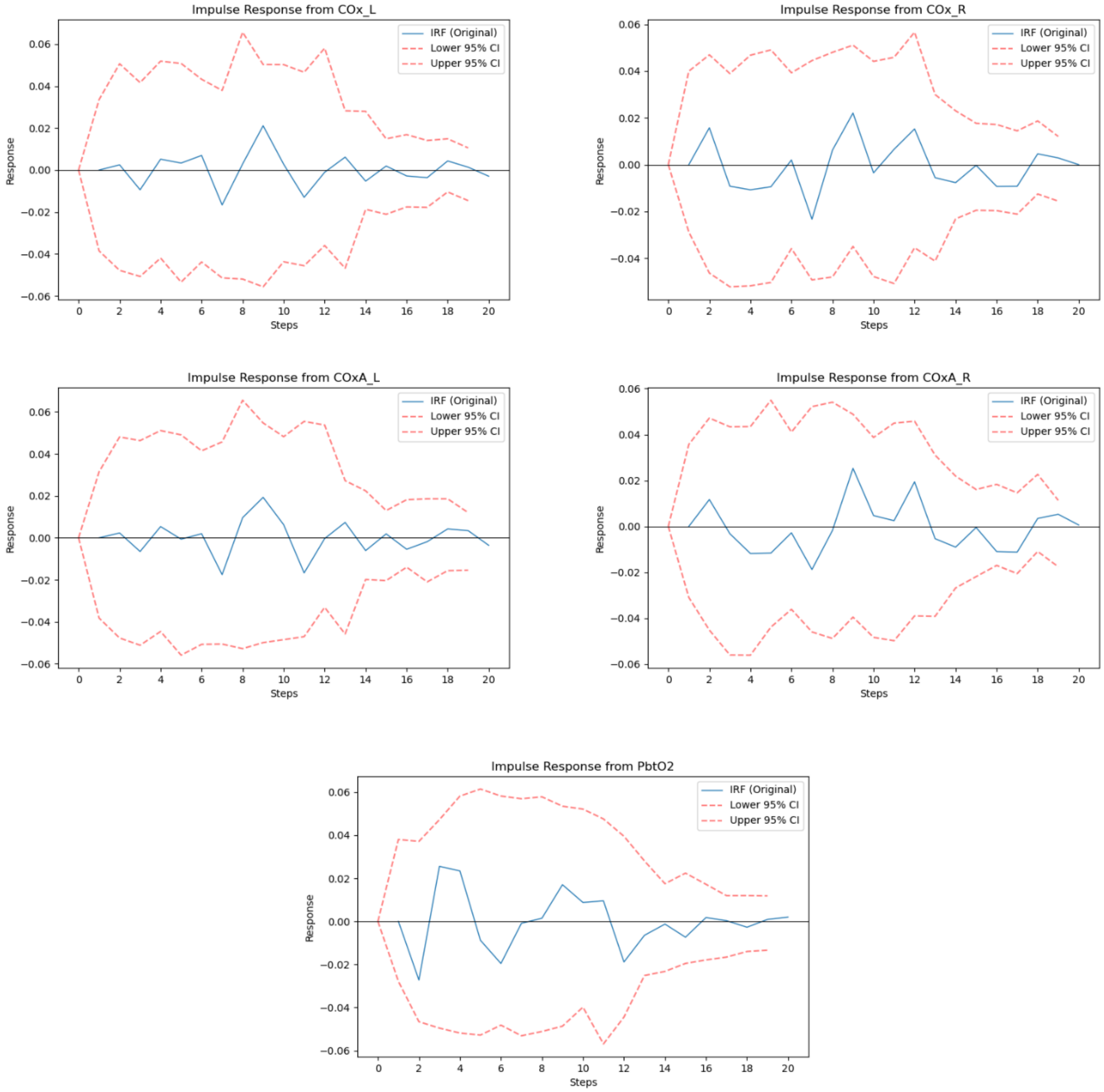

In this figure, the impulse response for rSO₂_L is absent because the corresponding VARIMA model failed to fit the data due to an insufficient number of data points, resulting in an error and no output from the analysis..

Figure S.2 VARIMA IRF plots at 5-minute-by-5-minute resolution for patient CAHR-101

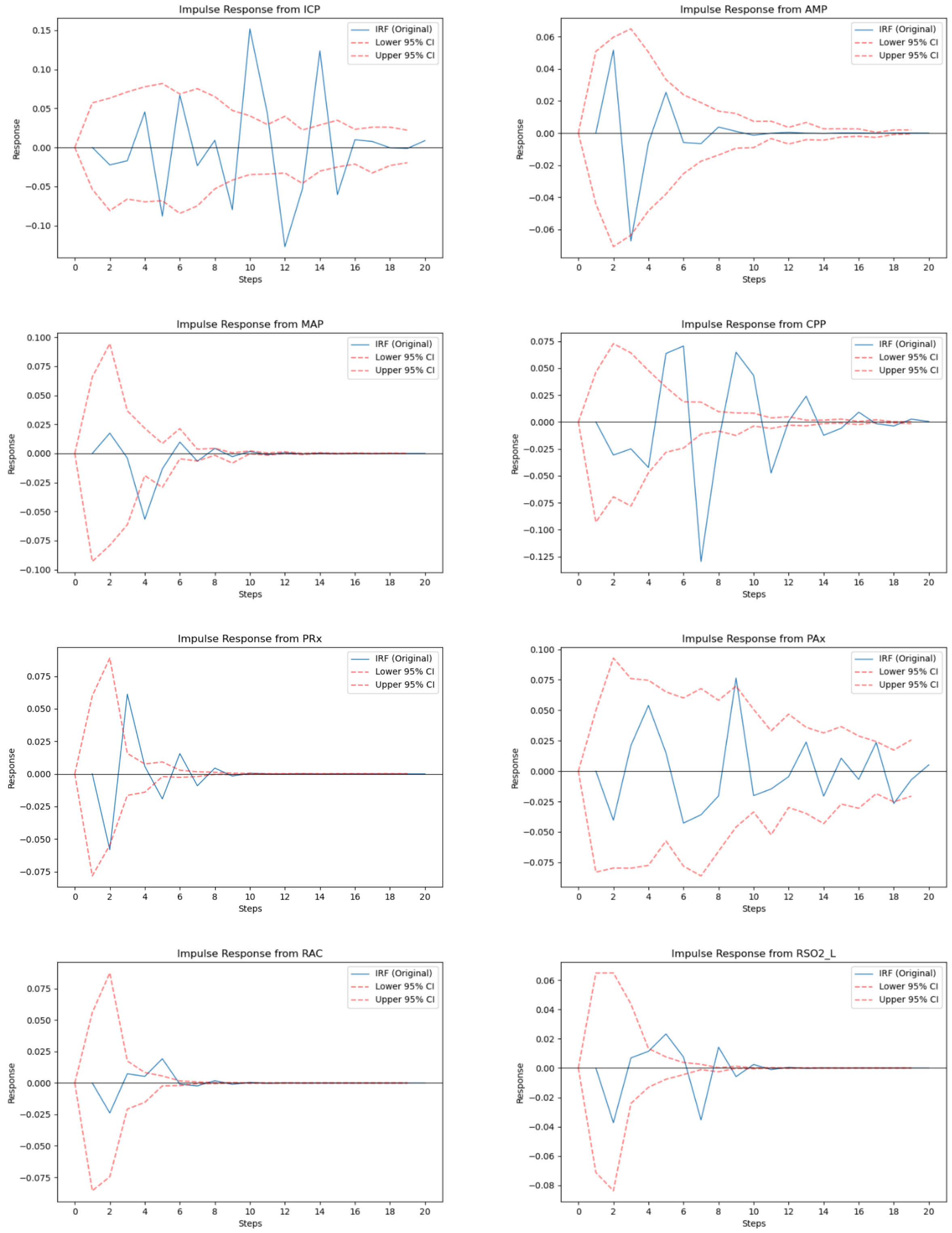

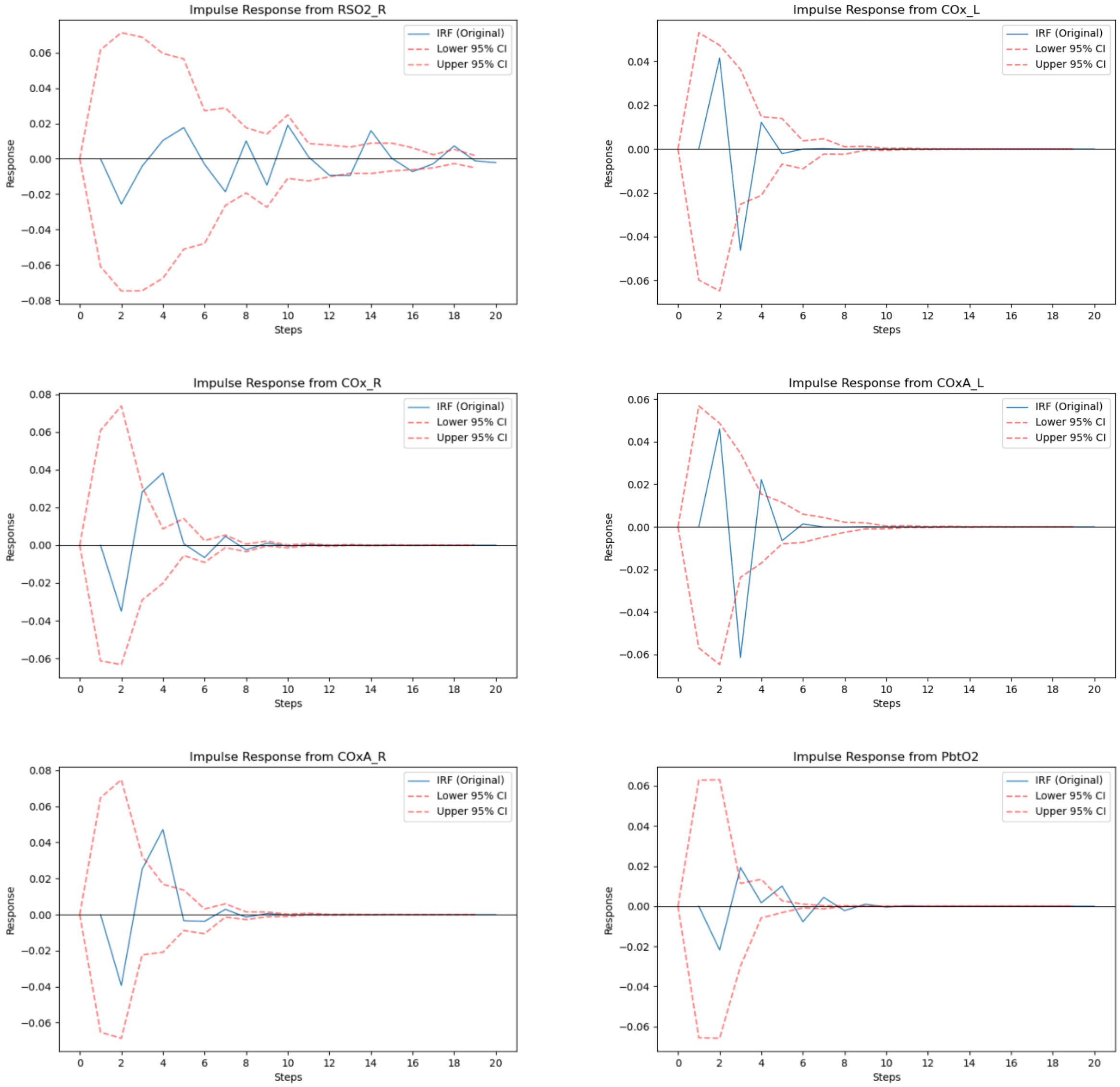

Impulse Response from RSO2_R
Impulse Response from COx_L
Impulse Response from COx_R
Impulse Response from COxA_L
Impulse Response from COxA_R
Impulse Response from PbtO2
IRF (Original)
Lower 95% CI
Upper 95% CI
Response
Steps

Figure S.3 VARIMA IRF plots at 10-minute-by-10-minute resolution for patient CAHR-101

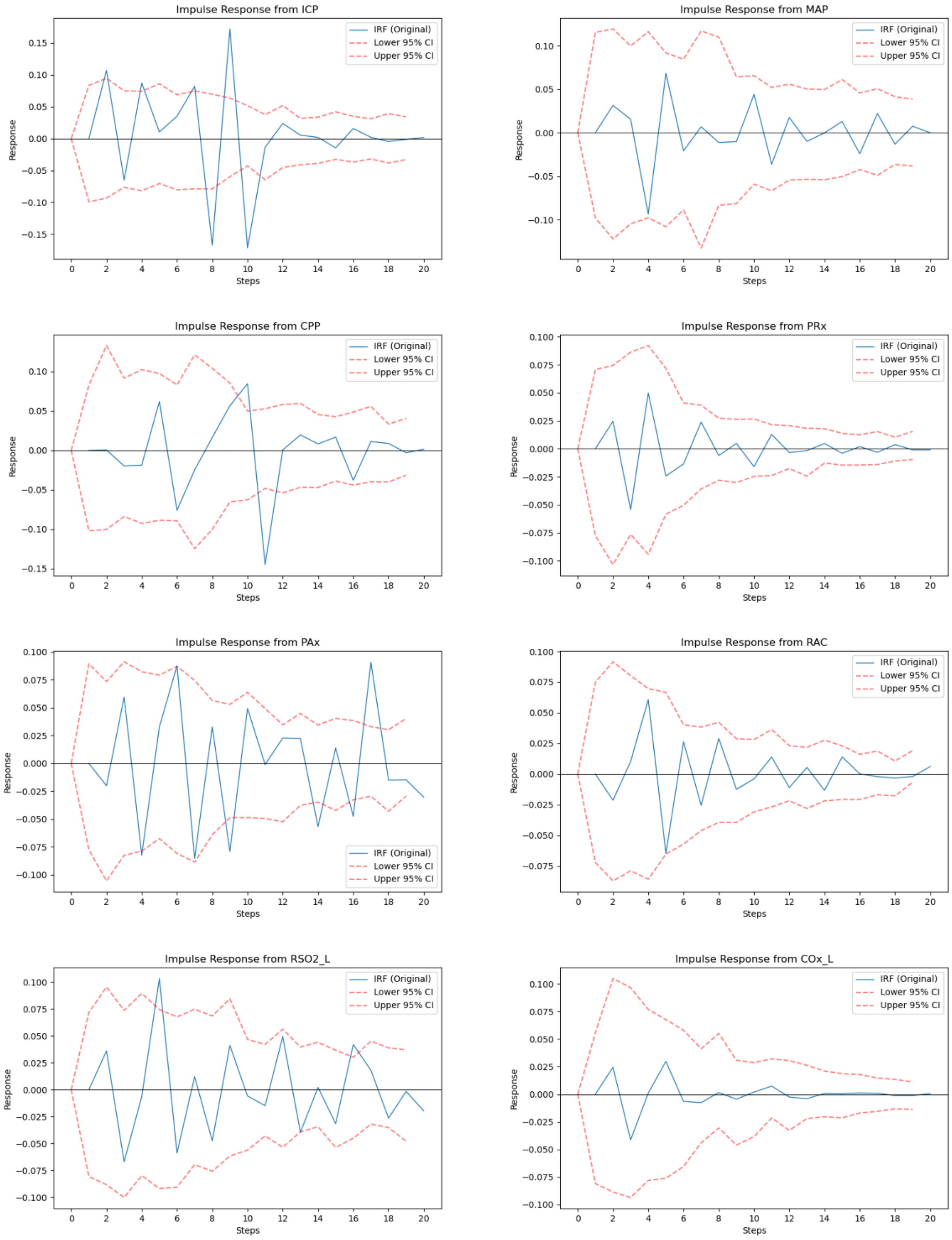

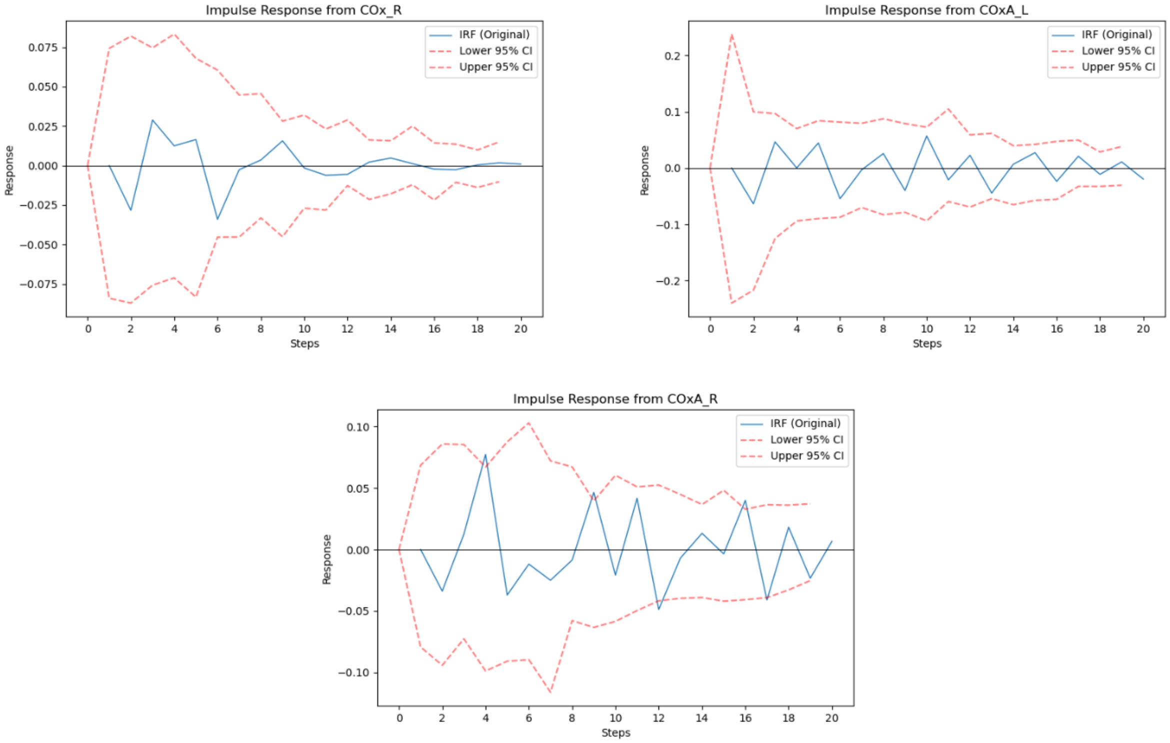

In this figure, the impulse response for AMP, rSO_2_R, $PbtO_2$ is absent because the corresponding VARIMA model failed to fit the data due to an insufficient number of data points, resulting in an error and no output from the analysis.

Table S.2 Responsiveness of RAP to the orthogonal impulse of cerebral physiological parameters across RAP states

**RAP < 0**						
Direction	Minute-by-minute		5-minute-by-5-minute		10-minute-by-10-minute	
	>0.1%	NA	>0.1%	NA	>0.1%	NA
ΔICP→ΔRAP	83.33%(5)	0	16.67%(1)	0	66.67%(4)	0
ΔAMP→ΔRAP	83.33%(5)	0	50.00%(3)	0	50.00%(3)	0
ΔMAP→ΔRAP	100.00%(6)	0	66.67%(4)	0	50.00%(3)	0
ΔCPP→ΔRAP	33.33%(2)	0	16.67%(1)	0	50.00%(3)	0
ΔPRx→ΔRAP	83.33%(5)	0	33.33%(2)	0	50.00%(3)	0
ΔPAx→ΔRAP	100.00%(6)	0	50.00%(3)	0	66.67%(4)	0
ΔRAC→ΔRAP	100.00%(6)	0	50.00%(3)	0	33.33%(2)	0
ΔrSO_2_L→ΔRAP	100.00%(1)	0	0.00%(0)	0	0.00%(0)	0
ΔrSO_2_R→ΔRAP	100.00%(1)	0	0.00%(0)	0	0.00%(0)	0
ΔCOx_L→ΔRAP	100.00%(1)	0	0.00%(0)	0	0.00%(0)	0
ΔCOx_R→ΔRAP	0.00%(0)	0	0.00%(0)	0	0.00%(0)	0
ΔCOx-a_L→ΔRAP	0.00%(0)	0	0.00%(0)	0	0.00%(0)	0
ΔCOx-a_R→ΔRAP	0.00%(0)	0	0.00%(0)	0	0.00%(0)	0
Δ$PbtO_2$→ΔRAP	100.00%(4	0	50.00%(2)	0	25.00%(1)	0
**0 ≤ RAP ≤ 0.4**						
Direction	Minute-by-minute		5-minute-by-5-minute		10-minute-by-10-minute	
	>0.1%	NA	>0.1%	NA	>0.1%	NA
ΔICP→ΔRAP	84.00%(21)	0	64.00%(16)	0	60.00%(15)	0
ΔAMP→ΔRAP	84.00%(21)	0	60.00%(15)	0	52.00%(13)	0

ΔMAP→ΔRAP	80.00%(20)	0	68.00%(17)	0	52.00%(13)	0
ΔCPP→ΔRAP	80.00%(20)	0	80.00%(20)	0	52.00%(13)	0
ΔPRx→ΔRAP	72.00%(18)	0	40.00%(10)	0	44.00%(11)	0
ΔPAx→ΔRAP	80.00%(20)	0	40.00%(10)	0	48.00%(12)	0
ΔRAC→ΔRAP	80.00%(20)	0	52.00%(13)	0	40.00%(10)	0
ΔrSO_2_L→ΔRAP	75.00%(9)	1	41.67%(5)	1	66.67%(8)	1
ΔrSO_2_R→ΔRAP	91.67%(11)	2	66.67%(8)	2	66.67%(8)	2
ΔCOx_L→ΔRAP	58.33%(7)	1	41.67%(5)	1	16.67%(2)	1
ΔCOx_R→ΔRAP	66.67%(8)	0	41.67%(5)	0	25.00%(3)	0
ΔCOx-a_L→ΔRAP	83.33%(10)	0	50.00%(6)	0	41.67%(5)	0
ΔCOx-a_R→ΔRAP	66.67%(8)	0	58.33%(7)	0	41.67%(5)	0
ΔPbtO_2→ΔRAP	100.00%(4)	0	75.00%(3)	0	100.00%(4)	0
**RAP > 0.4**						
Direction	Minute-by-minute		5-minute-by-5-minute		10-minute-by-10-minute	
	>0.1%	NA	>0.1%	NA	>0.1%	NA
ΔICP→ΔRAP	77.30%(269)	0	63.79%(222)	0	64.37%(224)	0
ΔAMP→ΔRAP	74.43%(259)	0	61.78%(215)	0	59.20%(206)	0
ΔMAP→ΔRAP	72.41%(252)	0	67.24%(234)	0	58.62%(204)	0
ΔCPP→ΔRAP	71.26%(248)	0	66.38%(231)	0	56.90%(198)	0
ΔPRx→ΔRAP	78.74%(274)	0	54.31%(189)	0	43.10%(150)	0
ΔPAx→ΔRAP	77.59%(270)	0	52.59%(183)	0	38.51%(134)	0
ΔRAC→ΔRAP	79.31%(276)	0	55.75%(194)	0	41.67%(145)	0
ΔrSO_2_L→ΔRAP	75.19%(97)	13	67.44%(87)	13	61.24%(79)	13
ΔrSO_2_R→ΔRAP	75.40%(95)	14	70.63%(89)	14	57.94%(73)	14
ΔCOx_L→ΔRAP	74.02%(94)	11	40.16%(51)	11	34.65%(44)	11
ΔCOx_R→ΔRAP	76.98%(97)	13	46.03%(58)	13	32.54%(41)	13
ΔCOx-a_L→ΔRAP	80.49%(99)	4	45.53%(56)	4	32.52%(40)	4
ΔCOx-a_R→ΔRAP	81.15%(99)	4	44.26%(54)	4	30.33%(37)	4
ΔPbtO_2→ΔRAP	60.19%(65)	0	66.67%(72)	0	62.96%(68)	0

Supplementary F: Application of Granger causality testing

This supplemantray includes tables summarizing the results of the sub-group Granger causality analyses across the three data resolutions. For each RAP–X pair (where X denotes another cerebral physiological parameter), four categories are presented: ΔX→ΔRAP, indicating RAP's response to an orthogonal impulse from X; ΔRAP→ΔX, indicating the reverse relationship; NS, denoting the number of cases with non-significant results; and NA, representing cases where results could not be obtained due to insufficient data. To calculate the percentage values in these tables, the NA cases were excluded from the total number of patients.

Δ, first-order differenced; AMP, pulse amplitude of ICP; COx_L, cerebral oxygenation index of left hemisphere; COx_R, cerebral oxygenation index of right hemisphere; COx-a_L, COx with ABP of left hemisphere; COx-a_R, COx with ABP of the right hemisphere; CPP, cerebral perfusion pressure; ICP, intracranial pressure; MAP, mean arterial pressure; NA, not applicable, NS, not significant; PAx, pulse amplitude index; $PbtO_2$, brain tissue oxygenation ; PRx, pressure reactivity index; RAC, a cerebral autoregulation index; RAP, index of cerebral compensatory reserve; rSO_2_L, regional cerebral oxygen saturation of left hemisphere; rSO_2_R, regional cerebral oxygen saturation of the right hemisphere.

Table S.1 Granger causality testing across the resolutions for RAP < 0 state

Parameters	Direction	Minute-by-minute	5-minute-by-5-minute	10-minute-by-10-minute
ΔICP & ΔRAP	ΔICP→ΔRAP	7.59%(28)	9.82%(32)	4.78%(12)
	ΔRAP→ΔICP	14.09%(52)	7.67%(25)	9.16%(23)
	NS	78.32%(289)	82.52%(269)	86.06%(216)
	NA	10	53	128
ΔAMP& ΔRAP	ΔAMP→ΔRAP	6.5%(24)	6.12%(20)	5.95%(15)
	ΔRAP→ΔAMP	10.3%(38)	4.89%(16)	5.56%(14)
	NS	83.2%(307)	88.99%(291)	88.49%(223)
	NA	10	52	127
ΔMAP& ΔRAP	ΔMAP→ΔRAP	4.05%(15)	7.12%(23)	5.2%(13)
	ΔRAP→ΔMAP	10.27%(38)	5.88%(19)	5.6%(14)
	NS	85.68%(317)	87.0%(281)	89.2%(223)
	NA	9	56	129
ΔCPP& ΔRAP	ΔCPP→ΔRAP	6.23%(23)	8.07%(26)	6.05%(15)
	ΔRAP→ΔCPP	10.03%(37)	7.14%(23)	6.05%(15)
	NS	83.74%(309)	84.78%(273)	87.9%(218)
	NA	10	57	131
ΔPRx & ΔRAP	ΔPRx→ΔRAP	10.05%(37)	4.02%(13)	4.82%(12)
	ΔRAP→ΔPRx	9.24%(34)	5.57%(18)	9.64%(24)
	NS	80.71%(297)	90.4%(292)	85.54%(213)
	NA	11	56	130
ΔPAx & ΔRAP	ΔPAx→ΔRAP	10.33%(38)	5.26%(17)	8.03%(20)
	ΔRAP→ΔPAx	10.33%(38)	7.74%(25)	7.63%(19)
	NS	79.35%(292)	87.0%(281)	84.34%(210)
	NA	11	56	130
ΔRAC & ΔRAP	ΔRAC→ΔRAP	13.86%(51)	5.26%(17)	7.23%(18)
	ΔRAP→ΔRAC	9.24%(34)	7.12%(23)	5.22%(13)
	NS	76.9%(283)	87.62%(283)	87.55%(218)
	NA	11	56	130
ΔrSO_2_L & ΔRAP	ΔrSO_2_L→ΔRAP	5.88%(8)	5.88%(7)	7.59%(6)
	ΔRAP→ΔrSO_2_L	9.56%(13)	5.04%(6)	3.8%(3)

	NS	84.56%(115)	89.08%(106)	88.61%(70)
	NA	20	37	77
ΔrSO$_2$_R & ΔRAP	ΔrSO$_2$_R→ΔRAP	7.41%(10)	4.31%(5)	7.89%(6)
	ΔRAP→ΔrSO$_2$_L	11.11%(15)	4.31%(5)	6.58%(5)
	NS	81.48%(110)	91.38%(106)	85.53%(65)
	NA	20	39	79
ΔCOx_L & ΔRAP	ΔCOx_L→ΔRAP	7.52%(10)	5.93%(7)	5.41%(4)
	ΔRAP→ΔCOx_L	9.02%(12)	2.54%(3)	5.41%(4)
	NS	83.46%(111)	91.53%(108)	89.19%(66)
	NA	19	34	78
ΔCOx_R & ΔRAP	ΔCOx_R→ΔRAP	8.27%(11)	10.43%(12)	9.33%(7)
	ΔRAP→ΔCOx_R	6.77%(9)	4.35%(5)	4.0%(3)
	NS	84.96%(113)	85.22%(98)	86.67%(65)
	NA	19	37	77
ΔCOx-a_L & ΔRAP	ΔCOx-a_L→ΔRAP	5.43%(7)	7.89%(9)	6.76%(5)
	ΔRAP→ΔCOx-a_L	10.08%(13)	4.39%(5)	8.11%(6)
	NS	84.5%(109)	87.72%(100)	85.14%(63)
	NA	11	26	66
ΔCOx-a_R & ΔRAP	ΔCOx-a_R→ΔRAP	6.2%(8)	4.5%(5)	9.59%(7)
	ΔRAP→ΔCOx-a_R	8.53%(11)	7.21%(8)	8.22%(6)
	NS	85.27%(110)	88.29%(98)	82.19%(60)
	NA	11	29	67
ΔPbtO$_2$ & ΔRAP	ΔPbtO$_2$→ΔRAP	4.59%(5)	12.5%(12)	6.41%(5)
	ΔRAP→ΔPbtO$_2$	8.26%(9)	4.17%(4)	8.97%(7)
	NS	87.16%(95)	83.33%(80)	84.62%(66)
	NA	7	20	38

Table S.2 Granger causality testing across the resolutions for $0 \leq RAP \leq 0.4$ state

Parameters	Direction	Minute-by-minute	5-minute-by-5-minute	10-minute-by-10-minute
ΔICP & ΔRAP	ΔICP→ΔRAP	7.59%(28)	9.82%(32)	4.78%(12)
	ΔRAP→ΔICP	14.09%(52)	7.67%(25)	9.16%(23)
	NS	78.32%(289)	82.52%(269)	86.06%(216)
	NA	10	53	128
ΔAMP& ΔRAP	ΔAMP→ΔRAP	6.5%(24)	6.12%(20)	5.95%(15)
	ΔRAP→ΔAMP	10.3%(38)	4.89%(16)	5.56%(14)
	NS	83.2%(307)	88.99%(291)	88.49%(223)
	NA	10	52	127
ΔMAP& ΔRAP	ΔMAP→ΔRAP	4.05%(15)	7.12%(23)	5.2%(13)
	ΔRAP→ΔMAP	10.27%(38)	5.88%(19)	5.6%(14)
	NS	85.68%(317)	87.0%(281)	89.2%(223)
	NA	9	56	129
ΔCPP& ΔRAP	ΔCPP→ΔRAP	6.23%(23)	8.07%(26)	6.05%(15)
	ΔRAP→ΔCPP	10.03%(37)	7.14%(23)	6.05%(15)
	NS	83.74%(309)	84.78%(273)	87.9%(218)
	NA	10	57	131
ΔPRx & ΔRAP	ΔPRx→ΔRAP	10.05%(37)	4.02%(13)	4.82%(12)
	ΔRAP→ΔPRx	9.24%(34)	5.57%(18)	9.64%(24)
	NS	80.71%(297)	90.4%(292)	85.54%(213)
	NA	11	56	130
ΔPAx & ΔRAP	ΔPAx→ΔRAP	10.33%(38)	5.26%(17)	8.03%(20)
	ΔRAP→ΔPAx	10.33%(38)	7.74%(25)	7.63%(19)

	NS	79.35%(292)	87.0%(281)	84.34%(210)
	NA	11	56	130
ΔRAC & ΔRAP	ΔRAC→ΔRAP	13.86%(51)	5.26%(17)	7.23%(18)
	ΔRAP→ΔRAC	9.24%(34)	7.12%(23)	5.22%(13)
	NS	76.9%(283)	87.62%(283)	87.55%(218)
	NA	11	56	130
ΔrSO$_2$_L & ΔRAP	ΔrSO$_2$_L→ΔRAP	5.88%(8)	5.88%(7)	7.59%(6)
	ΔRAP→ΔrSO$_2$_L	9.56%(13)	5.04%(6)	3.8%(3)
	NS	84.56%(115)	89.08%(106)	88.61%(70)
	NA	20	37	77
ΔrSO$_2$_R & ΔRAP	ΔrSO$_2$_R→ΔRAP	7.41%(10)	4.31%(5)	7.89%(6)
	ΔRAP→ΔrSO$_2$_L	11.11%(15)	4.31%(5)	6.58%(5)
	NS	81.48%(110)	91.38%(106)	85.53%(65)
	NA	20	39	79
ΔrSO$_2$_R & ΔRAP	ΔCOx_L→ΔRAP	7.52%(10)	5.93%(7)	5.41%(4)
	ΔRAP→ΔCOx_L	9.02%(12)	2.54%(3)	5.41%(4)
	NS	83.46%(111)	91.53%(108)	89.19%(66)
	NA	19	34	78
ΔCOx_R & ΔRAP	ΔCOx_R→ΔRAP	8.27%(11)	10.43%(12)	9.33%(7)
	ΔRAP→ΔCOx_R	6.77%(9)	4.35%(5)	4.0%(3)
	NS	84.96%(113)	85.22%(98)	86.67%(65)
	NA	19	37	77
ΔCOx-a_L & ΔRAP	ΔCOx-a_L→ΔRAP	5.43%(7)	7.89%(9)	6.76%(5)
	ΔRAP→ΔCOx-a_L	10.08%(13)	4.39%(5)	8.11%(6)
	NS	84.5%(109)	87.72%(100)	85.14%(63)
	NA	11	26	66
ΔCOx-a_R & ΔRAP	ΔCOx-a_R→ΔRAP	6.2%(8)	4.5%(5)	9.59%(7)
	ΔRAP→ΔCOx-a_R	8.53%(11)	7.21%(8)	8.22%(6)
	NS	85.27%(110)	88.29%(98)	82.19%(60)
	NA	11	29	67
ΔPbtO$_2$ & ΔRAP	ΔPbtO$_2$→ΔRAP	4.59%(5)	12.5%(12)	6.41%(5)
	ΔRAP→ΔPbtO$_2$	8.26%(9)	4.17%(4)	8.97%(7)
	NS	87.16%(95)	83.33%(80)	84.62%(66)
	NA	7	20	38

Table S.3 Granger causality testing across the resolutions for RAP > 0.4 state

Parameters	Direction	Minute-by-minute	5-minute-by-5-minute	10-minute-by-10-minute
ΔICP & ΔRAP	ΔICP→ΔRAP	17.94%(68)	30.5%(115)	26.67%(100)
	ΔRAP→ΔICP	27.18%(103)	17.51%(66)	15.73%(59)
	NS	54.88%(208)	51.99%(196)	57.6%(216)
	NA	0	2	4
ΔAMP& ΔRAP	ΔAMP→ΔRAP	14.25%(54)	44.03%(166)	31.73%(119)
	ΔRAP→ΔAMP	41.16%(156)	15.12%(57)	17.87%(67)
	NS	44.59%(169)	40.85%(154)	50.4%(189)
	NA	0	2	4
ΔMAP& ΔRAP	ΔMAP→ΔRAP	26.91%(102)	42.97%(162)	26.47%(99)
	ΔRAP→ΔMAP	20.58%(78)	11.94%(45)	9.09%(34)
	NS	52.51%(199)	45.09%(170)	64.44%(241)
	NA	0	2	5
ΔCPP& ΔRAP	ΔCPP→ΔRAP	25.07%(95)	45.62%(172)	29.95%(112)
	ΔRAP→ΔCPP	22.96%(87)	12.47%(47)	12.83%(48)

	NS	51.98%(197)	41.91%(158)	57.22%(214)
	NA	0	2	5
ΔPRx & ΔRAP	ΔPRx→ΔRAP	38.79%(147)	14.85%(56)	12.27%(46)
	ΔRAP→ΔPRx	17.41%(66)	16.98%(64)	15.47%(58)
	NS	43.8%(166)	68.17%(257)	72.27%(271)
	NA	0	2	4
ΔPAx & ΔRAP	ΔPAx→ΔRAP	39.58%(150)	11.67%(44)	10.13%(38)
	ΔRAP→ΔPAx	21.11%(80)	19.63%(74)	14.4%(54)
	NS	39.31%(149)	68.7%(259)	75.47%(283)
	NA	0	2	4
ΔRAC & ΔRAP	ΔRAC→ΔRAP	45.38%(172)	13.26%(50)	13.33%(50)
	ΔRAP→ΔRAC	19.26%(73)	22.81%(86)	15.73%(59)
	NS	35.36%(134)	63.93%(241)	70.93%(266)
	NA	0	2	4
ΔrSO_2_L & ΔRAP	ΔrSO_2_L→ΔRAP	17.14%(24)	20.44%(28)	8.09%(11)
	ΔRAP→ΔrSO_2_L	24.29%(34)	15.33%(21)	17.65%(24)
	NS	58.57%(82)	64.23%(88)	74.26%(101)
	NA	16	19	20
ΔrSO_2_R & ΔRAP	ΔrSO_2_R→ΔRAP	12.32%(17)	20.59%(28)	6.67%(9)
	ΔRAP→ΔrSO_2_L	28.26%(39)	13.97%(19)	7.41%(10)
	NS	59.42%(82)	65.44%(89)	85.93%(116)
	NA	17	19	20
ΔrSO_2_R & ΔRAP	ΔCOx_L→ΔRAP	10.79%(15)	5.88%(8)	8.21%(11)
	ΔRAP→ΔCOx_L	25.9%(36)	23.53%(32)	18.66%(25)
	NS	63.31%(88)	70.59%(96)	73.13%(98)
	NA	13	16	18
ΔCOx_R & ΔRAP	ΔCOx_R→ΔRAP	15.22%(21)	5.15%(7)	5.97%(8)
	ΔRAP→ΔCOx_R	17.39%(24)	31.62%(43)	17.16%(23)
	NS	67.39%(93)	63.24%(86)	76.87%(103)
	NA	14	16	18
ΔCOx-a_L & ΔRAP	ΔCOx-a_L→ΔRAP	11.11%(15)	8.33%(11)	10.0%(13)
	ΔRAP→ΔCOx-a_L	27.41%(37)	16.67%(22)	19.23%(25)
	NS	61.48%(83)	75.0%(99)	70.77%(92)
	NA	5	8	10
ΔCOx-a_R & ΔRAP	ΔCOx-a_R→ΔRAP	13.43%(18)	6.06%(8)	6.92%(9)
	ΔRAP→ΔCOx-a_R	23.88%(32)	19.7%(26)	13.85%(18)
	NS	62.69%(84)	74.24%(98)	79.23%(103)
	NA	6	8	10
$\Delta PbtO_2$ & ΔRAP	$\Delta PbtO_2$→ΔRAP	15.93%(18)	14.29%(16)	11.93%(13)
	ΔRAP→$\Delta PbtO_2$	21.24%(24)	19.64%(22)	11.93%(13)
	NS	62.83%(71)	66.07%(74)	76.15%(83)
	NA	3	4	7

Supplementary G: Application of cross-correlation analysis

This supplemantray presents tables summarizing the cross-correlation analysis results across three data resolutions at the sub-group levels. Each table reports the median of the maximum correlation values along with their interquartile range (IQR), as well as the median of the absolute lag values (with IQR) at which peak correlation occurred. Instances labelled as NA indicate patient examples where the analysis could not be performed due to insufficient data.

Δ, first-order differenced; AMP, pulse amplitude of ICP; COx_L, cerebral oxygenation index of left hemisphere; COx_R, cerebral oxygenation index of right hemisphere; COx-a_L, COx with ABP of left hemisphere; COx-a_R, COx with ABP of the right hemisphere; CPP, cerebral perfusion pressure; ICP, intracranial pressure; MAP, mean arterial pressure; NA, not applicable; PAx, pulse amplitude index; $PbtO_2$*, brain tissue oxygenation ; PRx, pressure reactivity index; RAC, a cerebral autoregulation index; RAP, index of cerebral compensatory reserve;* rSO_2*_L, regional cerebral oxygen saturation of left hemisphere;* rSO_2*_R, regional cerebral oxygen saturation of the right hemisphere.*

Table S.1 ΔRAP-ΔX cross-correlation analysis across the resolutions for RAP < 0 state

Parameter pair	Minute-by-minute			5-minute-by-5-minute			10-minute-by-10-minute		
	Maximum correlation	Maximum lag	NA	Maximum correlation	Maximum lag	NA	Maximum correlation	Maximum lag	NA
ΔRAP-ΔICP	0.192(0.13—0.274)	4.0(0.0—10.0)	3	0.364(0.262—0.479)	5.0(1.0—10.0)	18	0.468(0.362—0.576)	2.0(0.0—6.0)	58
ΔRAP-ΔAMP	0.175(0.122—0.288)	5.0(1.0—11.0)	3	0.37(0.266—0.5)	5.0(1.0—11.0)	18	0.473(0.359—0.56)	2.0(0.0—5.0)	57
ΔRAP-ΔMAP	0.171(0.115—0.263)	6.0(2.0—13.25)	3	0.37(0.268—0.483)	5.0(2.0—9.0)	17	0.448(0.321—0.552)	2.0(0.0—7.0)	56
ΔRAP-ΔCPP	0.167(0.116—0.266)	6.0(2.0—14.0)	3	0.365(0.257—0.483)	4.0(1.0—10.0)	18	0.455(0.337—0.537)	2.0(0.0—7.0)	62
ΔRAP-ΔPRx	0.196(0.132—0.306)	3.0(0.0—11.0)	3	0.364(0.283—0.5)	4.0(1.0—9.0)	16	0.478(0.359—0.548)	2.0(0.0—6.0)	58
ΔRAP-ΔPAx	0.245(0.153—0.352)	0.0(0.0—7.0)	3	0.377(0.293—0.5)	2.0(0.0—8.0)	16	0.479(0.363—0.554)	1.0(0.0—5.0)	58
ΔRAP-ΔRAC	0.224(0.151—0.349)	1.0(0.0—8.0)	3	0.391(0.296—0.5)	2.0(0.0—7.0)	16	0.476(0.365—0.563)	1.0(0.0—5.0)	58
ΔRAP-ΔrSO_2_L	0.205(0.153—0.316)	7.0(2.0—13.75)	14	0.415(0.316—0.528)	3.0(1.0—8.0)	20	0.5(0.419—0.602)	1.0(0.0—4.25)	28
ΔRAP-ΔrSO_2_R	0.208(0.144—0.322)	7.0(2.0—14.75)	13	0.394(0.285—0.498)	4.0(1.0—8.0)	18	0.5(0.408—0.568)	1.0(0.0—5.0)	30
ΔRAP-ΔCOx_L	0.226(0.16—0.311)	7.0(1.0—15.0)	11	0.445(0.362—0.51)	3.0(1.0—7.75)	18	0.5(0.42—0.597)	1.0(0.0—3.0)	28

ΔRAP-ΔCOx_R	0.217(0.156—0.311)	7.0(2.0—14.0)	10	0.44(0.329—0.528)	4.0(1.0—8.0)	18	0.5(0.433—0.576)	1.0(0.0—4.0)	31
ΔRAP-ΔCOx-a_L	0.226(0.155—0.316)	5.0(1.0—11.0)	9	0.429(0.369—0.517)	3.0(1.0—6.0)	16	0.508(0.438—0.596)	1.0(0.0—3.0)	26
ΔRAP-ΔCOx-a_R	0.218(0.157—0.313)	7.0(2.75—12.0)	8	0.435(0.343—0.514)	3.0(1.0—8.0)	16	0.5(0.398—0.602)	1.0(0.0—4.0)	29
ΔRAP-$\Delta PbtO_2$	0.157(0.106—0.251)	6.0(3.0—12.0)	4	0.37(0.277—0.488)	5.0(1.0—11.0)	8	0.467(0.372—0.563)	2.0(1.0—6.0)	19

Table S.2 ΔRAP-ΔX cross-correlation analysis across the resolutions for 0 ≤ RAP ≤ 0.4 state

Parameter pair	Minute-by-minute			5-minute-by-5-minute			10-minute-by-10-minute		
	Maximum correlation	Maximum lag	NA	Maximum correlation	Maximum lag	NA	Maximum correlation	Maximum lag	NA
ΔRAP-ΔICP	0.117(0.086—0.176)	7.0(2.0—13.0)	3	0.241(0.166—0.352)	7.0(2.0—12.0)	18	0.309(0.226—0.406)	6.0(2.0—12.0)	58
ΔRAP-ΔAMP	0.124(0.086—0.179)	7.0(2.0—13.0)	3	0.245(0.17—0.327)	6.0(2.0—12.0)	18	0.313(0.225—0.42)	5.0(1.0—12.0)	57
ΔRAP-ΔMAP	0.126(0.087—0.186)	9.0(3.0—14.0)	3	0.249(0.176—0.357)	7.0(2.0—13.0)	17	0.313(0.228—0.418)	6.0(2.0—11.0)	56
ΔRAP-ΔCPP	0.127(0.086—0.188)	9.0(4.0—14.0)	3	0.245(0.179—0.341)	6.0(2.0—13.0)	18	0.315(0.234—0.413)	6.0(2.0—11.0)	62
ΔRAP-ΔPRx	0.139(0.094—0.218)	4.0(0.0—11.75)	3	0.265(0.2—0.364)	5.0(1.0—12.0)	16	0.326(0.242—0.432)	5.0(2.0—11.0)	58
ΔRAP-ΔPAx	0.152(0.108—0.232)	2.0(0.0—10.0)	3	0.264(0.192—0.379)	5.0(1.0—11.0)	16	0.326(0.236—0.444)	4.0(1.0—9.0)	58
ΔRAP-ΔRAC	0.14(0.097—0.226)	5.0(0.0—12.0)	3	0.262(0.188—0.373)	6.0(1.0—13.0)	16	0.34(0.244—0.454)	4.0(1.0—10.0)	58
ΔRAP-ΔrSO_2_L	0.136(0.098—0.227)	8.0(3.0—14.0)	14	0.294(0.216—0.377)	6.0(3.0—13.5)	20	0.332(0.256—0.468)	5.0(2.0—11.0)	28
ΔRAP-ΔrSO_2_R	0.141(0.106—0.228)	9.0(3.0—15.0)	13	0.271(0.196—0.356)	6.0(2.0—14.0)	18	0.325(0.255—0.446)	5.0(1.0—11.0)	30
ΔRAP-ΔCOx_L	0.142(0.109—0.241)	8.0(4.0—13.0)	11	0.301(0.232—0.416)	7.0(2.0—14.0)	18	0.363(0.297—0.473)	5.0(2.0—11.0)	28
ΔRAP-ΔCOx_R	0.16(0.104—0.232)	8.0(4.0—14.0)	10	0.297(0.222—0.402)	6.0(2.0—13.0)	18	0.361(0.288—0.481)	6.0(2.0—12.0)	31
ΔRAP-ΔCOx-a_L	0.147(0.114—0.26)	8.0(4.0—13.0)	9	0.287(0.226—0.408)	5.0(2.0—11.5)	16	0.364(0.28—0.467)	5.0(2.0—9.0)	26

ΔRAP-ΔCOx-a_R	0.157(0.103—0.231)	8.0(3.0—14.0)	8	0.295(0.211—0.424)	5.0(2.0—9.0)	16	0.367(0.277—0.45)	5.0(2.0—10.0)	29
ΔRAP-ΔPbtO$_2$	0.126(0.085—0.182)	6.0(3.0—12.0)	4	0.254(0.178—0.349)	5.0(2.0—12.25)	8	0.32(0.228—0.427)	6.0(2.0—10.0)	19

Table S.3 ΔRAP-ΔX cross-correlation analysis across the resolutions for RAP > 0.4 state

Parameter pair	Minute-by-minute			5-minute-by-5-minute			10-minute-by-10-minute		
	Maximum correlation	Maximum lag	NA	Maximum correlation	Maximum lag	NA	Maximum correlation	Maximum lag	NA
ΔRAP-ΔICP	0.07(0.052—0.109)	3.0(0.0—5.0)	0	0.129(0.094—0.185)	1.0(0.0—6.0)	0	0.164(0.117—0.236)	1.0(0.0—9.0)	1
ΔRAP-ΔAMP	0.094(0.069—0.131)	0.0(0.0—4.0)	0	0.145(0.101—0.215)	1.0(0.0—5.0)	0	0.192(0.127—0.271)	1.0(0.0—6.0)	1
ΔRAP-ΔMAP	0.069(0.048—0.11)	2.0(0.0—8.0)	0	0.153(0.108—0.234)	1.0(0.0—6.0)	0	0.196(0.149—0.313)	0.0(0.0—6.0)	0
ΔRAP-ΔCPP	0.069(0.048—0.104)	2.0(0.0—8.0)	0	0.149(0.103—0.216)	1.0(0.0—6.0)	0	0.192(0.142—0.302)	1.0(0.0—6.0)	1
ΔRAP-ΔPRx	0.124(0.075—0.195)	0.0(0.0—1.0)	0	0.17(0.12—0.254)	0.0(0.0—6.0)	0	0.211(0.151—0.308)	1.0(0.0—8.0)	0
ΔRAP-ΔPAx	0.101(0.063—0.158)	0.0(0.0—1.0)	0	0.143(0.1—0.219)	0.0(0.0—7.0)	0	0.179(0.129—0.278)	1.0(0.0—9.0)	0
ΔRAP-ΔRAC	0.184(0.114—0.267)	0.0(0.0—0.0)	0	0.252(0.158—0.349)	0.0(0.0—1.0)	0	0.291(0.206—0.412)	0.0(0.0—2.0)	0
ΔRAP-ΔrSO$_2$_L	0.084(0.056—0.121)	5.0(1.0—13.0)	9	0.146(0.106—0.236)	3.0(0.0—11.0)	11	0.193(0.13—0.284)	6.0(1.0—14.0)	11
ΔRAP-ΔrSO$_2$_R	0.075(0.05—0.124)	4.0(1.0—11.0)	10	0.14(0.1—0.221)	4.0(1.0—13.5)	12	0.184(0.131—0.264)	6.0(1.0—13.0)	12
ΔRAP-ΔCOx_L	0.077(0.052—0.11)	3.5(0.0—10.0)	6	0.162(0.101—0.213)	5.5(1.0—13.0)	8	0.212(0.145—0.298)	5.0(0.0—10.0)	8
ΔRAP-ΔCOx_R	0.071(0.049—0.115)	6.0(1.0—14.0)	7	0.146(0.095—0.231)	5.0(1.0—14.0)	9	0.19(0.15—0.271)	6.0(1.0—16.0)	9
ΔRAP-ΔCOx-a_L	0.072(0.05—0.114)	3.0(1.0—10.25)	4	0.149(0.097—0.22)	6.0(1.0—12.0)	6	0.2(0.143—0.28)	4.0(1.0—10.0)	6
ΔRAP-ΔCOx-a_R	0.074(0.044—0.114)	5.0(1.0—11.0)	5	0.14(0.09—0.23)	5.0(1.0—12.0)	7	0.178(0.132—0.278)	5.0(0.0—14.0)	7
ΔRAP-ΔPbtO$_2$	0.063(0.044—0.092)	5.0(1.0—10.75)	2	0.123(0.088—0.176)	4.0(1.0—13.0)	3	0.159(0.111—0.223)	6.0(1.0—14.0)	3
